# Physiological reactions to acute stressors and subjective stress during daily life: A systematic review on ecological momentary assessment (EMA) studies

**DOI:** 10.1371/journal.pone.0271996

**Published:** 2022-07-27

**Authors:** Jeannette Weber, Peter Angerer, Jennifer Apolinário-Hagen

**Affiliations:** Institute of Occupational-, Social- and Environmental Medicine, Centre for Health and Society, Medical Faculty, Heinrich-Heine-University Düsseldorf, Düsseldorf, Germany; University of Colorado Denver, UNITED STATES

## Abstract

**Objective:**

This review aims to provide an overview of ecological momentary assessment (EMA) studies analyzing stress reactivity during daily life in terms of direct and moderated influence of acute stress on physiological responses.

**Materials and methods:**

A systematic literature search was performed on November 29, 2021 using Web of Science, MEDLINE and PsycINFO to identify prospective EMA studies targeting acute stressors or stress under naturalistic conditions, without restrictions of publication date or population. Study quality was assessed for multiple EMA-specific sources of bias.

**Results:**

Out of 4285 non-duplicate records, 107 publications involving 104 unique studies were included. The majority of studies assessed acute physiological stress responses primarily through salivary cortisol (n = 59) and cardiovascular outcomes (n = 32). Most studies performed at least three measurements per day (n = 59), and had a moderate risk of recall bias (n = 68) and confounding (n = 85). Fifty-four studies reported a compliance of ≥80%. Direct, non-moderated positive associations were observed between acute stress exposure and concurrent cortisol levels (44%, n = 11/25), systolic (44%, 8/18) and diastolic blood pressure (53%, 8/15) and heart rate (53%, 9/17). Several inter- and intra-individual moderators were identified, such as age, gender, health status, chronic stress, work-related resources, physical activity and stress coping indicators.

**Conclusions:**

About half of the reviewed EMA studies demonstrated direct associations between everyday acute stress exposure and physiological responses, including increased cortisol levels, blood pressure and heart rate. Results further suggested various moderator variables that could help develop tailored prevention strategies and identify groups at higher risk for dysfunctional stress responses.

**Registration:**

PROSPERO—Reg.-No.: PROSPERO 2020 CRD42020163178.

## Introduction

### Rationale

Psychosocial stress represents a major risk factor for both mental and physical health as repeated, longer or enduring exposure has been demonstrated being associated with several negative health outcomes including cardiovascular diseases, diabetes as well as various mental disorders [1–3]. Stress biology provides the ability to cope with psychosocial stress; the term allostasis refers to the active biological process of adapting and maintaining stability [4]. According to McEwen: "Through allostasis, the autonomic nervous system, the hypothalamic–pituitary–adrenal (HPA) axis, and the cardiovascular, metabolic, and immune systems protect the body by responding to internal and external stress. The price of this accommodation to stress can be allostatic load, which is the wear and tear that results from chronic overactivity or underactivity of allostatic systems” [4]. Increased allostatic load due to long-lasting, chronic or repetitive stimulation of physiological stress responses has been discussed as an underlying mechanism for the relationships between stress and disease [4]. One tool to assess allostatic load is the allostatic load index (ALI). This index captures primary indicators of the biological stress response, originally such as dehydroepiandrosterone sulfate (DHEA-S) and urinary epinephrine, norepinephrine, and cortisol, as well as secondary outcomes: systolic and diastolic blood pressure, total cholesterol, high-density lipoprotein (HDL), glycohemoglobin (HbA1c) and waist-to-hip ratio (WHR). Other compositions and metrics have also been proposed [5, 6].

Most individuals are occasionally or recurrently confronted with acute stressors across life domains like work or private life [7]. Acute stressors typically refer to short-term events [8], which usually lead to acute subjective and physiological stress responses as immediate reaction to the appraisal that one’s resources or capabilities are insufficient to cope with the demands of this event according to transactional stress theory [9]. Overall, psychosocial stressors play an important role in occurrence of acute stress responses. Amongst laboratory stressors, psychosocial stressors were found to induce the most excessive immediate physiological stress responses of the hypothalamic-pituitary-adrenal (HPA) axis when perceived social-evaluative threat, novelty, uncontrollability and ambivalence of social situations are involved [10]. In contrast, cortisol responses are less apparent to passive physical laboratory stressors such as the cold pressure test [11, 12]. Acute stress responses are thus grounded on actual, perceived or anticipated threat for the homeostasis or well-being. They are accompanied by a range of immediately measurable physiological responses mediated by the brain stem limbic region and hypothalamus aiming at regulating the neuroendocrine and automatic stress system [13]. This involves an immediate activation of the HPA axis, the autonomic nervous system (ANS) and the immune system.

To increase knowledge on short-term stress responses during daily life, ecological momentary assessment (EMA) approaches, also known as experience sampling and diary studies, have been increasingly applied in recent years [14, 15]. EMA might help to identify those stressors causing physiological stress reactions in daily life and could thus contribute to the prediction of future disease risk and development of tailored interventions based on specific risk profiles. For EMA, suitable outcomes of the stress response of ALI biomarkers mentioned above are those that react within minutes, hours or from day to day and that can be collected without external help. For the HPA system this may be cortisol in saliva or urine, for the ANS heart rate (HR), heart rate variability (HRV), ambulatory blood pressure (ABP), electrodermal acitivity (EDA), alpha-amylase in saliva and others. Those biomarkers are briefly described below.

Cortisol, released from the adrenal cortex to increase the level of blood glucose, increases within minutes after exposure to an acute stressor and the accompanying immediate emotional response, and reaches the maximum after 20–40 minutes [16]. Cortisol levels follow a strong diurnal course with a sharp rise in the morning, the cortisol awakening response (CAR), and subsequent decline over the day [17, 18]. A flatter decline of this subsequent diurnal cortisol slope has repeatedly been associated with chronic psychosocial stress but also with day-to-day variation of negative experiences [19–21]. Furthermore, events on one day may influence cortisol levels during the next, especially the CAR [21–23]. This can be a result of repeated stressors or negative emotions as a result of stressors that persist over a prolonged period of time [16]. A recent meta- analysis, P-curve analysis, and evaluation of the evidential value in existing studies concluded that worse psychosocial functioning was associated with CAR, among them general stress and work stress [24]. In a diary study on daily hassles, their appraisal as stressful was found to negatively predict the CAR [25]. Thus assuming that repeated stressors may lead to impaired psychosocial functioning, it reasonable to not only observe the immediate response of cortisol as outcome in EMA studies but also the response on a following day as an approach to the question why some predictors but not others are associated with some measures of CAR [24]. Cortisol in EMA studies can be reliably determined from saliva samples that subjects can obtain themselves [15].

The response of the ANS or autonomic stress reactivity typically refers to changes in HR and HRV or blood pressure due to an activation of the sympathetic nervous system and deactivation of the parasympathetic nervous system in response to acute stressors [26]. The association of environmental stress like work stress with altered HRV has been repeatedly shown [27] and stress interventions have been demonstrated to improve HRV [28]. Only more recently HRV is used in EMA studies [29, 30].[31] As the response of HRV can be immediate or delayed [26], continuous measurement by means of a wearable ECG for 24 h [29, 31, 32] or longer [30] is appropriate. Technical progress also allows for measuring HRV by pulse detection with a belt around the thorax or at the wrist [33, 34].

Ambulatory blood pressure (ABP) measurement is a mean to reflect effects of momentary psychological demands (e.g., stressful events) on the—short-term—blood pressure regulation which is mainly controlled by the ANS. This is useful in EMA studies to study the effect of stressful events [35, 36], or negative emotions [37]. ABP is usually measured by means of a cuff around the upper arm using an automated, auscultatory ABP monitor [38].

EDA is the common term for all electrical phenomena in skin [39]. EDA reflects only the sympathetic nervous system activity of the ANS [40]. It has been used in the laboratory for a long time but due to advances in technology may be used in real world environments [34, 41], making it an option for EMA studies [42]. Measurement is done e.g. by use of a wrist band. EDA can be computed by applying a small current and measure the resistance of skin between two placed electrodes [34].

Salivary alpha-amylase (sAA) reflects basal activity of the sympathetic nervous system [43, 44]. It is used to detect the ANS reaction to external psychosocial stressors [45, 46] and has therefore been used to measure stress responses and the effect of stress management interventions [47, 48].

Stress reactivity denotes activation of the ANS and HPA axis by acute psychosocial stress and the fact that following stress responses vary in magnitude between individuals but may also be influenced by contextual factors intra-invidually [49, 50]. Stress reactivity depends on several determinants such as gender, age or life style factors including physical activity or sleep [51–53]. Furthermore, dysregulation of stress response systems has been described in combination with chronic stress and illnesses such as depression, post-traumatic stress disorders (PTSD) and cardiovascular diseases [13, 54–56]. Such dysregulation might manifest in blunted (i.e. hypoactivation) and exaggerated (i.e. hyperactivation) stress responses. Chronic stress, for example, is associated with increased baseline functioning of the HPA axis, ANS and immune system and at the same time with blunted responsiveness of the HPA axis to recurrent stressors in the sense of habituation [4, 54, 55, 57]. Likewise, chronic stress is related to an altered HPA response and delayed recovery of the HPA system after occurrence of new stressors [54]. Dysfunctional -blunted as well as exaggerated- stress responses are associated with increased health risks including higher susceptibility to develop cardiovascular diseases [50]. Hence, it appears crucial to identify moderator variables being associated with altered physiological stress responses.

Acute stress responses and their moderators have been commonly examined in experimental research using specific stressors under laboratory conditions [10, 53]. This approach has important advantages including possibilities to standardize stress exposure and environmental variables. It is therefore highly suitable to examine responses to various stressor types and to investigate moderation of between-subject variables and variables that are easy to manipulate in the laboratory. In this sense, a previous meta-analysis found that cortisol responses to a combination of a public speaking and cognitive task were higher than to one of those tasks alone or to noise exposure or emotion induction. Furthermore, they found that social-evaluative threat and uncontrollability in combination with motivated performance are stressors characteristics that elicit strongest cortisol responses [10]. Regarding moderation variables, Allen et al. 2014 summarized research on the TSST and concluded that age, gender, genetic factors, appraisal of the laboratory task, social support and status moderate stress responses on the TSST [53]. However, laboratory research reaches limitations when it concerns possibilities to investigate how stress responses unfold during daily life routines and to investigate the impact of acute stressors that are difficult to recreate in the lab such as stressors in partnership [58].

In contrast, EMA studies examine stressors naturally occurring outside the laboratory. EMA allows for continuous or recurrent moment-to-moment measurement of physiological, behavioral and cognitive responses in real-life tracked over subsequent days or weeks. Methodological advances including newer generations of software to handle big physiological measurement data, multilevel analysis and further technological developments like mobile applications have made it much easier to collect and analyze complex EMA data than ever before [59, 60]. Various data collection methods may apply, including self-report measures, expert-based observations and the use of ambulatory physiologic monitoring devices [61]. EMA approaches are therefore highly suitable to examine within-subject associations between stressors occurring during daily life and subsequent stress responses. Furthermore, they provide opportunities to investigate moderation effects of variables that highly fluctuate during daily life and are hardly manipulable within laboratory conditions. Investigating moderator variables in daily life has been repeatedly called for in recent reviews and meta-analyses [15, 16, 24, 37]. If the literature retrieved allows indications of a specific context that influences stressor—stress response relationship [62], this will be presented in this review.

Over the last 20 years, the use of EMA in stress research is growing, making it necessary to summarize and map the accumulating evidence. Two recent meta-analyses examined EMA-studies investigating emotions and concurrent cortisol and blood pressure responses and found small positive within-subject associations between negative emotions and cortisol as well as blood pressure levels [16, 37]. Another meta-analysis has focussed on CAR and found that worse psychosocial functioning was associated with lower area under the curve (AUC) of the awakening repsonse. [24]. Other EMA studies have focused on subjectively measured stress responses, which have recently been summarized by a meta-analysis of diary studies about work stress during daily life [14]. Reviews about EMA studies investigating associations between acute psychosocial stressors or acute subjective stress and objectively measured physiological stress reactions are scarce. For instance, there are few exceptions with narrowed scope including a recent review targeting specific psychophysiological outcomes in psychiatric populations [63] and a review solely targeting cortisol reactions but missing a systematic literature search [15]. Furthermore, a very recent systematic review found mixed evidence regarding associations of perceived stress and cardiovascular reactions [64]. The authors of this study noted that blood pressure, heart rate and HRV were most often investigated and that perceived stress was operationalized in very different ways potentially explaining disparate findings [64]. It might therefore be useful to discuss those results by differentiating for outcome and operationalization of stress. Furthermore, a systematic overview on findings regarding potential moderators of associations between stress and physiological outcomes during daily life is still missing.

A comprehensive systematic review of EMA studies regarding acute pyschosocial stressors or subjective stress and objectively measured physiological stress reactions would thus be helpful to investigate which types of stressors typically occurring during daily life elicit physiological responses and whether there are certain inter- and intra-individual variables that might moderate those responses. This would provide indications to better understand the impact of stressors and to assess individual risk profiles for future stress-related diseases. This may serve as basis to develop tailored interventions considering the stressors that are experienced during daily life and specific intra- and inter-individual moderators. Furthermore, such overview will provide information on methodological aspects that are useful to consider within future EMA studies on stressor-strain relationships.

### Objectives

In view of the growing and heterogeneous field of EMA-assisted stress research, this systematic review aims to provide a comprehensive overview on EMA studies analyzing common organic or physiological reactions to acute stressors as well as acute subjective stress during daily life. This review will thereby differentiate between stressor type and outcome and will investigate variations among different life domains and populations. The comprehensive consideration of physiological stress responses was chosen because those considered may contribute to the allostatic load and thus to disease risk.

For this purpose, we postulate the following two research questions:

- Which physiological reactions to acute stressors and acute subjective stress have been observed during daily life by EMA studies?
- Which factors were found to intra- and inter-individually moderate physiological stress reactions during daily life by EMA studies?

In this review, we will include studies investigating physiological stress responses following either negative events or sensations being potentially able to provoke stress reactions (i.e. stressors, daily hassles) or the subjective feeling of being stressed (i.e. global subjective stress or momentary perceived distress). We also include subjective feelings of being stressed as exposure due the inconsistent definition, distinction and measurement of psychological stress and stressors in the EMA research literature [64]. This definition will exclude cognitive, emotional or behavioral responses to stress (e.g. mood, arousal, negative affect, stress coping) and all types of physical stressors (e.g. inducing pain).

## Materials and methods

We conducted a systematic review following the Preferred Reporting Items for Systematic Reviews and Meta-Analyses (PRISMA) guidelines [65]. This review was pre-registered at PROSPERO (PROSPERO 2020 CRD42020163178).

### Literature search and study selection

A systematic literature search was performed in electronic databases using *Web of Science* (all databases), *MEDLINE* (PubMed) via *Ovid* (Ovid MEDLINE(R) and *Epup Ahead of Print*, In-Process & Other Non-Indexed Citations and Daily) and *PsycINFO* via Ovid. The latest update of this search was performed on 29 November 2021. The search terms “ecological momentary assessment”, “diary”, “experience sampling”, “ambulatory monitoring”, “event-sampling” and “real-time assessment” were combined with “stress*”, “distress” and “strain”. Additional Medical Subject Headings (MeSH) were used for literature search in MEDLINE (Ecological Momentary Assessment; Monitoring Ambulatory; Stress, Psychological) and PsycINFO (Ecological Momentary Assessment; Occupational Stress; Psychological Stress). Since this search strategy provided more than 10,000 unscreened results, we further restricted our search by free text search terms and MeSH terms for physiological stress reactions (S1 Table). These search results were limited to studies published in English, German, Dutch and Portuguese due to authors’ fluencies. A search string example is given in S2 Table in the supporting information. Non-systematic literature searches were conducted using *Google Scholar* and *World Cat Database* to identify dissertations texts and other grey literature.

After duplicate removal, titles and abstracts were screened for eligibility based on the afore-mentioned criteria independently by two reviewers using the online platform *Rayyan* [66]. Remaining articles were read and assessed for eligibility. EMA and diary studies were included if acute stressors, daily negative events or subjective stress were measured as exposure and physiological stress responses were measured as outcome at least on a daily basis for at least three times in total. Further inclusion and exclusion criteria are listed in Table 1.

**Table 1:** Inclusion and exclusion criteria during study selection.

	Inclusion criteria	Exclusion criteria
**Population**	All (humans)	No restriction
**Exposure** ^1^	Acute stressors (e.g. while experiencing a novel ambiguous social situation) Daily negative events/hassles Global subjective stress Severity of acute stressors	Chronic stress Physical stress (e.g. noise, heat, cold) Artificial laboratory stress (e.g. Trier Social Stress Test) Single life events/single real-life stressors (e.g. natural disaster) Subjective and behavioral responses Mood, negative affect (e.g. anger, fear)
**Outcome**	Physiological stress reactions (e.g. blood pressure, heart rate variability, cortisol) measured by objective measurement methods	Subjective outcomes (e.g. measured using questionnaires, interviews) and/or behavioral outcomes (e.g. physical activity)
**Study design**	Ecological momentary assessment studies (EMA) Diary studies At least daily measurements of exposure and outcome Exposure is measured before or at the same time as the outcome	Non empirical studies Experimental or intervention studies Pharmacological clinical trials Case studies Studies with less than three measurement points Retrospective data collection Secondary analysis of published research

^1^ This classification has been developed for this review in accordance to stress typology as described in Epel et al. [8]

Twenty percent of articles were double screened and conflicts were resolved by two authors. As only few conflicts occurred (i.e. 12 out of 806 double screened articles), eligibility criteria were slightly refined in response and the remaining articles were independently screened by the same two authors. References of a random sample of included studies were screened for additional studies.

### Extraction of study characteristics

Study characteristics including study population, setting, inclusion and exclusion criteria, compliance, duration and frequency of measurement, maximum time lag between exposure and outcome, exposure and outcome variables, confounder, statistical analyses, and results on main and interaction effects were extracted from included studies on a standardized data extraction sheet using Excel by one author (JW) and were controlled by a second author (JA). Acute stressors and acute subjective stress was classified according to Table 2.

**Table 2:** Classification scheme of acute stressors and acute subjective stress as independent variable.

Classification	Definition	Example questionnaire items
Occurrence of acute stressors	Occurrence of any stressor within a given time period using a dichotomous yes/no scale.	Participants are asked on a dichotomous yes/no scale whether they experience a stressor, such as “Did anything happen that most people would consider stressful?”.
Number of acute stressors	Number of stressors within a given time period using a continuous scale.	Participants are asked on a dichotomous yes/no scale whether they experience a number of stressors, such as "Did you have any arguments or disagreement with anyone?” according to the Daily Inventory of Stressful Events [7]. Those items are summed up on a scale starting from 0 = “no stressors occurred”.
Occurrence of acute stressors within a life domain^1^	Occurrence of any stressor within a given time period and within a specific life domain (e.g. at work, at home, during social interactions) using a dichotomous yes/no scale.	Occurrence of acute stressors at work: “Has an event occurred at work that most people would consider stressful?” on a dichotomous yes/no scale.
Severity of acute stressors	Magnitude of one or more stressors using a continuous scale.	“How stressful was the most important event” on a scale from 0 =“not at all” to 10 =“very much”.
Severity of acute stressors within a life domain^1^	Magnitude of one or more stressors within a specific life domain using a continuous scale.	Severity of acute stressors regarding work: “Were you required working hard” on a scale from 0 =“not at all” to 4 =“very much” according to the Diary of Ambulatory Behavioral States [67].
Global subjective stress^1^	General assessment of feeling stressed using a continuous scale (e.g. perceived stress).	“How stressed do you feel” on a scale from 0 =“not at all” to 10 =“very much”.

^1^ This classification has been developed for this review in accordance to stress typology as described in Epel et al. [8]

### Quality assessment

Reporting guidelines for EMA studies [68] and previous quality assessment tools [69, 70] were adapted to develop a pragmatic quality assessment tool for EMA studies in stress research. This quality assessment included following characteristics:

#### Frequency of measurement

- Have exposure (i.e. acute stress) and outcome (i.e. physiological stress reactions) been measured for at least three times per day?

#### Compliance

- Was a compliance rate (i.e. number of answered prompts per total prompts) of at least 80% reported for the exposure?

#### Recall bias

- Does the data collection method allow backfilling (e.g. paper- and pencil questionnaires)?
- Do stress ratings include time frames of more than 30 minutes?
- If both questions are yes: strong; if one question is yes: moderate; if both questions are no: low

#### Confounding

- Have appropriate statistical analyses been applied to control for hierarchical structure of data (e.g. multilevel analysis)?
- Were other key potential variables [67, 71, 72] measured and adjusted for their impact on the relationship between exposure and outcome?
  - Gender; age; ethnicity; body mass index (BMI); recent consumption of food, alcohol, caffeine or nicotine; medication; current physical activity; mental disorders
  - Specific for cortisol: daytime; waking time; use of hormonal contraceptives
  - Specific for blood pressure, HR and HRV: posture; talking; cardiovascular disorders
  - Specific for electrodermal activity (EDA): talking; temperature
  - These variables could have been statistically controlled for or could have been taken into account as eligibility criteria. Participants could have also been asked to avoid certain behavior patterns (e.g. consumption of food, alcohol, tobacco; [15]).
- If both questions are yes: low; if one question is yes: moderate; if both questions are no: strong

#### Reliability and validity of exposure measure

- Were stress ratings reliably measured (e.g. analyzed by Generalizability Theory Analysis [73])?
- Were stress ratings valid (e.g. construct validity analyzed by multilevel confirmatory factor analysis, concurrent validity [74])?
- If both questions are no: low; if one question is yes: moderate; if both questions are yes: high

We did not assess the quality of outcome variables, because they were defined as being measured with objective measurement procedures (see, inclusion criteria in Table 1). Furthermore, the vast range of possible outcome variables might have complicated standardized and reliable assessment of outcome validity.

Quality assessment was independently performed by two authors (JW, JA) and conflicts were resolved by consensus.

## Results

### Literature search

**Fig 1:**
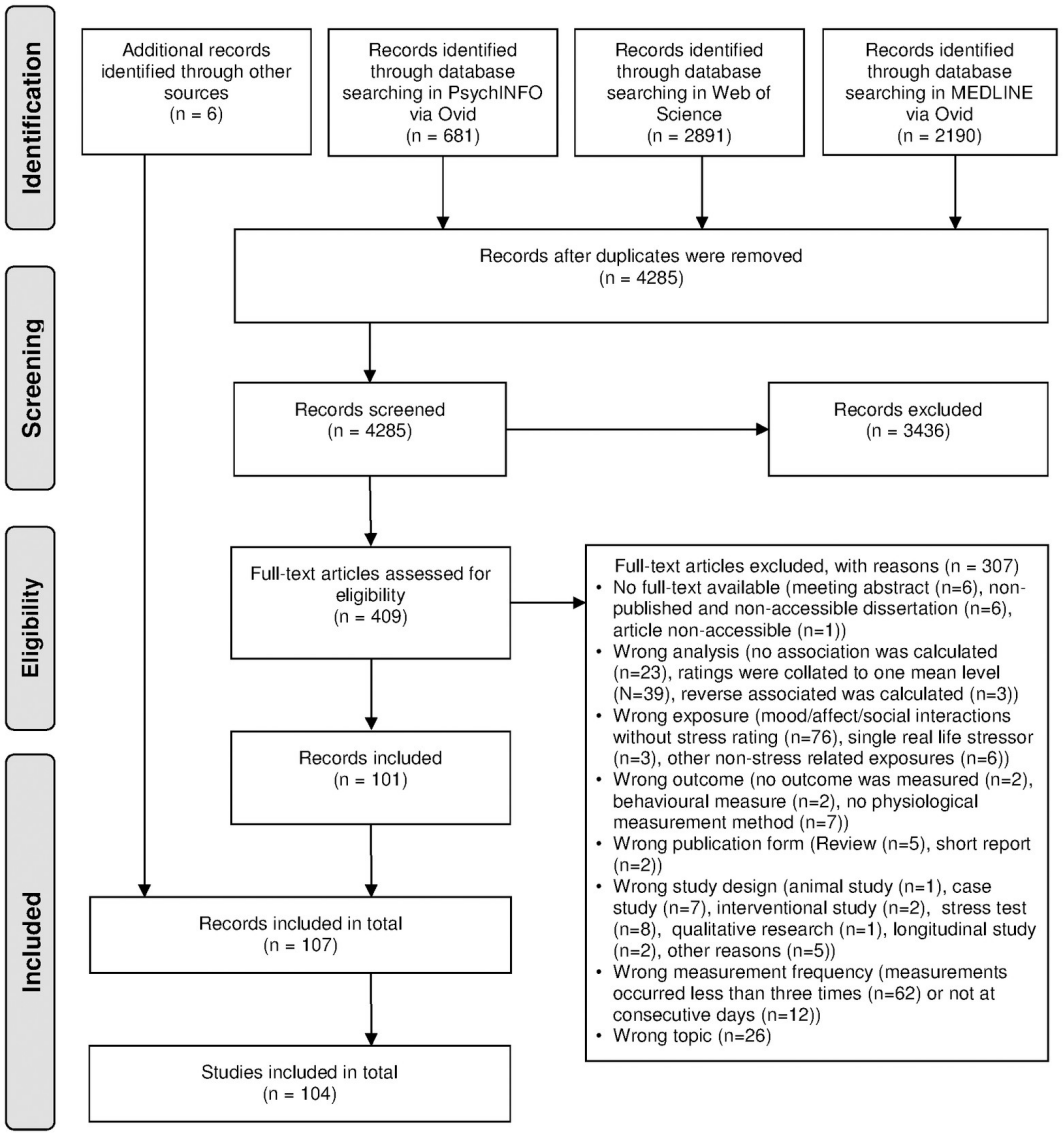
Flow chart of literature search (adapted from PRISMA guidelines [65]).

Fig 1 shows the flow diagram of the literature search. The literature search in PsycINFO, Web of Science and MEDLINE resulted in 4285 records after duplicates were removed. After title, abstract and full-text screening, 101 records remained. Furthermore, six records were identified through non-systematic literature search in Google Scholar, World Cat Database and reference screening of included studies. Finally, 107 eligible publications comprising 104 unique studies were included in this review.

Studies were increasingly published in recent years, with 77 of 104 studies being published between 2010 and 2021, 21 studies between 2000 and 2009 and six studies before 2000. Most studies were conducted in the United States (65%, n = 68/104), 31 studies in Europe, three studies in Canada and two studies in Singapore. Salivary cortisol levels (58%, 60/104) and cardiovascular outcomes (32%, 33/104) were most frequently studied, followed by salivary alpha-amylase (sAA); 5%, 5/104) and other physiological stress reactions (8%, 8/104). In supplementary material, a tabular description of each study including information on study population, setting, exposure variables, sampling schedule and results (S3–S5 Tables) and an overview on the quality assessment (S6 Table) is given.

### Main findings per outcome

#### Current salivary cortisol levels

*Study characteristics*. Sample sizes of the 25 studies ranged from 30 to 566 participants per study (Median = 115). Various age groups and populations were studied including employees of different industries, students and specific clinical groups (e.g. mental disorders, fibromyalgia, spinal cord injury). Most studies included more female than male participants (68%, 17/25, Median = 61% female participants). Sampling schedules ranged from two to fourteen sampling days per participant and study (Median = 4 days). Those studies either measured occurrence of acute stressors for the last 30, 60, 90, 120 or 145 minutes before cortisol sampling. Severity of acute stressors and global subjective stress was measured concurrently with cortisol sampling in seven cases or for periods of 20 minutes up to 5.5 hours preceding cortisol sampling.

*Quality assessment*. Nearly all studies measured exposure and outcome for at least three times per day (96%, 24/25). Compliance was good (≥ 80%) in 17 and not reported in three studies. Eight studies were rated as having a low risk, eleven as having a moderate risk and six as having a strong risk of recall bias. Nearly all studies were rated to have moderate levels of confounding (96%, 24/25). Fifteen studies used at least partly valid or reliable exposure measures, whereas nine studies did not report reliability and validity of their exposure measures.

*Results*. Severity of acute stressors [75–79] was not directly associated with current cortisol levels. However, three studies observed moderating variables. Having a sibling with psychotic disorder [75] or reporting high concurrent engagement coping [78] was related to increased stress reactivity. Spinal cord injury [79], high coping efficacy and engagement coping in general [78] were related to decreased stress reactivity. Whereas one study examining pregnant women [80] and one study examining working adults [81] found positive relationships between global subjective stress and current cortisol levels, other studies with working adults [82] and women with fibromyalgia [83] found no such association. Furthermore, one study among healthy adults suggests that several subjective responses to stress attenuate a positive effect of global subjective stress on momentary cortisol levels [42]. Positive relationships between cortisol and acute stressor severity during social interactions among school children [84] and women [76] were found. Within those studies, stressor severity was measured for periods up to 20 minutes preceding cortisol sampling. Another study on a healthy study population measuring acute stressor severity during social interactions and cortisol concurrently found no association [85]. Other studies using longer time lags between acute stressor severity during social interactions [82, 86] or using a study population with autism spectrum disorders [87] also found no association. Regarding severity of acute stressors at work, cortisol levels were positively associated with concurrent measurement of the ability to meet work demands [88] and with performance pressure [89] but not with task failure measured for periods up to one hour preceding cortisol sampling. Furthermore, trait anxiety was identified as a moderator as performance pressure was only positively associated with cortisol levels among study participants having high anxiety levels [89]. Other studies using longer time lags between severity of acute stressors at work and cortisol levels found no association [90, 91]. Regarding severity of acute stressors in current activity, positive associations were found with concurrent cortisol levels among healthy study populations [76, 92]. Among study populations with small sample sizes and specific health conditions including spinal cord injury [79], autism spectrum disorders [87] and 22q11.2 deletion syndrome [92] no such association was found. Finally, four out of six studies identified the occurrence of acute stressors to be positively related to cortisol levels among healthy study populations [77, 93–97]. Previous number of bipolar events and major depressive disorders were found to decrease cortisol reactivity [96, 98].

#### Cortisol awakening response, diurnal cortisol slope and single day-time levels of cortisol

Since most studies examining the diurnal cortisol slope also examined single-day time levels of cortisol and next-day CAR, study characteristics and quality assessment of those studies will be described together.

*Study characteristics*. Thirteen studies examined next-day CAR (i.e. slope of cortisol levels between waking-up and 30 minutes after waking-up), 17 studies examined diurnal cortisol slope (i.e. decline of free cortisol levels during the day) and eight studies examined evening cortisol levels. Sample sizes ranged from 28 to 1736 participants per study (Median = 126). Those samples included children as well as adult study populations, parents of children with chronic health conditions and other family caregivers, employees, retirees, students and specific clinical groups (e.g. cancer, premutation of fragile X). Most studies included more female than male participants (79%, 23/29, Median = 57% female participants). Sampling schedules ranged from three to eight sampling days per participant and study (Median = 4 days). Most studies measured number (52%, 15/29) or occurrence (34%, 10/29) of acute stressors in general of within life domains. There were also some studies measuring severity of acute stressors in general (10%, 3/29) and within life domains (17%, 5/29) as well as global subjective stress (10%, 3/29). Diurnal cortisol slope and bedtime levels of cortisol were related to stressors or stress at the same day, whereas the CAR was related to previous-day stressors or stress.

*Quality assessment*. Except for two studies, all studies were daily diary studies with only one measurement of exposure but several measurements of salivary cortisol per day. Compliance was good in 15 (52%, 15/29) and not reported in 14 studies (48%, 14/29). Most studies were assessed as having a moderate risk of recall bias (90%, 26/29). Risk of confounding was rated as moderate in 23 (79%, 23/29) and low in six studies (21%, 6/29). Fifteen studies (52%, 15/29) used at least partly valid or reliable exposure measures, whereas eleven studies did not report reliability and validity of their exposure measures (38%, 11/29).

*Results regarding next-day cortisol awakening response*. Number of acute stressors regarding discrimination among black American students was positively associated with the CAR [99]. In addition, a positive association regarding occurrence of acute stressors regarding violent crime in neighborhood among adolescents was found [100]. Those associations were not found for single measurements of cortisol levels at waking-up [99, 100]. However, Bai et al. 2017 found that among children acute stressors regarding social interactions and education were positively associated with single measurements of cortisol levels at waking up [101]. All other studies found no direct association between CAR, single measurements of cortisol levels at waking-up and number of acute stressors [25, 102–106] in general and regarding caregiving of children with fragile X syndrome [102]. There were also no associations found regarding number [107] and occurrence [108–110] of acute stressors due to social interactions and severity of acute stressors [25, 104, 111]. However, chronic caregiving stress was suggested to moderate those relationships [112–114]. Whereas parents without chronic caregiving stress experienced increased cortisol awakening response after days with acute stressors, this association was attenuated in parents experiencing chronic caregiving stress [112, 113]. Another study observed positive associations between levels of cortisol at waking-up and occurrence of acute stressors at work among mothers with, but not among mothers without chronic caregiving stress [114]. Other studies found cortisol at waking-up being only positively associated with severity of acute stressors in case rumination was high on the prior day [111] or with number of acute stressors if no negative life events were previously reported [104].

*Results regarding bedtime cortisol levels*. Among black American students, number of acute stressors regarding discrimination was found to be positively associated with bedtime cortisol levels [99]. Also severity of acute stressors regarding social interactions was positively associated with bedtime cortisol levels in men but not women in a sample of two-earner middle class families. However, severity of acute stressors at work was negatively associated with bedtime cortisol levels in this same study sample [115]. All other studies that examined bedtime cortisol levels found no association neither for number [103] and occurrence of acute stressors [116] in general, occurrence of acute stressors regarding violent crime in neighborhood [100], severity of acute stressors regarding family life [117] and work-family conflict [118]. Those relationships were examined in samples of children, adolescents as well as adult study populations.

*Results regarding diurnal cortisol slope*. Studies examining acute stressors related to interactions among family members found significant associations with diurnal cortisol slope. Negative interactions with adult children and children with physical-emotional problems were associated with steeper diurnal cortisol slope at the same day [108, 109]. No such association was found for negative interactions with parents [109]. In contrast, Barker et al. 2012 found that severity of acute stressors but not number of acute stressor were associated with a flattened diurnal cortisol slope among parents with children with serious mental disorders [112]. In addition, a flatter cortisol slope was associated with number of acute stressors in marriage [119]. Furthermore occurrence of acute stressors related to behavioral problems of the spouse with mild cognitive impairments was related to elevated cortisol levels and a flattened diurnal slope [120, 121]. However, another study examining number of acute stressors related to the care for someone with dementia among family caregivers was not associated with diurnal cortisol slope [122, 123]. Studies examining other types of acute stressors found no association with diurnal cortisol slope (i.e. number of acute stressors [124] number of acute stressors regarding education [101], social interactions in general [101, 107] and discrimination [99], occurrence of acute stressors in general [116, 125] and regarding social interactions [110, 125], severity of acute stressors [111], global subjective stress [124, 126, 127].

#### Area under the curve (AUC) of salivary cortisol levels per day

*Study characteristics*. Sample sizes of the ten studies ranged from 83 to 1694 participants per study (Median = 147). Those samples included students, pregnant women, general adult populations, health care professionals and cancer survivors. Most studies included more female than male participants (80%, 8/10, Median = 63% female participants). Sampling schedules ranged from three to twelve sampling days per participant and study (Median = 4 days). Most studies measured number (50%, 5/10) or occurrence (40%, 4/10) of acute stressors in general or within life domains. Within all studies, AUC of cortisol levels were related to same-day stress levels.

*Quality assessment*. Except for two studies, all studies were daily diary studies with only one measurement of exposure but several measurements of salivary cortisol per day. Compliance was good in four and not reported in six studies. Most studies were assessed as having a moderate risk of recall bias (70%, 7/10). All studies were rated as having a moderate risk of confounding. Six studies used at least partly valid or reliable exposure measures, whereas three studies did not report reliability and validity of their exposure measures.

*Results*. One study found that number of acute stressors was positively associated with AUC of cortisol [128]. However, this relationship was attenuated at days when study participants performed volunteer work [128]. Among study populations of students and older adults with smaller sample size no relationship between number of acute stressors and AUC of cortisol was found [129, 130]. However, among Chinese American school children the number of acute stressors regarding education and among black American students the number of acute stressors regarding discrimination were also positively associated with AUC of cortisol [131]. Occurrence of acute stressors in general and regarding social interactions was positively associated with AUC of cortisol among healthy individuals but not among cancer survivors [116, 125]. Whereas a positive association between occurrence of acute stressors at work and AUC of cortisol was found among a sample of adults providing support to their parents [132], a study among health care professionals with smaller sample size found no such association [133]. Among pregnant women, global subjective stress was positively associated with AUC of cortisol [80].

Table 3 represents an overview on study results regarding moderating variables on within-subject associations between acute stressors or stress and cortisol.

**Table 3:** Moderating variables on within-subject associations between acute stressors or stress and cortisol.

	Current cortisol levels				Awakening response of cortisol^1^				Diurnal cortisol slope^2^				AUC cortisol				Single day-time levels of cortisol (e.g. at wake up, in evening)			
	/	↑	↓	Ref.	/	↑	↓	Ref.	/	↑	↓	Ref.	/	↑	↓	Ref.	/	↑	↓	Ref.
***Sociodemographic variables (BS)***																				
• Age	✓	-	-	[93]	✓	-	-	[107]	✓	-	-	[107]					✓	-	-	[106]
• Gender (female vs. male)	✓	✓	✓	[87, 93, 96]					-	-	-						✓	-	-	[103, 117]
• Socioeconomic status	✓	-	-	[88]																
***Health-related variables (BS)***																				
• Mental disorders	✓	-	✓	[87, 98, 134]					✓	-	-	[124]								
• Having a sibling with psychotic disorders	-	✓	-	[75]																
• Spinal cord injury	-	-	✓	[79]																
• 22q11.2 deletion syndrome	-	-	✓	[92]																
• Higher activation of the normal X chromosome when having a premutation of FMR1																	✓	✓	-	[135]
***Personality-related variables (BS)***																				
• Coping	-	-	✓	[78]																
• Trait anxiety	-	✓	-	[89]																
***Family-related variables (BS)***																				
• Chronic caregiving stress					-	-	✓	[112, 113]	-	✓	-	[112]	-	-	-		-	✓	-	[114]
• Parental warmth									-	-	-		-	-	✓	[129]	✓	-	-	[103]
• Marital satisfaction													-	-	-		-	✓	✓	[115]
• Family conflict and lack of parental affection during childhood													✓	-	-	[129]				
***Family-related variables (WS)***																				
• Providing support to parents													✓	-	-	[132]				
***Subjective responses to stress (WS)***																				
• Self-oriented thoughts	✓	-	-	[97]																
• Past-oriented thoughts	-	-	✓	[97]																
• Positive thoughts	-	-	✓	[97]																
• Negative affect	-	-	✓	[97]																
• Arousal	✓	-	-	[97]																
• Coping	✓	✓	-	[78, 97]																
• Rumination					✓	-	-	[111]	-	-	✓	[111]					-	✓	-	[111]
***Other variables (BS)***																				
• Life satisfaction	✓	-	-	[95]																
• Previous negative life events																	-	-	✓	[104]
• Satisfaction with network support									-	-	✓	[119]								
• Believing in incremental theory of intelligence	-	✓	-	[86]																
• Ethnic-racial identity					-	-	✓	[99]	✓	-	-	[99]	✓	✓	-	[99, 131]	-	✓	-	[99]
***Other variables (WS)***																				
• Time of the day	✓	-	-	[93]																
• Performing volunteer work													-	-	✓	[128]				
• Social contacts	✓	-	✓	[82]																

BS = between-subject moderating variables; Ref. = References; WS = within-subject moderating variables; / = no moderation effect; ↑ = higher levels of moderating variable increased relationship between acute stress and cortisol (i.e. increased stress reactivity); ↓ = higher levels of moderating variable decreased relationship between acute stress and cortisol (i.e. decreased stress reactivity);

^1^ ↑ = higher levels of moderating variable was associated with increased cortisol awakening response, ↓ = higher levels of moderating variable was associated with decreased cortisol awakening response;

^2^ ↑ = higher levels of moderating variable was associated with a flatter diurnal cortisol slope, ↓ = higher levels of moderating variable was associated with steeper diurnal cortisol slope.

#### Blood pressure

*Study characteristics*. Nineteen studies examined blood pressure. Sample sizes ranged from 33 to 477 participants per study (Median = 99). Various age groups and populations were studied, including employees of different industries, students and specific clinical groups (e.g. mental disorders, cardiovascular disease). Female and male participants were included in equal proportions (Median = 53% female participants). Sampling schedules ranged from one to ten days per participant and study, with eight studies using one sampling day. Exposure and outcome were measured at least every hour in 53% and at least every two hours in 79% of studies. Most studies measured severity of acute stressors within life domains concurrently (3 studies) or preceding the last 10 minutes (5 studies) of blood pressure measurement. Global subjective stress concurrently during (2 studies) or for periods of one hour preceding (1 study) blood pressure measurement was also examined. Occurrence of acute stressors in general and within life domains was examined by six studies with various time lags of up to six hours between exposure and outcome.

*Quality assessment*. Exposure and outcome were measured at least three times per day in most of the 33 studies (89%, 17/19). Compliance was rated as being good (i.e. ≥ 80%) in nine (47%, 9/19) and not reported in seven studies (37%, 7/19). Eight studies were rated to have low (42%, 8/19) and moderate (42%, 8/19) risk of recall bias respectively. Most studies had a moderate risk of confounding (63%, 12/19). Good validity and reliability of exposure measures was given in five studies (26%, 5/19), whereas eight studies have neither used valid nor reliable exposure measures (42%, 8/19).

*Results*. Among a sample of undergraduate students, the occurrence of acute stressors during periods of up to 30 minutes preceding blood pressure measuring was positively associated with systolic blood pressure (SBP) and diastolic blood pressure (DBP; [136]. Conversely, another study only found a positive relationship with DPB but not SBP among older compared to middle-aged adults [137]. Occurrence of acute stressors at work within the last ten minutes was positively associated with SBP and DBP, whereas occurrence of acute stressors during social interactions was only positively associated with SBP but not DBP [138]. However, a moderation effect of physical activity was found suggesting that occurrence of acute stressors at work and social interactions are only positively associated with SBP and DBP among individuals with low physical activity [138]. Also occurrence of acute stressors in academic education within the last hour was found to be only positively associated with SBP but not DBP and especially so for individuals with high test anxiety [139]. Occurrence of acute stressors regarding work-family conflict during preceding hours was not directly associated with SBP or DBP [111, 112]. However, family-supportive supervision seemed to moderate this relationship: family-to-work conflict was only associated with increased blood pressure if family-supportive supervision was low [111]. Mixed results were obtained regarding an association between severity of acute stressors at work within the last 10 minutes and blood pressure with two out of five studies suggesting positive relationships [35, 140–143]. Furthermore, job control was shown to attenuate this relationship [142]. Severity of acute stressors during social interactions was found to be positively related with blood pressure by four studies [35, 144–146]. Another study found no relationship [143]. Global subjective stress was associated with SBP and DBP in two studies [147, 148]. One further study showed that global subjective stress was associated with increased blood pressure among Vietnam veterans with PTSD but not among Vietnam veterans without PTSD [149].

#### Heart rate

*Study characteristics*. Seventeen studies examined relationships between HR and acute stressors or stress. Sample size ranged between 6 and 219 study participants per study (Median = 92). Female participants were slightly overrepresented (Median = 56% female participants). Sampling frequency ranged from one to ten days per participant (Median = 2). Exposure was measured at least once per hour by 42% and at least every two hours by 88% of studies. Most studies measured severity of acute stressors within life domains (7 studies) or global subjective stress (6 studies) for periods of up to 12 minutes preceding HR measurement. One study measuring global subjective stress used longer time frames as well as four studies measuring occurrence of acute stressors in general or within life domains.

*Quality assessment*. Except for two studies, all other studies reported to measure exposure and outcome for at least three times per day. Ten studies reported good compliance (59%, 10/17). Most studies were rated as having a moderate (76%, 13/17) risk of recall bias and confounding (71%, 12/17). Ten studies did not report on validity and reliability of exposure measures (59%, 10/17), whereas five studies used at least valid or reliable tools to measure exposure (29%, 5/17).

*Results*. Occurrence of acute stressors in education was not associated with HR [139]. Whereas occurrence of acute stressors regarding work-to-family conflict during preceding hours was not associated with HR, opposed results were obtained regarding family-to-work conflict with one study finding a positive [150] and one study finding a negative relationship [151]. Positive relationships with severity of acute stressors in general [152] and at work [143, 153] were identified. Another study found no direct association between severity of acute stressors at work and HR, but an interaction between job demands and control showed that HR was especially high in situations of high demands and low control [141]. No interaction between job demands and control however was found by another similar study [153]. All studies examining severity of acute stressors regarding social interactions found no relationship with HR [143, 144, 146, 152]. Positive associations between global subjective stress and HR among healthy adults [148, 152, 154, 155] were identified. Furthermore, chronic stress was found to moderate this relationship such that only among individuals with chronic stress such association was found [154]. Whereas the results of one study suggested global subjective stress being only (positively) related to HR in individuals with a diagnosis of PTSD [156], another study found no moderation effect of PTSD [149]. Among adults being overweight, global subjective stress did not seem to have an effect on HR [157].

#### Heart rate variability

*Study characteristics*. Eight studies examined relationships with HRV. Sample sizes ranged between 43 and 219 study participants with a median of 50% female participants. Those studies included students, teachers, police workers, firefighters and individuals with PTSD. Two studies included general adult study populations. Sampling duration ranged from one to five days (Median = 2 days) per participant with at least six measurements per day. Those studies measured global subjective stress for periods of less than 30 minutes preceding HRV measurement (3 studies), whereas most studies measuring occurrence of acute stressors (2/3 studies) and severity of acute stressors at work (2 studies) used longer periods of up to one or two hours preceding HRV measurement.

*Quality assessment*. Except for one study, which did not report on exposure measurement frequency, all other studies measured exposure and outcome for more than three times per day. Compliance was good in three studies and not reported by four studies. The risk of recall bias was moderate in six and low in two studies. All studies were rated to have a moderate risk of confounding. No study reported on validity and reliability of their exposure measures.

*Results*. No direct relationships were found between occurrence of acute stressors and RMSSD or SDNN [158–160]. However, study participants of two of those studies were on average below 30 years of age [158, 160, 161] and the third study identified age as a moderator [159]. This study suggested that stressors affecting several life domains are negatively and stronger associated with RMSSD among individuals of higher age but that this moderation was reversed for stressors affecting only one life domain [159]. Whereas one study demonstrated a negative association between severity of acute stressors at work and RMSSD and SDNN among firefighters [162], another one among school teachers did not find such association [163]. Negative associations between global subjective stress and high-frequency domain of HRV and RMSSD were found by one study among healthy adults [155]. Furthermore, better cardiorespiratory fitness was shown to attenuate this relationship [164]. However, a third study only found negative associations between global subjective stress and high- and low-frequency domains of HRV among individuals with but not without PTSD [156].

Table 4 represents an overview on study results regarding moderating variables on within-subject associations between acute stress and cardiovascular outcomes.

**Table 4:** Moderating variables on within-subject associations between acute stress and cardiovascular outcomes.

	Systolic blood pressure				Diastolic blood pressure				Heart rate				Heart rate variability			
	/	↑	↓	Ref.	/	↑	↓	Ref.	/	↑	↓	Ref.	/	↑	↓	Ref.
***Sociodemographic variables (BS)***																
• Age					-	✓	-	[137]					-	✓	✓	[159]
• Gender (female vs. male)	✓	-	-	[143]	✓	-	-	[143]	✓	-	-	[143, 155]	✓	-	-	[155]
• Ethnicity	✓	-	-	[143]	✓	-	-	[143]	✓	-	-	[143]	-	-	-	
***Health-related variables (BS)***																
• Post-traumatic stress disorder	-	✓	-	[149]	-	✓	-	[149]	✓	✓	-	[149, 156]	-	✓	-	[156]
• Cardiorespiratory fitness													-	-	✓	[164]
***Personality-related variables (BS)***																
• Anxious attachment	✓	-	-	[146]	✓	-	-	[146]	✓	-	-	[146]				
• Avoidant attachment	✓	-	-	[146]	-	✓	-	[146]	✓	-	-	[146]				
• Test anxiety	-	✓	-	[139]	✓	-	-	[139]	✓	-	-	[139]				
• Hostility	✓	-	-	[144]	✓	✓	-	[144, 165]	✓	-	-	[144]				
• Rumination													✓	-	-	[160]
• Neuroticism													✓	-	-	[160]
***Family-related variables (BS)***																
• Experience of childhood parental death and parental caring	✓	-	-	[136]	✓	-	-	[136]								
***Work-related variables (BS)***																
• Work-supportive family	✓	-	-	[150]	✓	-	-	[150]	✓	-	-	[150]				
• Family-supportive supervision	-	-	✓	[150]	-	-	✓	[150]	✓	-	-	[150]				
• Job resources	-	-	✓	[142]									✓	-	-	[163]
• Chronic work stress									-	✓	-	[154]				
***Work-related variables (WS)***																
• Job resources	✓	-	-	[141]	✓	-	-	[141]	✓	✓	✓	[141, 143, 153]				
***Other variables (BS)***																
• Physical activity	-	-	✓	[138]	-	-	✓	[138]								
• Social support	-	-	✓	[147]	-	-	✓	[147]								
***Other variables (WS)***																
• Physical activity	-	-	✓	[138]	-	-	✓	[138]								
• Resilience													-	✓	-	[162]
• Being in company with someone													✓	-	-	[160]
• menstrual cycle phase (luteal vs. follicular phase)									-	✓	-	[155]	-	✓	-	[155]

BS = between-subject moderating variables; Ref. = References; WS = within-subject moderating variables; / = no moderation effect; ↑ = moderating variable increased relationship between acute stress and cardiovascular outcomes (i.e. increased stress reactivity); ↓ = moderating variable decreased relationship between acute stress and cardiovascular outcomes (i.e. decreased stress reactivity)

#### Salivary alpha-amylase

*Study characteristics*. Three daily diary studies with four to eight sampling days per participant [120, 123, 166] used middle-aged to older study populations with two of those studies examining caregivers with a high percentage of female participants (88% and 90%, respectively). Two other studies examined a sample of university students with one sampling day and a frequency of 15 measurements per participant (58% female participants [167]) and a sample of working adults with four sampling days and six measurements per participant and day (33% female participants [82]), respectively.

*Quality assessment*. Three out of five studies used daily diary designs with one measurement of exposure per day to examine within-subject associations with sAA levels [120, 123, 166]. Those three studies had good compliance rates and moderate risk of confounding [120, 123, 166]. Two studies measured outcome and exposure for more than three times per day [82, 167]. Risk of confounding was low. All five studies had a moderate risk of recall bias and validity and reliability of exposure was low to moderate.

*Results*. One study found a positive relationship between the diurnal slope of sAA and occurrence of acute stressors regarding marriage [120]. Number of acute stressors during social interactions was not associated with overall levels per day, diurnal slope and next-day awakening response of sAA [166]. Depending on daytime and patient’s type of memory- and behavior-related problems, mixed results regarding associations between diurnal slope of sAA levels and number of acute stressors related to caregiving were obtained [120, 123]. No association between global subjective stress, severity of acute stressors regarding social interactions and current sAA levels was found [82, 167].

#### Other outcomes

Within-subject associations between acute stress and urinary levels of adrenaline, temporalis muscle activity, electromyographic (EMG) activity and salivary levels of dehydroepiandrosterone sulfate (DHEA-S) were analyzed by one study respectively (i.e. four studies in total). Urinary levels of adrenaline were measured by a three day daily diary design in a sample of 104 participants [90]. In this study, a positive association with severity of acute stressors at work was found among men but not women. No direct associations were identified between global subjective stress and temporalis muscle [168], EMG activity [169] or salivary levels of DHEA-S [107]. However, a significant moderation effect of age on the association of number of acute stressors regarding social interactions and DHEA-S levels occurred [107]. Two EMA studies examined whether acute stress is associated with EDA. One study found a positive association between occurrence of acute stressors regarding discrimination and concurrent EDA within a sample of college students of racial and ethnic minorities if high traits of anger, anxiety, depression, loneliness or stress were reported [170]. The other study found that among female participants severity of acute stressors during social interactions with dating partners was positively associated with concurrent EDA if they had experienced aggression during childhood or partnership [171]. Finally, two daily diary studies examined blood glucose levels in patients with diabetes type I. Those studies found higher blood glucose levels being related to severity and number of acute stressors regarding diabetes but not to acute stressors in general [172, 173].

## Discussion

This systematic review summarizes the evidence base of 104 EMA studies regarding associations between acute stressors or subjective stress and physiological stress reactions during daily life. Cortisol responses, blood pressure and HR were most frequently studied. However, evidence regarding direct associations to acute stressors or stress was mixed. Other types of physical stress reactions were seldom investigated.

More than one third of included studies examining concurrent responses of salivary cortisol levels to acute stressors or subjective stress found direct positive relationships. Especially laboratory stress research has reliably initiated salivary cortisol reactions in response to acute social-evaluative stressors, such as speech and arithmetic tasks during the Trier Social Stress Test (TSST) [10, 174, 175]. This systematic review suggests that cortisol responses to acute psychological stressors or stress might be more difficult to detect in daily life compared to laboratory settings using the TSST. However, during the TSST a standardized stressor is used, whereas EMA studies measured subjective stress in daily life. One should therefore consider that even under laboratory conditions subjective stress and cortisol responses are not fully matched [176]. Especially among studies examining stressor severity within life domains among healthy individuals it is noteworthy that increased stressor severity was associated with higher levels of salivary cortisol when maximum time lags between those variables did not exceed 20 minutes [76, 84, 88, 92] and that studies allowing for longer time lags found no such association [82, 86, 90, 91]. This could be explained by the fact that cortisol levels peak between 20–40 minutes after stress exposure and decline afterwards [10]. Furthermore, it might on the first look be puzzling that stressor severity within life domains but not stressor severity in general was associated with cortisol levels. Those studies measuring stressor severity in general were asking participants to rate unpleasantness or stressfulness of the most important event within periods of one hour or more before cortisol sampling, whereas studies examining stressor severity within life domains asked to rate overall stressfulness of their work, activity or social interactions for a given period of time. Null findings among studies examining stressor severity in general might therefore be explained by neglecting that also other more positive events might have occurred and by the fact that they asked for only one specific and potentially completed event. Evidence that differentiation between ongoing and completed stressors within EMA studies is essential when examining their effects with salivary cortisol levels comes from studies measuring occurrence of any acute stressor [77, 93, 94]. Last but not least, health status seems to moderate stress reactivity since no or weakened associations between occurrence or severity of acute stressors and salivary cortisol levels were found for various clinical groups including spinal cord injury [79], fibromyalgia [83], 22q11.2 deletion syndrome [92] and remitted bipolar [98] or depressive disorders [96]. In accordance with results of laboratory studies among similar clinical groups, those findings corroborate evidence of HPA-axis dysregulation within pain- and stress-related disorders [177–180].

Aggregate measures of cortisol levels throughout the day including the awakening response and diurnal slope are mainly discussed to reflect biological markers for chronic stress and are related to subsequent health outcomes [19, 20, 24, 181], but a significant number of studies also observed day-to-day variation suggesting that they might depend on daily experiences including acute stress as well [21–23, 101]. Among EMA studies associations with the awakening response and diurnal slope of salivary cortisol were seldom found suggesting that they are not per se suitable to assess acute stress reactivity. However, there were few exceptions. First, studies assessing associations between different forms of violence and CAR among adolescents and young adults found positive associations [99, 100]. Second, also among school children, acute stressors regarding social education and education were positively associated with cortisol levels at waking-up [101]. Third, evidence arose that chronic caregiving stress moderates relationships. Associations between acute stressors and CAR were attenuated under chronic caregiving stress [112–114]. Furthermore, acute stressors were related to flattened diurnal slope among individuals experiencing chronic caregiving stress [112, 120, 121]. Under chronic overactivity of the HPA-axis, increased allostatic load might lead to a dysregulation of the HPA-axis [4], which is supported by previous research indicating that chronic stress results in lower cortisol levels and flatter diurnal slope [19]. This suggests that future studies should control for chronic stress when examining acute stress reactivity. Furthermore, other methodological limitations might have contributed to difficulties in detecting stress reactivity in studies investigating CAR and diurnal slope. Those studies used single evening ratings for daytime stress and thus large time lags between the occurrence of acute stress and cortisol sampling might have occurred. In addition, single evening ratings increase the risk of recall bias and small sample sizes and low number of sampling days reduces statistical power. Last but not least, CAR is discussed to reflect anticipatory reactions to stress in the upcoming day [182, 183]. This review, however, focused on reactions to concurrent or previous acute stress and therefore excluded studies examining associations between cortisol and prospective levels of stress (e.g. anticipated stressful events, such as worries about the future). Eight studies also examined bedtime levels of cortisol, but most studies found no relationship to acute stressors or acute stress. Increased levels of bedtime cortisol are related to a flatter diurnal cortisol slope [20] and therefore those findings are in accordance to the results concerning diurnal cortisol slope.

EMA studies measuring cardiovascular outcomes observed typical acute stress responses including increased blood pressure, HR and decreased HRV. Regarding blood pressure, increased levels were found in situations of high global subjective stress [147, 148], negative social interactions [35, 144–146] and when acute stressors in general and within life domains occurred [136–139]. Evidence that severity of acute stressors at work is associated with blood pressure levels is more mixed. It is notable though that three of those studies used the same questionnaire to measure job demands and only the study with largest sample size and sampling frequency found a significant relationship [35, 141, 143].

In contrast, EMA studies investigating HR and HRV successfully demonstrated relationships to severity of acute stressors at work among healthy study populations. Higher levels of job demands and work efforts were associated with increased HR [143, 153] and stressful emergency operations among firefighters were associated with reduced HRV [162]. Furthermore, EMA studies increased the evidence base for the job-demand-control model, which states that high demands in combination with low control result in high strain jobs and thus chronic stress [184]. Since EMA studies observed significant interactions between job control and job demands for HR and blood pressure on within-subject levels [141–143], not only chronic but also acute stress during work might be explained by the job-demand-control model.

Also EMA studies examining global subjective stress successfully demonstrated relationships to HR and HRV among healthy study populations [148, 152, 154, 155]. Furthermore, respectively one study investigating moderation due to chronic stress and PTSD only found significant relationships among individuals with high levels of chronic stress [154] or PTSD [156]. A further study found no moderation of PTSD for heart rate but for blood pressure reactivity [149]. Those results hint to exaggerated stress reactivity of the autonomic nervous system during chronic stress and PTSD, which is at least for PTSD supported by previous non-EMA literature [185]. For cardiovascular stress responses, previous literature was described as more mixed [186]. However, more recent laboratory studies found rather blunted than exaggerated HR and blood pressure responses to acute stressors under chronic stress [55, 56]. Furthermore, exaggerated responses of cardiovascular measures are in contrast to findings of blunted cortisol responses to acute psychosocial stress under PTSD [179, 187] and that stress responses of the autonomic nervous system and HPA axis are interrelated [188]. However, also previous work found disparate responses of the autonomic nervous system and HPA axis among patients with PTSD [189]. Furthermore, low baseline cortisol levels and blunted cortisol responses to acute stress among patients with PTSD might be explained by a hyperresponsive negative feedback loop regarding the HPA axis [19, 54, 185].

For heart rate and HRV no consistent evidence regarding associations with occurrence of acute stressors was found. Most studies allowed time lags of one hour and more between occurrence of acute stressor and measurement of heart rate and HRV. One might assume that those time lags were probably too long to detect stress responses. For example, previous laboratory studies showed that associations between subjective stress and heart rate peak during occurrence of stressors and that heart rate levels recover after five minutes [53, 190]. This goes along with the recommendation of not using time frames of more than 30 minutes when examining cardiovascular outcomes [64].

In contrast to blood pressure, no association was found between severity of stressors regarding social interactions and HR [143, 144, 146, 152]. This suggests that blood pressure might be the better indicator for physiological stress reactivity regarding negative social interactions than HR.

Altogether, the included studies suggest that EMA research designs are more suitable to observe physiological stress reactions if exposure and outcome were measured concurrently using dense time resolution instead of applying daily diary designs. Advantages of studies examining current cortisol and cardiovascular reactivity were frequent or continuous measurements and therefore higher statistical power, reduced time lag between exposure and outcome assessments and reduced risk of recall bias as compared to studies examining aggregate measures of cortisol reactivity. However, even under those circumstances EMA studies were only partially able to demonstrate typical acute stress reactions during daily life. In direct comparison, stress during daily life often includes minor hassles [8] possibly resulting in weaker stress reactions than laboratory stress tests being specifically designed to initiate maximal stress responses, such as the TSST [191]. Furthermore, subjective understandings of stress might differ between participants and frequent lack of reliable and valid measures might have aggravated identification of acute stress as well as comparability of EMA findings [8]. However, it is likely that the multilevel approach of investigating within-subject associations implicitly controlled for variations in personal stress definitions. Moreover, most studies in this review have missed to control for at least two of the variables that were listed as confounders within the quality assessment. This might have also contributed to overall inconclusive findings. Furthermore, stress assessment during daily life may have been challenged by large variety of possible stressors and their infrequent occurrence[15]. In future studies, a broad sampling scheduling strategy may help to better detect stress occurrences in daily life across various life domains. Just like laboratory stress research [53, 192], EMA studies found several inter- and intra-individual moderators of stress reactivity. The large number of variables influencing stress reactivity complicate the measurement of physiological stress reactions, especially in uncontrolled settings during daily life [71].

### Limitations

This systematic review has different limitations that should be taken into account.

On the review level, it should be mentioned that there is no consistent definition of stress. As exposure (in the sense of an independent variable in a causal model), we chose both stressors and subjective stress as the trigger of subsequent or concurrent physiological stress responses although subjective stress already represents a response. While the exposure definition could be criticized from a theoretical stance, it can be noted that it enabled us to include a larger number of studies despite the inconsistent definition of stressors and stress in many of the included studies. In addition, various aspects have hampered the comparability and generalizability of results. In view of the large heterogeneity in study design, analysis and operationalization of acute stressors or stress, we have not performed a meta-analysis. For example, operationalization of acute stressors or subjective stress differed regarding type of stressor (e.g. stressors regarding work, education, current activity, social interactions), scaling (e.g. occurrence of any stressor with a dichotomous scale or number of stressors with a continuous scale) and timing (e.g. acute stress measured within the last 5 minutes or since the previous report). This variation however is likely to influence the strength of association. Furthermore, scales that assessed severity of acute stressors or global subjective stress are likely to measure stronger subjective stress than scales measuring mere occurrence of negative events. Pooled effect measures would therefore be hard to interpret. In addition, most studies were only assessed as having low or moderate quality increasing for example the risk for confounding and recall bias. During meta-analysis, those risks would be combined resulting in unreliable effect measures [193]. Furthermore, we could identify a variety of moderator variables that were in several cases very specific or hard to generalize to other contexts.

On the study level, compliance was overall better for studies examining current salivary cortisol levels. For the rest, there were no huge differences between outcomes regarding quality assessment. It can be stated that instruments for exposure measurement were seldom described to be valid and reliability estimates were often missing. Data collection in EMA studies requires extensive resources, including high time demands for participants and researchers. Therefore, study samples were often small and not representative for the general population or the population of interest (e.g. selection bias). However, due to repeated measurements, sample sizes for within-subject analyses were still high. It is still important, though, that sampling occasions within one participant are representative for his or her daily life. High compliance rates and either frequent time-based sampling schemes of current experiences or repeated end-of-day assessments have likely facilitated representativeness of sampling occasions within included EMA studies. However, end-of-day assessments increase the risk of recall bias.

Although publication bias cannot be excluded, it should be noted that more than half of included studies reported null findings regarding direct associations between acute stressors or stress and physiological outcomes. These studies rarely aimed to study direct associations, but rather focused on moderating variables. Furthermore, “p-hacking” (i.e. fishing for significance) could have contributed to some of the significant findings as most studies tested more than one exposure and/or outcome. Analyzing only two outcomes with strong correlation was previously shown to increase the chance of false significant results twofold [194]. As a result, physiological stress responses might be even more difficult to detect by EMA studies than a first look into the research literature might suggest.

### Implications and recommendations for research and practice

Exaggerated cortisol and cardiovascular reactivity to acute stress was shown to be prospectively associated with an increased risk for cardiovascular diseases [50]. This relationship was mainly investigated in laboratory research [50], but only one of included EMA studies investigated health consequences of increased stress reactivity during daily life [195]. Future EMA studies should thus provide more insights on whether heightened stress reactivity might be per se associated with negative health outcomes or whether it is a combination of both, the experience of a large or frequent number of stressors and amplified reactivity to those stressors. Such research could help develop tailored stress prevention interventions including the individual identification of daily stressors, subsequent training of coping skills (e.g. emotion-focused coping strategies) and reduction of individual stressors if possible (e.g. problem-orientated coping).

In addition, blunted stress reactivity is associated with increased morbidity [50]. Differentiating between blunted, normal and exaggerated stress responses might therefore be important for future research to give reliable information about the quantifiable health impact of the identified moderators of stress reactivity. However, to the best of our knowledge, reference values for normal stress responses are lacking so far [24].

In light of negative health consequences of altered -blunted as well as exaggerated- stress reactions [50], research on modifiable moderators is particularly important to give information on potential starting points for interventions promoting adaptive and healthy coping with stress. EMA studies suggest that physical activity [138], engagement coping [78] or work [141–143, 150] and family environments [103, 115, 129] are possible targets for interventions. Such interventions might especially address individuals experiencing a high amount of psychological stress and having an increased risk for blunted or exaggerated stress reactions. EMA studies suggested that this might include patients suffering from mental disorders [98, 134, 149, 156], spinal cord injury [79], 22q11.2 deletions syndrome [92] and women carrying a premutation of FMR1 [135]. In addition, patient groups having an increased risk to develop cardiovascular disease or other diseases in which stress plays a significant role in the etiology might benefit from such interventions. However, research on those moderators in daily life is still in the beginning and more rigorous testing of those variables seems advisable before implementing corresponding interventions.

## Conclusion

Results suggest that EMA designs are especially suitable for stress research if associations between stressors or stress and physiological reactions are studied concurrently under dense temporal resolution with at least three measurement points per day instead of using daily diary designs. However, even studies performing at least three measurements per day could only partially observe typical acute stress reactions including increased salivary cortisol levels, blood pressure, HR and decreased HRV during daily life. Furthermore, EMA designs appear to be useful to provide preliminary indications of inter- and intra-individual moderators affecting stress reactivity during daily life. Included studies in this review identified moderators such as chronic stress, physical activity, engagement coping, work and family environments and several mental and physical health conditions. Nevertheless, more research on moderator variables in daily life is needed to develop and evaluate interventions promoting adaptive and healthy coping with stress.

## Supporting information

S1 Table Free text search terms and MeSH terms for physiological stress reactions. (PDF)

S2 Table Search in PsycINFO via Ovid. (PDF)

S3 Table Description of studies examining within-subject associations between acute stress and salivary cortisol (nmol/L, log transformed). (PDF)

S4 Table Description of studies examining within-subject associations between acute stress and cardiovascular outcomes. (PDF)

S5 Table Description of studies examining within-subject associations acute between stress and other outcomes. (PDF)

S6 Table Quality assessment. (PDF)

S1 Checklist PRISMA 2020 checklist. (DOCX)
