# Supplementary material for: Physiological reactions to acute stressors and subjective stress during daily life: A systematic review on ecological momentary assessment (EMA) studies
Source: PLoS One. 2022 Jul 27;17(7):e0271996. doi: 10.1371/journal.pone.0271996 (PMC9328558; doi:10.1371/journal.pone.0271996)
Supplement: S1 Table — (PDF) [file pone.0271996.s001.pdf]

S1 Table

Article title: Physiological reactions to acute stressors and subjective stress during daily life: A systematic review on ecological momentary assessment (EMA) studies

Authors: Jeannette Weber, Peter Angerer, Jennifer Apolinário-Hagen

Institute of Occupational-, Social- and Environmental Medicine, Centre for Health and Society, Medical Faculty, Heinrich-Heine-University Düsseldorf, Moorenstraße 5, 40225 Düsseldorf, Germany

S1 Table. Free text search terms and MeSH terms for physiological stress reactions

Stress reactions	Free text search terms	MeSH terms PsycINFO	MeSH terms Pubmed
Autonomic nervous system	autonomic nervous system; autonomic function*; sympathetic nervous system	exp autonomic nervous system/	exp Autonomic Nervous System/
Norepinephrine and epinephrine	*adrenaline; *epinephrine; catecholamine*	exp Catecholamines/	exp Catecholamines/
alpha-Amylase	alpha-amylase	-	exp alpha-Amylases/
Electrodermal activity	electrodermal activity; skin conductance; galvanic skin response	exp Galvanic Skin Response/	exp Galvanic Skin Response/
Hypothalamic-pituitary-adrenal axis	hypothalamic pituitary; HPA axis	exp Hypothalamic Pituitary Adrenal Axis/	exp Hypothalamo-Hypophyseal System/
Cortisol	Cortisol; glucocorticoid*	exp Hydocortisone/	exp Hydrocortisone/
ACTH	adrenocorticotrop*; ACTH	exp Corticotropin/	exp Adrenocorticotropic Hormone
Corticotropin Releasing hormone (CRH)	corticotrop*; CRH	exp Corticotropin Releasing Factor/	exp Corticotropin-Releasing Hormone/
Dehydroepiandrosterone	Dehydroepiandrosterone; DHEA	-	exp Dehydroepiandrosterone/
Other biomarkers of stress	Biomarker* NEAR/1 stress		
Serotonin<br>Dopamine	Serotonin; dopamine; neurotransmitter*; hormone*; monoamine	*Serotonin/	exp Serotonin/
Acetylcholine	acetylcholine	exp Acetylcholine/	exp Acetylcholine/
GABA	GABA; aminobutyric acid	exp gamma aminobutyric acid/	exp gamma-Aminobutyric Acid/
Chromogranin A	chromogranin A	-	exp Chromogranin A/
Brain derived neurotropic factor (BDNF)	brain derived neurotropic factor; brain derived neurotrophic factor; BDNF	exp Brain Derived Neurotrophic factor/	exp Brain-Derived Neurotrophic Factor/
Cardiac troponin T (CTT)	cardiac	-	exp Troponin T/
Neuropeptide Y (NPY)	neuropeptide Y; NPY	exp Neuropeptide Y/	exp Neuropeptide Y/

Orexin A	orexin A	exp Orexin/	exp Orexins/
Oxytocin	oxytocin	exp Oxytocin/	exp Oxytocin/
Physiological reactions			
Brain			
Altered brain activity	electroencephalogra*; EEG; brain NEAR/2 activity; brain waves; brainwaves	Exp Electroencephalography/	exp Electroencephalography/
Eyes			
Pupil diameter	pupil diameter; size NEAR/2 pupil; dilation NEAR/2	exp Pupil Dilation/	-
Eye gaze	pupil; dilatation NEAR/2 pupil; eye gaze; eye track*;	exp Eye Movements/	exp Eye Movements/
Blink rates	blink* rate*; blink* frequency; eye blink*"	-	exp Blinking/
Respiration			
Bronchodilation<br>Rapid breathing	respirat*; breathing; *plethysmograph*	exp Respiration/ OR exp Plethysmography/	exp Respiration/ or exp Plethysmography/ or exp Vital Signs
Muscle			
Muscle tension	musc* tension; musc* tone; musc* tonus; musc* activity; electromyogra*; EMG	exp Muscle Contractions/ OR exp Muscle Relaxation/ OR exp Muscle Tone OR exp Electromyography	exp Muscle Contraction/ or exp Muscle Tonus or exp Electromyography/
Cardiovascular	Cardiovascular; cardiac	exp Cardiovascular Reactivity/	-
Heart rate; heart rate variability	heart rate; electrocardiogra*; ECG	exp Heart Rate/	exp Vital Signs/ or exp Electrocardiography, Ambulatory/ or exp Hemodynamics/
Cardiac output	cardiac	-	exp Hemodynamics/
Blood pressure	blood pressure	exp Blood Pressure/	exp Vital Signs/ or exp Hemodynamics/
Changes of blood circulation	blood flow; blood circulation; *plethysmograph*	exp Blood Flow/	exp Hemodynamics/
Coagulation	*coagulation; *coagulability; clotting; fibrino*; prothrombin time; von Willebrand factor; hemostasis	exp Blood Coagulation/	exp Hemostasis/
Metabolism			
Plasma glucose levels	Glucose; blood sugar	exp Glucose/	exp Glucose/
Thermogenesis			
Skin temperature	Thermogenesis; temperature	exp Body Temperature/	exp Vital Signs/ or exp Body Temperature/
Sweat rate	sweat rate; sweating; sweat production	exp Sweating	exp Body Temperature/
Immune response	immune	exp Immunology/	exp Immune System/
Immunoglobuline A	immunoglobulin*	exp Immunology/	exp Immunoglobulins/
Lysozyme	Lysozyme; muramidase	-	exp Muramidase/
IL-6	cytokine*; interleukin*	exp Immunology/	exp Cytokines/
IL-1ß			
TNF-alpha	tumor necrosis factor; TNF	exp Immunology/	exp Cytokines/
C-reactive protein	C-reactive protein, CRP	-	exp C-Reactive Protein/

Altered blood count	natural killer cell*; NK cell*; leukocyte*; leucocyte*; white blood cell*; lymphocyte*; B cell*; T cell*; monocyte*; CD* cell*; neutrophil*	exp Leucocytes/	exp Leukocytes/
