# Supplementary material for: Physiological reactions to acute stressors and subjective stress during daily life: A systematic review on ecological momentary assessment (EMA) studies
Source: PLoS One. 2022 Jul 27;17(7):e0271996. doi: 10.1371/journal.pone.0271996 (PMC9328558; doi:10.1371/journal.pone.0271996)
Supplement: S2 Table — (PDF) [file pone.0271996.s002.pdf]

S2 Table

Article title: Physiological reactions to acute stressors and subjective stress during daily life: A systematic review on ecological momentary assessment (EMA) studies

Authors: Jeannette Weber, Peter Angerer, Jennifer Apolinário-Hagen

Institute of Occupational-, Social- and Environmental Medicine, Centre for Health and Society, Medical Faculty, Heinrich-Heine-University Düsseldorf, Moorenstraße 5, 40225 Düsseldorf, Germany

S2 Table Search in PsycINFO via Ovid

	Searches	Results
**1**	("ecological momentary assessment" or diary or diaries or "experience sampling" or "ambulatory monitoring" or "event-sampling" or "real-time assessment").mp.	16762
**2**	exp Ecological Momentary Assessment/	971
**3**	1 or 2	16762
**4**	(strain or distress or stress*).mp.	359982
**5**	exp Occupational Stress/ or exp Psychological Stress/	29493
**6**	4 or 5	360084
**7**	("autonomic nervous system" or "autonomic function*" or "sympathetic nervous system").mp.	12093
**8**	exp autonomic nervous system/	10422
**9**	7 or 8	17191
**10**	("adrenaline" or "noradrenaline" or "epinephrine" or "norepinephrine" or "catecholamine*").mp.	22612
**11**	exp Catecholamines/	31793
**12**	10 or 11	43495
**13**	"alpha-amylase".mp.	629
**14**	("electrodermal activity" or "skin conductance" or "galvanic skin response").mp.	9744
**15**	exp Galvanic Skin Response/	2642
**16**	14 or 15	9744
**17**	("hypothalamic pituitary" or "HPA axis" or "cortisol" or "glucocorticoid*" or "adrenocorticotrop*" or ACTH or corticotrop* or CRH or dehydroepiandrosterone or "DHEA").mp.	30990
**18**	exp Hypothalamic Pituitary Adrenal Axis/ or exp Hydrocortisone/ or exp Corticotropin/ or exp Corticotropin Releasing Factor/	15028
**19**	17 or 18	31078
**20**	(biomarker* adj1 stress).mp.	178
**21**	(GABA or "aminobutyric acid" or acetylcholine or serotonin or dopamine or neurotransmitter or hormone or monoamine or "chromogranin A" or "brain derived neurotropic factor" or "brain derived neurotrophic factor" or BDNF or cardiac or "neuropeptide Y" or NPY or "orexin A" or oxytocin).mp.	158450
**22**	exp Acetylcholine/ or exp gamma aminobutyric acid/ or *Serotonin/ or exp Brain Derived Neurotrophic factor/ or exp Neuropeptide Y/ or exp Orexin/ or exp Oxytocin/	34806
**23**	20 or 21 or 22	159346
**24**	(electroencephalogra* or EEG or "brain adj2 activity" or "brain waves" or brainwaves).mp.	64488
**25**	exp Electroencephalography/	28626
**26**	24 or 25	66306
**27**	("pupil diameter" or (size adj2 pupil) or (dilation adj2 pupil) or (dilatation adj2 pupil) or "eye	4514

	gaze" or "eye track" or "blink* rate*" or "blink* frequency" or "eye blink*").mp.	
**28**	exp Pupil Dilation/ or exp Eye Movements/	20216
**29**	27 or 28	23450
**30**	(respirat* or breathing or plethysmograph*).mp.	30516
**31**	exp Respiration/ or exp Plethysmography/	6137
**32**	30 or 31	30518
**33**	("musc* tension" or "musc* tone" or "musc* tonus" or "musc* activity" or electromyogra* or EMG).mp.	20235
**34**	exp Muscle Contractions/ or exp Muscle Relaxation/ or exp Muscle Tone/ or exp Electromyography/	7300
**35**	33 or 34	21582
**36**	(cardiovascular or "heart rate" or electrocardiogra* or ECG or "blood pressure" or "blood flow" or "blood circulation" or photoplethysmograph* or coagulation or hypercoagulation or coagulability or hypercoagulability or clotting or fribrino* or "prothrombin time" or "von Willebrand factor" or hemostasis).mp.	79686
**37**	exp Cardiovascular Reactivity/ or exp Heart Rate/ or exp Blood Pressure/ or exp Blood Flow/ or exp Blood Coagulation/	25573
**38**	36 or 37	79729
**39**	(glucose or "blood sugar").mp.	17664
**40**	exp Glucose/	4527
**41**	39 or 40	17664
**42**	(thermogenesis or temperature or "sweat rate" or sweating or "sweat production").mp.	19325
**43**	exp Body Temperature/ or exp Sweating/	4656
**44**	42 or 43	19621
**45**	(immune or immunoglobulin* or lysozyme or muramidase or cytokine* or interleukin* or "tumor necrosis factor" or TNF or "C-reactive protein" or CRP or "natural killer cell*" or "NK cell*" or leukocyte* or leucocyte* or "white blood cell*" or lymphocyte* or "B cell*" or "T cell*" or monocyte* or "CD* cell*" or neutrophil*).mp.	50256
**46**	exp Immunology/ or exp Leucocytes/	25904
**47**	45 or 46	57521
**48**	physiolog*.mp.	123407
**49**	9 or 12 or 13 or 16 or 19 or 23 or 26 or 29 or 32 or 35 or 38 or 41 or 44 or 47 or 48	520249
**50**	3 and 6 and 49	606
**51**	limit 50 to (dutch or english or german or portuguese)	594
