# Supplementary material for: Physiological reactions to acute stressors and subjective stress during daily life: A systematic review on ecological momentary assessment (EMA) studies
Source: PLoS One. 2022 Jul 27;17(7):e0271996. doi: 10.1371/journal.pone.0271996 (PMC9328558; doi:10.1371/journal.pone.0271996)
Supplement: S3 Table — (PDF) [file pone.0271996.s003.pdf]

S3 Table

Article title: Physiological reactions to acute stressors and subjective stress during daily life: A systematic review on ecological momentary assessment (EMA) studies

Authors: Jeannette Weber, Peter Angerer, Jennifer Apolinário-Hagen

Institute of Occupational-, Social- and Environmental Medicine, Centre for Health and Society, Medical Faculty, Heinrich-Heine-University Düsseldorf, Moorenstraße 5, 40225 Düsseldorf, Germany

S3 Table Description of studies examining within-subject associations between acute stress and salivary cortisol (nmol/L, log transformed)

Study	Study population and setting	Exposure variable (scale range[1])	Sampling schedule per participant: Duration and frequency[2]	Max. time lag between exposure and outcome	Results (direct association between exposure and outcome)
**Almeida et al. 2016 (1)**	131 employees of an IT division in the US; M_{age}=45.2 (SD=6.3); female: 45%	Severity of acute stressors within a life domain: work-family conflict (scale range: 4–20)	4 days; exposure: daily outcome: 2 times/day	same day	Moderation effects of supervisor support were included in the following models:<br><u>Before dinner cortisol:</u> No association (BS: b=-0.022, SE=0.018, p>.10; WS: b=-0.014, SE=0.014, p>.10)<br><u>Bedtime cortisol:</u> No association (BS: b=-0.010, SE=0.020, p>.10; WS: b=0.007, SE=0.017, p>.10)<br><u>Before dinner to bedtime cortisol slope:</u> Positive association (BS: b=0.009, SE=0.009, p>.10; WS: b=0.013, SE=0.007, p<.05)
**Bai et al. 2017 (2)**	47 children in the US; M_{age}=11.3 (SD=1.5, range=8-13); female: 60%	Number of acute stressors in education and regarding social interactions (scale range: 0-5); Severity	6-8 days; exposure: daily outcome: 4 times/day	Diurnal cortisol slope: same day Next-day cortisol at wake-up, bedtime cortisol: previous day	<u>Diurnal cortisol slope:</u> No association with acute stressors regarding social interactions (WS: b=-0.01, SE=0.01, p>.10; BS: b=0.04, SE=0.02, p<.05), acute stressors in education (WS: b=0.003, SE=0.008, p>.10; BS: b=0.01, SE=0.01, p>.10) or interparental conflict (WS: b=0.01, SE=0.01, p<.10; BS: b=0.02, SE=0.04, p>.10).<br><u>Next day cortisol at wake-up:</u> Positive association with acute stressors regarding social interactions (WS: b=0.34, SE=0.17, p<.05; BS: b=0.11, SE=0.32, p>.10) and acute stressors in education (WS: b=0.18, SE0.08, p<.05; BS: b=0.01, SE=0.01, p<.10). No association with interparental conflict (WS: b=-0.01, SE=0.09, p>.10; BS: b=-0.16, SE=0.24, p>.10).

		of acute stressors in family life - interparental conflict (scale range: 1-3)			Bedtime cortisol: No association with acute stressors regarding social interactions (WS: b=-0.05, SE=0.16, p=.760; BS: b=-0.69, SE=0.55, p=.219), acute stressors in education (WS: b=0.11, SE=0.12, p=.382; BS: b=-0.21, SE=0.18, p=.258) or interparental conflict (WS: b=0.04, SE=0.08, p=.652; BS: b=-0.17, SE=0.30, p=.570)
**Barker et al., 2012 (3)**	61 parents of children with and 321 parents of children without serious mental disorders in the US; parents of children with serious mental disorders: M_{age}= 60.07 (SD=10.01), female: 54%; control group: M_{age}= 58.09 (SD=12.88), female: 52%	Number of acute stressors (scale range: 0-7); severity of acute stressors (scale range: 0-28)	4 days; exposure: daily outcome: 4 times/day	Next-day CAR: previous day Diurnal cortisol slope: same day	Next-day CAR: Negative association with number of acute stressors (WS: b=-2.92, SE=1.465, p<.05) and positive association with severity of acute stressors (WS: b=1.26, SE=0.523, p<.05)<br>Diurnal cortisol slope: No association with number of acute stressors (WS: b=-3.22, SE=1.841, p>.05) and positive association with severity of acute stressors (WS: b=1.43, SE=0.660, p<.05)
**Bernstein et al., 2018 (4)**	108 participants in the US; M_{age}= 41.12 (SD= 11.6); female: 75%	Severity of acute stressors regarding social interactions (scale range: 0-6*)	3 days; 6 times/day	concurrent	Current cortisol: No association (WS: b=-0.01, SE=0.01, p>.10; BS: b=-0.004, SE=0.03, p>.10)
**Birditt et al., 2015 (5)**	1736 participants in the US; M_{age}= 56.24 (SD=12.21); female: 57%	Occurrence of acute stressors regarding social interactions: engagement and avoidance of arguments (categorical variable: 1=argument, 2=avoidance,	4 days exposure: daily outcome: 4 times/day	Overall cortisol per day, diurnal cortisol slope: same day and previous day Next-day CAR: previous day	Overall cortisol per day: No association with same day argument (b=0.05, SE=0.03, p>.05), same day avoidance of argument (b=0.03, SE=0.02, p>.05), previous day argument (b=0.02, SE=0.03, p>.05) or previous day argument + avoidance of argument (b=.0.01, SE=0.05, p>.05). Positive association with same day argument + avoidance of argument (b=0.15, SE=0.06, p<.05) and previous day avoidance of argument (b=0.06, SE=0.02, p<.05).<br>Next-day CAR: No association with engagement in arguments (b=-0.01, SE=0.07, p>.05), avoidance of arguments (b=-0.06, SE=0.05, p>.05) or engagement in arguments + avoidance of arguments (b=0.05, SE=0.12, p>.05)<br>Diurnal cortisol slope: No association with same day argument (b=-0.00, SE=0.00, p>.05), same day avoidance of arguments (b=-0.00, SE=0.00, p>.05), same day arguments + avoidance of arguments (b=-0.02, SE=0.01, p>.05), previous day argument (b=-0.00, SE=0.00, p>.05), previous day avoidance of arguments (b=-0.00, SE=0.00, p>.05) or previous day arguments + avoidance of

		3=argument and avoidance, 4=no interpersonal tension)			arguments (b=0.00, SE=0.00, p>.05)
**Birditt et al., 2016 (6)**	197 middle-aged adults (reduced sample size in analysis) in the US; M_{age}= 56.3 (SD=4.8); female: 55%	Occurrence of acute stressors regarding social interactions with children who have physical-emotional or lifestyle-behavioral problems (dichotomous scale: 0/1)	4 days exposure: daily outcome: 3 times/day	Overall cortisol per day, diurnal cortisol slope: same day, previous day Next-day CAR: previous day	Overall cortisol per day: No association with interactions at same (b=0.10, p>.05) and previous day (b=0.12, p>.05) among adults with children who have physical-emotional problems. Among adults with children who have life-style behavioral problems, no association with same day interaction (b=0.03, p>.05) but positive association with previous day interactions (b=0.19, p<.05)<br>Next-day CAR: No association with among adults with children who have physical-emotional problems (b=-0.15, p>.05) or among adults with children who have lifestyle-behavioral problems (b=0.09, p>.05)<br>Diurnal cortisol slope: Negative interactions at same day were associated with steeper decline among adults with children who have physical-emotional problems (b=-0.03, p<.01). No association with negative interactions at previous day (b=-0.01, p>.05) among adults with children having physical-emotional problems. No association with negative interactions at same day (b=-0.01, p>.05) or previous day (b=-0.01, p>.05) among adults with children who have lifestyle-behavioral problems.
**Birditt et al., 2017 (7)**	156 middle-aged adults in the US; M_{age}= 55.85 (SD=4.72); female: 56%	Occurrence of acute stressors regarding social interactions: negative interactions and avoidance of negative interactions with adult child and parent (dichotomous scale: 0/1)	4 days exposure: daily outcome: 3 times/day	Overall cortisol per day, diurnal cortisol slope: same day, previous day Next-day CAR: previous day	Overall cortisol per day: No association with negative interaction with parents at same day (b=-0.07, SE=0.08, p>.05) and previous day (b=-0.06, SE=0.08, p>.05) or with children at same day (b=0.03, SE=0.08, p>.05) and previous day (b=-0.05, SE=0.07, p>.05). No association with avoidance of negative interaction with parents at same day (b=-0.09, SE=0.08, p>.05) and previous day (b=-0.01, SE=0.07, p>.05) and with children at same day (b=0.03, SE=0.07, p>.05) and previous day (b=0.07, SE=0.07, p>.05)<br>Next-day CAR: No association with negative interaction with parents (b=0.22, SE=0.17, p>.05) or with children (b=-0.01, SE=0.16, p>.05). No association with avoidance of negative interaction with parents (b=0.14, SE=0.17, p>.05) and with children (b=-0.05, SE=0.16, p>.05)<br>Diurnal cortisol slope: No association with negative interaction with parents at same day (b=0.01, SE=0.01, p>.05) and previous day (b=0.01, SE=0.01, p>.05), but negative association with negative interactions with children at same day (b=-0.01, SE=0.01, p<.05) and previous day (b=-0.02, SE=0.01, p<.01). No association with avoidance of negative interaction with parents at same day (b=0.00, SE=0.01, p>.05) and previous day (b=0.01, SE=0.01, p>.05) and no association with avoidance of negative interaction with children at previous day (b=0.00, SE=0.01, p>.05). Negative interaction with avoidance of negative interaction with children at same day (b=-0-02, SE=0.01, p<.05)
**Collip et al., 2011 (8)**	60 siblings of patients diagnosed with non-affective	Severity of acute stressors (scale range:	6 days; 10 times/day	Since last prompt	Current cortisol: No association (ß=0.01, 95%CI=-0.013-0.034, p=.37)

	psychotic disorder and 63 controls in the Netherlands; siblings: M_{age}= 28.8 (SD=10.0), female: 63%; controls: 33.3 (SD=10.3) female: 71%	0 - 3)			
**Costanzo et al., 2012 (9)**	111 cancer survivors and 111 controls without prior cancer in the US; M_{age}= 65 (range=35-83); female: 63%	Occurrence of any acute stressor; Occurrence of acute stressors regarding social interactions: engagement and avoidance of argument (dichotomous rating scale: 0/1);	4 days exposure: daily outcome: 4 times/day	Same day	Diurnal cortisol slope No association with occurrence of any acute stressors (cancer: b=0.01, SE=0.01, p>.05; control: b=0.01, SE=0.01, p>.05), engagement of arguments (cancer: b=0.01, SE=0.01, p>.05; control: b=0.01, SE=0.01, p>.05) and avoidance of arguments (cancer: b=0.01, SE=0.01, p>.05; control: b=0.00, SE=0.01; p>.05).<br>Cortisol AUC: No association with occurrence of any acute stressor (b=-7.37, SE=15.97, p>.05) engagement of arguments (b=-31.29, SE=25.59, p>.05) and avoidance of arguments (b=-10.71, SE=25.02, p>.05) among cancer survivors. No association with avoidance of arguments (b=13.68, SE=22.95, p>.05) among controls. Positive association with occurrence of any acute stressor (b=42.49, SE=15.04, p<.01) and engagement of arguments (b=65.42, SE=23.76, p<.01) among controls.
**Crockett & Neff, 2013 (10)**	99 newlywed couples in the US (N=199); husbands M_{age}= 29.13 (SD=5.33); wives: M_{age}=27.24 (SD=4.93) female: 49%	Global subjective stress (scale range: 1-7)	6 days exposure: daily outcome: 2 times/day	Same day	Diurnal cortisol slope: No association among husband (WS: b=-0.02, SE=0.04, 95% CI= -0.077 - 0.040, p>.10) or wives (WS: b=0.01, SE=0.02, 95% CI= -0.029-0.048, p>.10).
**Damaske et al., 2016 (11)**	115 employed people at work in the US; M_{age}=41.21 (SD=11.62); female: 74%	Severity of acute stressors at work: meeting job demands*, resources* (scale range: 1-5*)	2 days; 6 times/day (only time points during work were included in analysis)	concurrent	Current cortisol: Positive association with meeting job demands (b=0.05, SE=0.02, p<.05). No association with resources (b=-0-02, SE=0.02, p>0.1).
**Fischer et al., 2016**	30 women with Fibromyalgia in	Global subjective	14 days; 6 times/ day	concurrent	Current cortisol No association (b=-0.03, p=0.846)

**(12)**	Germany; M_{age}= 50.7 (SD=9.9); female: 100%	stress (scale range: 0-4)			
**Gartland et al., 2014 (13)**	60 participants at working days in England; M_{age}= 28.97 (SD=6.95); female: 66%	Number of acute stressors; severity of acute stressors (scale range: 1-5); Severity of acute stressors: ratio between perceived demands of stressors and perceived resources to meet stressors	3 days; exposure: daily outcome: 4 times/day	Previous day	Next-day AUC of CAR: Negative association with ratio between perceived demands and resources (WS: b=-2.76, SE=1.16, p=02). No association with number of acute stressors (WS: b=0.15, SE=0.46, p.74) and severity of acute stressors (WS: b=-0.01, SE=0.15, p=.96)
**Han et al., 2018 (14)**	340 individuals doing volunteer work in the US; M_{age}=52.46 (SD=11.32); female: 64%	Number of acute stressors (scale range: 1-3)	4 days; exposure: daily outcome: 4 times/day	Same day	Cortisol AUC: Positive association (WS: b=0.03, 95% CI=0.00-0.07, p<.05, BS: b=0.07, 95% CI=-0.02-0.16, p>.05)
**Hanson et al., 2000 (15)**	77 health professionals and office clerks in the Netherlands; M_{age}=36.1; female: 44%	Severity of acute stressors at work: ratio between demands and satisfaction	2 days; 6-10 times/day	140 minutes	Current cortisol: No association (p>.05)
**Hanson & Chen, 2010 (16)**	87 college undergraduate students in Canada; age range=19-25 female: 67%	Number of acute stressors (scale range: 0-16); severity of acute stressors (scale range: 1-5)	5 days; exposure: daily outcome: 4 times/day	Same day	Cortisol AUC: No association with number of acute stressors (WS: b=0.16, SE=0.19, p=.40) or severity of acute stressors (WS: b=0.23, SE=0.40, p=.57)
**Hartley et**	114 mothers who	Number of	4 days;	Previous day	Next-day AUC of CAR: No association with child behavior problems (WS: b=-

**al., 2019 (17); Hartley et al., 2012 (18)**	carry premutation of fragile X and have a biological child with fragile X syndrome in the US; M_{age}=50.3 (SD=7.52, range=36-79); female: 100%	acute stressors in caregiving: child behavior problems (scale range: 0-8); Number of acute stressors (scale range: 0-6)	exposure: daily outcome: 2 times/day		0.06, SE=0.11, p>.09; BS: b=0.29, SE=0.45, p>.09) or acute stressors (WS: b=-0.20, SE=0.13, p>.09; BS: b=0.54, SE=0.78, p>.09)<br>Next-day cortisol at wake up: No association with child behavior problems (n=76, b=0.02, SE=0.03, p>.05)<br>Next-day cortisol 30 minutes post awakening: No association child behavior problems (n=76, b=-0.01, SE=0.02, p>.05)
**Havermans et al., 2011 (19)**	36 bipolar patients and 38 controls in the Netherlands; patients: M_{age}=45.9 (SD=9.7); female: 50% controls: M_{age}=44.4 (SD=11.7); female: 61%	Occurrence of any acute stressors (dichotomous scale: 0/1)	6 days; 10 times/day	90 minutes	Current cortisol: Positive association (b=0.137, SE=0.031, p<.001)
**Heissel et al., 2018 (20)**	42 adolescents in the US; M_{age}= 14.9 (SD=1.87); female: 49%	Occurrence of acute stressors regarding violent crime in neighborhood (dichotomous scale: 0/1)	3 days; exposure: daily outcome: 3 times/day	Previous day	Next day CAR: Positive association (b=0.133, SE=0.058, p<.05)<br>Bedtime cortisol: No association (b=0.005, SE=0.019, p>.10)<br>Next-day waking cortisol: No association (b=-0.048, SE=0.038, p>.10)
**Hoppmann et al., 2006 (21)**	106 employed parents in Germany; M_{age}= 37 (SD=4.9); female: 50%	Number of acute stressors in current activity: goal-hindering activities	6 days; 6 times/day	3 hours	Current cortisol: Positive association with goal-hindering activities (b=0.08, SE=0.08, p<.05)
**Jacobs et al., 2007 (22)**	566 women in Belgium; M_{age}= 27 (SD=8, range=18-61); female: 100%	Severity of acute stressors in current activity (scale range: 1-7)	5 days; every 90 minutes	Severity of acute stressors in current activity and in social interactions:	Current cortisol: Positive association with severity of acute stressors in current activity (ß=0.01, SE= 0.005, p<.05) and social interactions (ß=0.02, SE=0.005, p<.001). No association with severity of acute stressors (ß=0.01, SE=0.009, p>.05)

		and in social interactions (scale range: 1-7); severity of acute stressors (scale range: 0-3)		concurrent; Severity of acute stressors: 90 minutes	
**Johnson et al., 2021 (23)**	115 working adults in the US; M_{age}= 41.23 (SD=11.87, range 19-63); female: 76%	Global subjective stress (scale range: 0-4)	3 days; 6 times/day	concurrent	Current cortisol: Positive association (b=0.03, SD=0.014, p=0.023)
**Kalpakjian et al., 2009 (24)**	25 individuals with spinal cord injury (SCI) and 26 individuals without SCI in the US; SCI: M_{age}=46.0 (SD=11.28), female: 36%; no SCI: M_{age}=46.0 (SD=11.5), female: 42%	severity of acute stressors: overall and in current activity (scale range: 0-6)	2 days; 5 times/day	severity of acute stressors in current activity and social interaction, global subjective stress: concurrent; severity of acute stressors overall: 2 hours	Moderation effects of spinal cord injury were included in those models<br>Current cortisol: No association with severity of acute stressors in current activity (b=-0.005, SE=0.016, p>.05) and overall (b=-0.009, SE=0.015, p>.05). Among participants with spinal cord injury, negative association with severity of acute stressors in social interaction and global subjective stress.
**Keneski et al., 2018 (25)**	107 newlywed couples in the US; husbands: M_{age}=29.1 (SD=5.3); wives: M_{age}=27.2 (SD=4.9)	Number of acute stressors in marriage (scale range: 0-9) and outside marriage (scale range: 0-5)	6 days; exposure: daily outcome: 2 times/day	Same day	Diurnal cortisol slope: Negative association with number of acute stressors in marriage (WS: b=-0.04, SE=0.015, 95% CI = -0.006 - -0.003, p=.028; BS: b=-0.01, SE=0.057, 95% CI = -0.12-0.10, p=.834). No association with number of acute stressors outside marriage (WS: b=0.05, SE=0.028, 95% CI = -0.004 - 0.11, p=.071)
**Lazarides et al. 2020 (26)**	152 pregnant women in the US: Mage=27.8 (SD=5.5)	Global subjective stress (scale range: 0-4)	12 days; exposure: hourly outcome: 5 times/day	Current cortisol: 1 hour; Cortisol AUC: same day	Current cortisol: Positive association (WS: b=0.03, SE=0.014, p=0.031; BS: b=0.023, SE=0.046, p=0.583)<br>Cortisol AUC: Positive association (WS: b=0.038, SE=0.016 p=0.035, BS: b=0.019, SE=0.043, p=0.32)

**Linz et al., 2018 (27)**	289 participants in Germany; M_{age}=40.6 (SD=9.3); female: 60%	Occurrence of acute stressors (dichotomous scale: 0/1); Global subjective stress (scale range: 1-20)	2 days; 4 times/day	2 hours	Moderation effects of affect, arousal and though content were included in the model:<br>Current cortisol: Negative association with occurrence of acute stressors (b=-0.36, SE=0.175, 95% CI=-0.70 - -0.01, p=0.041). No association with global subjective stress.
**Lippold et al., 2016 (28)**	126 children in the US; age range=9-17; female: 55%	Number of acute stressors (scale range: 0-5)	4 days; exposure: daily outcome: 4 times/day	Cortisol before dinner, at bedtime and evening slope: same day; CAR: previous day	Cortisol before dinner: No association (WS: b=-0.02, SE=0.05, p<.10; BS: b=0.17, SE=0.1, p>0.10)<br>Cortisol at bedtime: No association (WS: b=0.09, SE=0.04, p<.10; BS: b=0.14, SE=0.09, p>0.10)<br>Next-day CAR: No association<br>Evening slope of cortisol: Positive association (WS: b=0.20, SE=0.08, p<.05; BS: b=-0.04, SE=0.11, p>0.10)
**Lippold et al., 2016 (29)**	126 children in the US; age range=9-17; female: 55%	Severity of acute stressors in family life (scale range: 1-3)	4 days; exposure: daily outcome: 4 times/day	Same day	Cortisol before dinner: No association (WS: b=-0.01, SE=0.10, p>.10; BS: b=0.14, SE=0.10, p>0.10)<br>Cortisol at bedtime: No association (WS: b=0.01, SE=0.10, p>.10; BS: b=0.18, SE=0.1, p>0.10)<br>Evening slope of cortisol: No association (WS: b=0.06, SE=0.06, p>.10; BS: b=0.07, SE=0.08, p>0.10)
**Liu et al., 2018/Liu 2017 (30, 31)**	165 family caregivers caring for someone with dementia in the US; M_{age}=61.99 (SD=10.70); female: 88%	Number of acute stressors in caregiving (scale range: 0-19); Number of acute non-care related stressors (scale range: 0-8)	8 days; exposure: daily outcome: 5 times/day	Same day	Diurnal cortisol slope: No association with non-care related acute stressors (WS: b=0.09, SE=0.07, p>.05; BS: b=-0.11, SE=0.19, p>.05) or care-related stressors (WS: b=-0.01, SE=0.02 p>.05; BS: b=0.00, SE=0.02, p>.05)
**Lee et al., 2019 (32)**	374 high school students in the US; M_{age}= 14.2 (SD=0.5); female: 51%	Severity of acute stressors in social interactions and education (scale range: 1-10)	6-10 days; daily	1 day	Moderation effect of implicit theories of intelligence were included in the model:<br>Current cortisol: Negative association with previous day severity of acute stressors in education (WS: b=-1.06, SE=0.34, ß=-0.12, p=.002; BS: b=-0.21, SE=0.33, ß=-0.03, p=.529). No association with previous day severity of acute stressors in social interactions (WS: b=0.18, SE=0.29, ß=0.02, p=.536).
**O'Connor et al., 2020**	142 individuals who have	Number of acute	7 days; exposure:	Same day	Moderation effect of vulnerability to suicide was included in the model:<br>Diurnal cortisol slope: No association with acute stressors (WS: b=-0.009,

**(33)**	attempted a suicide or with suicidal ideation and control group in UK; M_{age}=28 (SD=9.3); female: 70%	stressors (scale range: 0-8): Global subjective stress (scale range: 0-16)	daily outcome: 2 times/day		SE=0.021, p=.645) and global subjective stress (WS: b=0.006, SE=0.011, p=.537)
**Peeters et al., 2003 (34)**	84 individuals with major depressive disorders or healthy controls in the Netherlands; major depressive disorders: M_{age}=40 (SD=11), female: 58%; controls: M_{age} =44 (SD=12); female: 59%	Occurrence of any acute stressors	6 days; 10 times/ daily	90 minutes	Current cortisol: No association in individuals with major depressive disorders (b=0.005, SE=0.031). Positive association in healthy controls (b=0.109, SE=0.028).
**Polenick et al., 2021 (35)**	93 middle-aged to old adults in the US; M_{age}=67.77 (SD=14.57, range 40-95)	Number of acute stressors regarding social interactions (scale range: 0-10)	5 days; exposure: daily outcome: 4 times/day	Overall cortisol levels per day: same day, previous day<br>Next-day awakening response of cortisol: previous day<br>Wake-Evening slope of cortisol: same day and previous day	Overall cortisol levels per day: No association at same day (b=-0.033, SE=0.03, p>.05) or previous day (b=-0.070, SE=0.04, p>.05)<br>Next-day awakening response of cortisol: No association (WS: b=0.175, SE=0.10, p>.05)<br>Wake-Evening Slope of cortisol: No association with same day (WS: b=-0.001, SE=0.00, p>.05) or previous day (WS: b=0.005, SE=0.00, p>.05)
**Pollard et al., 1996 (36)**	104 participants in UK; Male: 49%, M_{age}= 38.0 (SD=7.53); female: 51%, M_{age}= 37.9 (SD=7.00)	Severity of acute stressors at work (scale range: 1-5)	3 days; daily	3 hours	Current cortisol: No association in women (b=-0.01, p=0.19, F=1.76) or men.
**Proulx, 2015 (37)**	162 older adults in the US; female: 40%, M_{age} =74.42	Number of acute stressors	4 days; exposure: daily	Same day	Cortisol AUC: No association (BS: b=0.60, SE=0.54, p>.10; WS: b=-0.08, SE=-0.08, p>.10)

	(SD=5.82); men: M_{age} =78.85 (SD=5.33)	(scale range: 0-7)	outcome: 4 times/day		
**Qu et al., 2020 (38)**	95 Chinese American school children in the US: M_{age} =13.7 (SD=1.38, range:11-18); female: 50%	Number of acute stressors regarding education (scale range: 0-5)	4 days exposure: daily outcome: 4 times/day	Same day	Cortisol AUC: Positive association (WS: b=38.18, SE=6.71, p<.001)
**Savla et al., 2013 (39)**	28 spouse care partners of individuals with mild cognitive impairment in the US; M_{age}= 72.9 (SD=6.82); female: 90%	Occurrence of any acute stressor in caregiving: Memory- and behavior related problems of spouse - restless behavior, mood disturbances, memory-related problems; Severity of acute stressors regarding social interactions (dichotomous scale: 0/1)	4 days; exposure: daily outcome: 5 times/day	Same day	Diurnal cortisol slope: Restlessness behavior was associated with a steeper decline of cortisol throughout the day (main effect: b=-0.42, SE=0.15, p<.01; interaction effect with daily decline of cortisol: b=0.02, SE=0.01, p>.10). Mood-related problems were associated with an elevated slope of cortisol throughout the day (main effect: b=0.36, SE=0.13, p<.01; interaction effect with daily decline of cortisol: b=0.03, SE=0.01, p<.05). No association with memory-related problems (main effect: b=-0.17, SE=0.10, p>.10; interaction effect with daily decline of cortisol: b=0.01, SE=0.01, p>.10) and severity of acute stressors regarding social interactions (main effect: b=-0.03, SE=0.10, p>.10; interaction effect with daily decline of cortisol: b=0.01, SE=0.01, p>.10)
**Savla et al., 2011 (40)**	30 spouse care partners of individuals with mild cognitive impairment in the US; M_{age}= 72.6 (SD=6.91); female: 90%	Occurrence of acute stressors in caregiving: Behavior related problems of spouse (dichotomous scale: 0/1)	4 days; exposure: daily outcome: 5 times/day	Same day	Diurnal cortisol slope: No association with behavioral problems after waking (b=0.06, SE=0.15, p>.05) and in the evening (b=0.12, SE=0.13, p>.05). Negative association with behavioral problems during the day (main effect: b=-0.08, SE=0.11, p<.05; interaction effect with daily decline of cortisol: b=0.03, SE=0.01, p<.05)
**Savla et**	127 adults	Occurrence	4 days	Same day	Cortisol AUC: Positive association with acute stressors at work (b=0.20, SE=0.07,

**al., 2018 (41)**	providing support to their parents in the US; M_{age}= 56.27 (SD=11.79); female: 61%	of acute stressors at home, at work and in friendship networks (dichotomous scale: 0/1)	exposure: daily outcome: 4 times/day		p<.01). No association with acute stressors at home (b=0.05, SE=0.07, p>0.05) or in friendship networks (b=0.04, SE=0.07, p>.05).
**Saxbe et al., 2008 (42)**	30 couples from two-earner middle-class families (N=60) in the US; M_{age}= 41 (range=28-58); female: 50%	Severity of acute stressors at work: job demands and regarding social interactions at work (scale range: 1-4)	3 days; daily	Same day	Evening cortisol (before bed): Negative association with job demands in men (WS: b=-0.40, SE=0.17, p<.05) and women (WS: b=-0.33, SE=0.17, p<.05). Positive association with negative social interactions at work in men (WS: b=0.84, SE=0.33, p<.05) but not in women (WS: b=-0.78, SE=0.17, p>.05).
**Seaton et al., 2021 (43)**	93 black American students; M_{age}= 22 (range 17-56); female: 78%	Number of acute stressors regarding discrimination (scale range: 0-2)	3 days; exposure: daily outcome: 3 times/day	Next-day cortisol awakening response, Next-day morning cortisol: previous day Evening cortisol, Cortisol AUC: Same day	Evening cortisol (before bed): Positive association (WS: B=0.626, SE=0.299, p<.05)<br>Cortisol AUC: Positive association (WS: B=3.634, SE=1.099, p<.01)<br>Next-day cortisol awakening response: Positive association (WS: B=-0.122, SE=0.040, p<.001)<br>Diurnal slope of cortisol: No association (WS: B=0.005, SE=0.008, p>.05)<br>Next-day morning cortisol: No association (WS: B=0.310, SE=0.341, p>.05)
**Schlotz et al., 2006 (44)**	71 participants in Germany; M_{age}= 52.6 (SD=16.3); female: 56%	Severity of acute stressors at work: performance pressure, task failure (scale range: 0-3)	2 days; 3 times/day	1 hour	Current cortisol: Positive association with performance pressure (WS: b=0.078, SE=0.034, p<.05; BS: b=0.024, SE=0.037, p>.10). No association with task failure (WS: b=-0.031, SE=0.036, p>.05; BS: b=-0.124, SE=0.046, p<.01)
**Seltzer et al., 2010 (45)**	86 mothers of children with autism spectrum disorders in the US; M_{age}=53.9	Number of acute stressors in caregiving: Behavior	4 days exposure: daily outcome: 2 times/day	Previous day	Next day CAR: No association with behavior problems of child with autism spectrum disorders (b=0.617, SE=0.647, p>.10)

	(SD=8.5); female: 100%	problems of child with autism spectrum disorders (scale range: 0-7)			
**Sladek et al., 2019 (46)**	206 Latino(a)/Hispanic high school seniors being accepted to a public university in the US; M_{age}=18.1; female: 64%	Occurrence of ongoing compared to completed acute stressor (dichotomous scale: 0 = completed, 1 = ongoing); severity of acute stressors (scale range: 0-10)	3 days; 5 times/day	1 hour	Current cortisol: Positive association with occurrence of ongoing acute stressors (b=0.018, p<.01, SE=0.006). No association with severity of acute stressors neither when controlled (b=-0.008, SE=0.007, p>.05) nor when not controlled for ongoing or completed stress (b=-0.009, SE=0.007, p>.05).
**Sladek et al., 2016 (47)**	63 first year college students in the US; M_{age}=18.9 (SD=0.5, range=17-19); female: 78%	Severity of acute stressors (scale range: 0-3)	3 days; 5 times/ day	1 hour	Current cortisol: No association (WS: ß=0.03, SE=0.02, p>.10)
**Sladek et al., 2020 (48)**	61 third year college students in the US; M_{age}= 20.91 (SD=0.36); female: 75%	Severity of acute stressors (scale range: 0-4)	3 days; exposure: 5 times/day → rating collated to one mean-day level outcome: 5 times/day	Previous day	Moderation effects of rumination were included in the following models:<br>Next-day cortisol at wake-up: No association (WS: b=0.05, SE=0.08, p>.10; BS: b=0.30, SE=0.12, p<.05)<br>Next-day CAR: No association (WS: b=-0.07, SE=0.12, p>.10; BS: b=-0.14, SE=0.09, p>.10)<br>Next-day cortisol diurnal slope: No association (WS: b=-0.01, SE=0.01, p>.10; BS: b=-0.004, SE=0.01, p>.10).
**Slatcher et al., 2010 (49)**	37 couples with young children (N=74) in the US; husbands: M_{age}=35.6 (SD=4.6); wives: M_{age}=34.5	Global subjective stress (scale range: 0-4)	3 days; exposure: daily outcome: 6 times/day	Previous day	Cortisol slope since waking: No association (husbands: b=0.012, SE=0.013, p=0.37; wives: b=0.015, SE=0.013, p=.28).<br>Cortisol slope since waking^2: No association (husbands: b=-0.001, SE=0.001, p=.36; wives: b=-0.001, SE=0.001, p=.40).

	(SD=4.1)				
**Smyth et al., 1998 (50)**	60 employed and 60 unemployed adults in the US; M_{age}=36.7 (SD=12.0) female: 71%	Occurrence of prior, current and anticipated acute stressors (dichotomous scale: 0/1)	2 days; 6 times/day	prior acute stressor: 145 minutes; current acute stressor: 30 minutes	Current cortisol: No association with prior acute stressors (F(1,1880)=0.4, p>.50, b^3=0.41). Positive association with current acute stressors (F(1, 1740)=6.2, p<.05, b^3=1.18) and anticipation of acute stressors (F(1,1882)=4.4, p<.05, b^3=0.80).
**Smyth et al., 2017 (51)**	115 working adults in the US; M_{age}=41.2 (SD=11.87); female: 76%	Occurrence of any acute stressor (dichotomous scale: 0/1);	3 days; 6 times/ day	Since last prompt	Moderation effect of life satisfaction was included in the model<br>Current cortisol: No association (b=-0.04, SE=0.08, p>.10)
**Stawski et al., 2013 (52)**	1694 participants in the US; M_{age}=56 (SD=12, range =33-84); female: 57%	Occurrence of any acute stressor (dichotomous scale: 0/1)	4 days exposure: daily outcome: 4 times/day	Same day	Cortisol before lunch: Positive association (WS: b=0.36, SE=0.17, p<.05; BS: b=0.04, SE=0.48, p>.05)<br>Cortisol AUC: Positive association (WS: b=6.30, SE=2.14, p<.01; BS: b=10.20, SE=7.29, p>.05)<br>Linear diurnal cortisol slope: BS association (WS: b=-0.07, SE=0.07, p>.05; BS: b=-0.31, SE=0.14, p<.05).<br>Quadratic diurnal cortisol slope: BS association (WS: b=0.002, SE=0.004, p>.05; BS: b=0.02, SE=0.01, p<.05)<br>Before bed cortisol: No association (WS: b=0.09, SE=0.14, p>.05; BS: b=0.19, SE=0.43, p>.05)
**Stoffel et al., 2021 (53)**	60 working adults in Germany; M_{age}=36.17 (SD=11.61, range 19-60) ; female: 33%	Severity of acute stressors regarding social interactions (scale range: 1-100*); Global subjective stress (scale range: 1-100)	4 days; 6 times/day	Current cortisol: 5.5 hours; Average cortisol levels/day: same day	Current cortisol: No association with severity of acute stressors regarding social interactions (WS: b=-0.001, SE= 0.001, p = 0.384, BS: b=0.001, SE= 0.005, p=0.821) and global subjective stress (WS: p>.05)<br>Average cortisol levels/day: Negative association with severity of acute stressors regarding social interactions (WS: b=-0.006, SE=0.002, p=0.013) and positive association with global subjective stress (WS: b = 0.006, SE = 0.002, p = 0.003, in this model an interaction with occurrence of social contact was included).
**Vaessen et al., 2018 (54)**	187 individuals diagnosed with psychotic disorder, non-psychotic first-degree relatives and healthy controls in the Netherlands;	Severity of acute stressors in current activity (scale range: 1-7)	6 days; 10 times/ day	concurrent	Current cortisol: No main effects reported

	M_{age}=42.2, female: 51%				
**van Eck et al., 1996 (55)**	87 male employees in the Netherland; M_{age}= 42.1 (range=27-57); female: 0%	Occurrence of any acute stressor (dichotomous scale: 0/1)	5 days; 10 times/ day	90 minutes	Current cortisol: Positive association (b=.033, SE=0.013, p<.01)
**van der Linden et al., 2021 (56)**	101 patients with autism spectrum disorder and controls without developmental or psychiatric disorders in the Netherlands; patient group: M_{age}=41.1 (SD=12.9, range: 18-64), female: 52%, control: M_{age}=35.5 (SD=12.2, range 18-63), female: 51%	Severity of acute stressors regarding activities (scale range: 1-7), events (scale range: 0-3) and social interactions (scale ragen: 1-7)	10 days; 10 times/day	Severity of acute stressors regarding activities and social interactions : concurrent; severity of acute stressors regarding events: since last prompt	Current cortisol (in those models interactions with gender and group were included): No association with severity of acute stressors regarding activities (WS: B=0.02, SE=0.02, p=0.314), events (WS: B=0.04, SE=0.03, p=0.120) and social interactions (WS: B=0.04, SE=0.02, p=0.090)
**van Duin et al., 2019 (57)**	51 individuals with 22q11.2 deletion syndrome and healthy controls in the Netherlands; M_{age}=36.8, female: 69%	Severity of acute stressors in current activity (scale range: 1-7)	6 days; 10 times/ day	concurrent	Current cortisol: Trend of positive association (overall: b=0.026, SE=0.013, 95% CI=-0.00 - -0.05, p=.051; healthy controls: b=0.03, SE=0.01, 95% CI=-0.00 - -0.05, p=.051; 22q11.2 deletion syndrome: b=-0.02, SE=0.01, 95% CI=-0.05 - 0.01, p=.22)
**Volmer & Fritsche, 2016 (58)**	83 health care professionals at work in Germany; M_{age}= 40.76 (SD=11.84); female: 63%	Occurrence of any acute stressor at work	3 days; 3-5 times/day	Same day	Cortisol AUC: No association (b=0.163, SE=0.52, p>.05)<br>Cortisol after finishing work: No association (b=0.063, SE=0.07, p>.05)
**Wong et al., 2014 (59)**	239 mothers of children with developmental disorder and control group in the US; mothers of children without	Occurrence of any acute stressor at work (dichotomous scale: 0/1)	4 days; daily	Previous day	Next-day cortisol at wake up: No association (WS: b=0.048, SE=0.047, p>0.10; BS: b=-0.028, SE=0.140, p>.10)

	developmental disorder: M_{age}=46.2 (SD=7.1); control group: M_{age}=49.9 (SD=6.3)				
**Wong et al., 2012 (60)**	82 mothers of children with autism in the US; M_{age}= 53.4 (SD=8.4)	Number of acute stressors (scale range: 0-7); severity of acute stressors (scale range:1-28)	4 days; daily	Previous day	Next-day cortisol at wake-up: No association with number of acute stressors (WS: b=0.011, SE=0.031, p>.10, BS: b=-0.059, SE=0.052, p>.10) and severity of acute stressors (WS: b=-0.012, SE=0.013, p>.10, BS: b=-0.002, SE=0.019, p>.10)
**Wong & Shobo, 2016 (61); Wong & Shobo, 2017 (62)**	435 midlife and older workers and retirees in the US; Overall sample: M_{age}=64.1, female: 55%; Sample of retirees: n=253, M_{age}= 66.80 (SD=4.93), female: 55%	Number of acute stressors outside of work (scale range: 0-6)	4 days; exposure: daily outcome: 2 times/day	Previous day	Next-day cortisol 30 minutes post awakening: No association with number of acute stressors among the overall sample (WS: b=0.003, SE=0.021, p>.10; BS: b=0.145, SE=0.057, p<.05) and among retirees (WS: b=0.013, SE=0.029, p>.10; BS: b=0.091, SE=0.072, p>.10)<br>Next-day CAR: No association with number of acute stressors among the overall sample (WS: b=-0.317, SE=0.588, p>.10; BS: b=1.082, SE=1.017, p>.10) and among retirees (WS: b=-0.012, SE=0.028, p>.10; BS: b=0.060, SE=0.073, p>.10)
**Wright et al., 2021 (63)**	95 school children in Canada; M_{age}=10.80 (SD=0.72, range: 9-11); female: 49%	Severity of acute stressors regarding social interactions (scale range: 1-7*)	4 days; Exposure: 4 times/day outcome: 5 times/day	20 minutes	Current cortisol: Positive association (WS: b=0.014, p=0.038)

Notes. Abbreviations: AUC = area under the curve, b= unstandardized regression coefficient, ß = standardized regression coefficient, BS = between-subject effect, CAR = cortisol awakening response, SD = standard deviation, SE = standard error, WS= Within-subject effect; [1] Higher values correspond to more stress. If higher values correspond to lower stress, those scale ranges will be marked by an asterisk;[2] only measurement points, which were also included in analyses will be taken into account; [3] corresponds to cortisol values not being log transformed; * higher values correspond to lower stress

References

1. Almeida DM, Davis KD, Lee S, Lawson KM, Walter K, Moen P. Supervisor Support Buffers Daily Psychological and Physiological Reactivity to Work-to-Family Conflict. J Marriage Fam. 2016;78:165-79.

2. Bai S, Robles TF, Reynolds BM, Repetti RL. Children's diurnal cortisol responses to negative events at school and home. Psychoneuroendocrinology. 2017;83:150-8.

3. Barker ET, Greenberg JS, Seltzer MM, Almeida DM. Daily Stress and Cortisol Patterns in Parents of Adult Children With a Serious Mental Illness. Health Psychol. 2012;31:130-4.

4. Bernstein MJ, Zawadzki MJ, Juth V, Benfield JA, Smyth JM. Social interactions in daily life: Within-person associations between momentary social experiences and psychological and physical health indicators. J Soc Pers Relat. 2018;35:372-94.

5. Birditt KS, Nevitt MR, Almeida DM. Daily interpersonal coping strategies: Implications for self-reported well-being and cortisol. J Soc Pers Relat. 2015;32:687-706.

6. Birditt KS, Kim K, Zarit SH, Fingerman KL, Loving TJ. Daily interactions in the parent-adult child tie: Links between children's problems and parents' diurnal cortisol rhythms. Psychoneuroendocrinology. 2016;63:208-16.

7. Birditt KS, Manalel JA, Kim K, Zarit SH, Fingerman KL. Daily interactions with aging parents and adult children: Associations with negative affect and diurnal cortisol. J Fam Psychol. 2017;31:699-709.

8. Collip D, Nicolson N, Lardinois M, Lataster T, van Os J, Myin-Germeys I. Daily cortisol, stress reactivity and psychotic experiences in individuals at above average genetic risk for psychosis. Psychol Med. 2011;41:2305-15.

9. Costanzo ES, Stawski RS, Ryff CD, Coe CL, Almeida DM. Cancer survivors' responses to daily stressors: Implications for quality of life. Health Psychol. 2012;31:360-70.

10. Crockett EE, Neff LA. When receiving help hurts: Gender differences in diurnal cortisol responses to spousal support. Soc Psychol Personal Sci. 2013;4:190-7.

11. Damaske S, Zawadzki MJ, Smyth JM. Stress at work: Differential experiences of high versus low SES workers. Soc Sci Med. 2016;156:125-33.

12. Fischer S, Doerr JM, Strahler J, Mewes R, Thieme K, Nater UM. Stress exacerbates pain in the everyday lives of women with fibromyalgia syndrome--The role of cortisol and alpha-amylase. Psychoneuroendocrinology. 2016;63:68-77.

13. Gartland N, O'Connor DB, Lawton R, Bristow M. Exploring day-to-day dynamics of daily stressor appraisals, physical symptoms and the cortisol awakening response. Psychoneuroendocrinology. 2014;50:130-8.

14. Han SH, Kim K, Burr JA. Stress-buffering effects of volunteering on salivary cortisol: Results from a daily diary study. Soc Sci Med. 2018;201:120-6.

15. Hanson EK, Maas CJ, Meijman TF, Godaert GL. Cortisol secretion throughout the day, perceptions of the work environment, and negative affect. Ann Behav Med. 2000;22:316-24.

16. Hanson MD, Chen E. Daily stress, cortisol, and sleep: The moderating role of childhood psychosocial environments. Health Psychol. 2010;29:394-402.

17. Hartley SL, DaWalt LS, Hong J, Greenberg JS, Mailick MR. Positive Emotional Support in Premutation Carrier Mothers of Adolescents and Adults With Fragile X Syndrome: Gene by Environment Interactions. Am J Intellect Dev Disabil. 2019;124:411-26.

18. Hartley SL, Seltzer MM, Hong J, Greenberg JS, Smith L, Almeida D, Coe C, Abbeduto L. Cortisol response to behavior problems in FMR1 premutation mothers of adolescents and adults with fragile X syndrome: A diathesis-stress model. Int J Behav Dev. 2012;36:53-61.

19. Havermans R, Nicolson NA, Berkhof J, deVries MW. Patterns of salivary cortisol secretion and responses to daily events in patients with remitted bipolar disorder. Psychoneuroendocrinology. 2011;36:258-65.

20. Heissel JA, Sharkey PT, Torrats-Espinosa G, Grant K, Adam EK. Violence and Vigilance: The Acute Effects of Community Violent Crime on Sleep and Cortisol. Child Dev. 2018;89:e323-e31.

21. Hoppmann CA, Klumb PL. Daily goal pursuits predict cortisol secretion and mood states in employed parents with preschool children. Psychosom Med. 2006;68:887-94.

22. Jacobs N, Myin-Germeys I, Derom C, Delespaul P, van Os J, Nicolson N. A momentary assessment study of the relationship between affective and adrenocortical stress responses in daily life. Biol Psychol. 2007;74:60-6.

23. Johnson JA, Zawadzki MJ, Jones DR, Reichenberger J, Smyth JM. Intra-individual Associations of Perceived Stress, Affective Valence, and Affective Arousal with Momentary Cortisol in a Sample of Working Adults. Annals of Behavioral Medicine. 2021;22:22.

24. Kalpakjian CZ, Farrell DJ, Albright KJ, Chiodo A, Young EA. Association of daily stressors and salivary cortisol in spinal cord injury. Rehabil Psychol. 2009;54:288-98.

25. Keneski E, Neff LA, Loving TJ. The Importance of a Few Good Friends: Perceived Network Support Moderates the Association Between Daily Marital Conflict and Diurnal Cortisol. Soc Psychol Personal Sci. 2018;9:962-71.

26. Lazarides C, Ward EB, Buss C, Chen WP, Voelkle MC, Gillen DL, Wadhwa PD, Entringer S. Psychological stress and cortisol during pregnancy: An ecological momentary assessment (EMA)-Based within- and between-person analysis. Psychoneuroendocrinology. 2020;121:104848.

27. Linz R, Singer T, Engert V. Interactions of momentary thought content and subjective stress predict cortisol fluctuations in a daily life experience sampling study. Sci Rep. 2018;8:15462.

28. Lippold MA, Davis KD, McHale SM, Buxton OM, Almeida DM. Daily stressor reactivity during adolescence: The buffering role of parental warmth. Health Psychol. 2016;35:1027-35.

29. Lippold MA, McHale SM, Davis KD, Almeida DM, King RB. Experiences With Parents and Youth Physical Health Symptoms and Cortisol: A Daily Diary Investigation. J Res Adolesc. 2016;26:226-40.

30. Liu Y. Aging, caregiving, health and well-being (Doctoral dissertation, The Pennsylvania State University, Pennsylvania, USA). 2017; Available from: https://etda.libraries.psu.edu/files/final_submissions/11686.

31. Liu Y, Almeida DM, Rovine MJ, Zarit SH. Modeling cortisol daily rhythms of family caregivers of individuals with dementia: Daily stressors and adult day services use. J Gerontol B Psychol Sci Soc Sci. 2018;73:457-67.

32. Lee HY, Jamieson JP, Miu AS, Josephs RA, Yeager DS. An entity theory of intelligence predicts higher cortisol levels when high school grades are declining. Child Dev. 2019;90:e849-e67.

33. O'Connor DB, Branley-Bell D, Green JA, Ferguson E, O'Carroll RE, O'Connor RC. Effects of childhood trauma, daily stress, and emotions on daily cortisol levels in individuals vulnerable to suicide. J Abnorm Psychol. 2020;129:92-107.

34. Peeters F, Nicholson NA, Berkhof J. Cortisol Responses to Daily Events in Major Depressive Disorder. Psychosom Med. 2003;65:836-41.

35. Polenick CA, Birditt KS, Turkelson A, Perbix EA, Salwi SM, Zarit SH. Daily Social Interactions and HPA Axis Activity Among Midlife and Older Adults. Gerontologist. 2021;61:897-906.

36. Pollard TM, Ungpakorn G, Harrison GA, Parkes KR. Epinephrine and cortisol responses to work: A test of the models of Frankenhaeuser and Karasek. Ann Behav Med. 1996;18:229-37.

37. Proulx JA. Are There Gender Differences in Stress Response in Older Adults? : Findings from the Normative Aging Study (Doctoral dissertation, Oregon State University, Oregon, USA). 2015; Available from: http://hdl.handle.net/1957/57584.

38. Qu Y, Yang B, Telzer EH. The Cost of Academic Focus: Daily School Problems and Biopsychological Adjustment in Chinese American Families. JOURNAL OF YOUTH AND ADOLESCENCE. 2020;49:1631-44.

39. Savla J, Granger DA, Roberto KA, Davey A, Blieszner R, Gwazdauskas F. Cortisol, alpha amylase, and daily stressors in spouses of persons with mild cognitive impairment. Psychol Aging. 2013;28:666-79.

40. Savla J, Roberto KA, Blieszner R, Cox M, Gwazdauskas F. Effects of daily stressors on the psychological and biological well-being of spouses of persons with mild cognitive impairment. J Gerontol B Psychol Sci Soc Sci. 2011;66:653-64.

41. Savla J, Zarit SH, Almeida DM. Routine support to parents and stressors in everyday domains: Associations with negative affect and cortisol. J Gerontol B Psychol Sci Soc Sci. 2018;73:437-46.

42. Saxbe DE, Repetti RL, Nishina A. Marital satisfaction, recovery from work, and diurnal cortisol among men and women. Health Psychol. 2008;27:15-25.

43. Seaton EK, Zeiders KH. Daily Racial Discrimination Experiences, Ethnic-Racial Identity, and Diurnal Cortisol Patterns Among Black Adults. CULTURAL DIVERSITY & ETHNIC MINORITY PSYCHOLOGY. 2021;27:145-55.

44. Schlotz W, Schulz P, Hellhammer J, Stone AA, Hellhammer DH. Trait anxiety moderates the impact of performance pressure on salivary cortisol in everyday life. Psychoneuroendocrinology. 2006;31:459-72.

45. Seltzer MM, Greenberg JS, Hong J, Smith LE, Almeida DM, Coe C, Stawski RS. Maternal cortisol levels and behavior problems in adolescents and adults with ASD. J Autism Dev Disord. 2010;40:457-69.

46. Sladek MR, Doane LD, Gonzales NA, Grimm KJ, Luecken LJ. Latino adolescents' cultural values associated with diurnal cortisol activity. Psychoneuroendocrinology. 2019;109:104403.

47. Sladek MR, Doane LD, Luecken LJ, Eisenberg N. Perceived stress, coping, and cortisol reactivity in daily life: A study of adolescents during the first year of college. Biol Psychol. 2016;117:8-15.

48. Sladek MR, Doane LD, Breitenstein RS. Daily rumination about stress, sleep, and diurnal cortisol activity. Cogn Emot. 2020;34:188-200.

49. Slatcher RB, Robles TF, Repetti RL, Fellows MD. Momentary work worries, marital disclosure, and salivary cortisol among parents of young children. Psychosom Med. 2010;72:887-96.

50. Smyth J, Ockenfels MC, Porter L, Kirschbaum C, Hellhammer DH, Stone AA. Stressors and mood measured on a momentary basis are associated with salivary cortisol secretion. Psychoneuroendocrinology. 1998;23:353-70.

51. Smyth JM, Zawadzki MJ, Juth V, Sciamanna CN. Global life satisfaction predicts ambulatory affect, stress, and cortisol in daily life in working adults. J Behav Med. 2017;40:320-31.

52. Stawski RS, Cichy KE, Piazza JR, Almeida DM. Associations among daily stressors and salivary cortisol: Findings from the National Study of Daily Experiences. Psychoneuroendocrinology. 2013;38:2654-65.

53. Stoffel M, Abbruzzese E, Rahn S, Bossmann U, Moessner M, Ditzen B. Covariation of psychobiological stress regulation with valence and quantity of social interactions in everyday life: disentangling intra- and interindividual sources of variation. JOURNAL OF NEURAL TRANSMISSION. 2021;128:1381-95.

54. Vaessen T, Kasanova Z, Hernaus D, Lataster J, Collip D, van Nierop M, Myin-Germeys I. Overall cortisol, diurnal slope, and stress reactivity in psychosis: An experience sampling approach. Psychoneuroendocrinology. 2018;96:61-8.

55. van Eck M, Berkhof H, Nicolson N, Sulon J. The effects of perceived stress, traits, mood states, and stressful daily events on salivary cortisol. Psychosom Med. 1996;58:447-58.

56. Rutledge T, Linden W, Davies RF. Psychological risk factors may moderate pharmacological treatment effects among ischemic heart disease patients. Canadian Amlodipine/Atenolol in Silent Ischemia Study (CASIS) Investigators. Psychosomatic Medicine. 1999;61:834-41.

57. van Duin ED, Vaessen T, Kasanova Z, Viechtbauer W, Reininghaus U, Saalbrink P, Vingerhoets C, Hernaus D, Booij J, Swillen A, Vorstman J, van Amelsvoort T, Myin-Germeys I. Lower cortisol levels and attenuated cortisol reactivity to daily-life stressors in adults with 22q11.2 deletion syndrome. Psychoneuroendocrinology. 2019;106:85-94.

58. Volmer J, Fritsche A. Daily negative work events and employees' physiological and psychological reactions. Front Psychol. 2016;7:1711.

59. Wong JD, Mailick MR, Greenberg JS, Hong J, Coe CL. Daily work stress and awakening cortisol in mothers of individuals with autism spectrum disorders or Fragile X syndrome. Fam Relat. 2014;63:135-47.

60. Wong JD, Seltzer MM, Greenberg JS, Hong J, Almeida DM, Coe CL. Stressful life events and daily stressors affect awakening cortisol level in midlife mothers of individuals with autism spectrum disorders. Aging Ment Health. 2012;16:939-49.

61. Wong JD, Shobo Y. The influences of employment status and daily stressors on physiological functioning in a sample of midlife and older adults. J Aging Hum Dev. 2016;83:26-43.

62. Wong JD, Shobo Y. The influences of daily stressors on morning cortisol levels in midlife and older retirees: The moderating roles of age and gender. J Aging Health. 2017;29:858-79.

63. Wright L, Bukowski WM. Gender is Key: Girls' and Boys' Cortisol Differs as a Factor of Socioeconomic Status and Social Experiences During Early Adolescence. Journal of Youth & Adolescence. 2021;50:1281-91.
