# Supplementary material for: Physiological reactions to acute stressors and subjective stress during daily life: A systematic review on ecological momentary assessment (EMA) studies
Source: PLoS One. 2022 Jul 27;17(7):e0271996. doi: 10.1371/journal.pone.0271996 (PMC9328558; doi:10.1371/journal.pone.0271996)
Supplement: S4 Table — (PDF) [file pone.0271996.s004.pdf]

S4 Table

Article title: Physiological reactions to acute stressors and subjective stress during daily life: A systematic review on ecological momentary assessment (EMA) studies

Authors: Jeannette Weber, Peter Angerer, Jennifer Apolinário-Hagen

Institute of Occupational-, Social- and Environmental Medicine, Centre for Health and Society, Medical Faculty, Heinrich-Heine-University Düsseldorf, Moorenstraße 5, 40225 Düsseldorf, Germany

S4 Table. Description of studies examining within-subject associations between acute stress and cardiovascular outcomes

Study	Study population and setting	Exposure variable (scale range[1])	Sampling schedule per participant	Max. time lag between exposure and outcome	Results (direct association between exposure and outcome)
**Balducci et al., 2021 (1)**	61 workers in Italy; M_{age}=47.5 (SD=12, range 23-68), female: 43%	Severity of acute stressors at work (scale range: 1-7)	10 days; daily	Same day	<u>SBP:</u> No association (WS: b=0.34, SE=0.48, p>.05)
**Bishop et al., 2003 (2)**	92 police patrol officers at work in Singapore; M_{age}=26.85 (SD=5.15) female: 0%	Severity of acute stressors at work: job demands (scale range: 1-4)	1 day; every 30 minutes	10 minutes	Moderation effect of job control was included in the following models:<br><u>SBP:</u> No association (BS: b=-.29 , SE=0.99, p>.05; WS: b=0.16, SE=0.16, p>.05)<br><u>DBP:</u> No association (BS: b=.10, SE=0.65, p>.05; WS: b=0.38, SE=0.31, p>.05)<br><u>HR:</u> No association (BS: b=-.06, SE=1.11, p>.05; WS: b=0.45, SE=0.31, p>.05).<br><u>Mean arterial pressure:</u> No association (BS: b=-.03, SE=0.69, p>.05; WS: b=0.33, SE=0.28, p>.05).<br><u>Pressure rate product:</u> No association (BS: b=-.04, SE=0.17, p>.05; WS: b=0.08, SE=0.05, p>.05).
**Bowen et al., 2014 (3)**	94 heterosexual couples (N=188) at workplace and in the evening at home with partner in the US;	Global subjective stress (scale range: 1-4)	1 day; every 30 minutes	concurrent	<u>SBP:</u> Positive association (p<.01)<br><u>DBP:</u> Positive association (p<.05)

	M_{age}=29.6 (SD=8.6), female: 50%				
**Brondolo et al., 2003 (4)**	104 participants in the US; M_{age}=30 (SD=7,7, range=18-46); female: 62%	Severity of acute stressors regarding social interactions (scale range: 1-5)	1 day; every 20 minutes	concurrent	SBP: Positive association (WS: b=1.97, SE=0.96, p<.05; BS: b=7.76, SE=4.94, p>.05),<br>DBP: Positive association (WS: b=2.28, SE=0.59, p<.001; BS: b=2.49, SE=2.86, p>.05)<br>HR: No association (WS: b=1.67, SE=0.89, p>.05; BS: b=-6.76, SE=3.83, p>.05)
**Buckley et al., 2004 (5)**	36 Vietnam veterans (19 with post-traumatic stress disorder (PTSD), 17 without PTSD) in the US; M_{age}=51.1 (SD=3.3) with PTSD; M_{age}=53.4 (SD=3.1) without PTSD; female: 0%	Global subjective stress (dichotomous scale: 0/1);	1 day; every 20 minutes	concurrent	Moderation effect of PTSD was included in the following models:<br>SBP: No association (WS: b=-2.99, SE=2.76, p>.05)<br>DBP: No association (WS: b=-1.48, SE=1.88, p>.05)<br>HR: No association (WS: b=-4.53, SE=2.55, p>.05)
**Carels et al., 2003 (6)**	45 participants with congestive heart failure in the US; M_{age}=66 (SD=11.7); female: 39%	Severity of acute stressors at work: job demands (scale range: 1-5)	1 day; exposure: 2 times/hour outcome: continuously (aggregated per hour)	1 hour	Frequency of premature ventricular contractions: No association (WS: b=1.1, SE=11.1, p=.92)<br>Frequency of repetitive premature ventricular contractions: No association (WS: b=0.2, SE=7.3, p=.982)<br>Tachycardia: No association (WS: b=1.6, SE=2.7, p=.544)
**Conley & Lehman, 2012 (7)**	99 undergraduate students in the US; M_{age}=21; female: 70%	Occurrence of acute and anticipated stressors in education (dichotomous scale: 0 = no stressor occurred, 1 = stressor occurred);	4 days exposure: daily outcome: hourly	1 hour	SBP: Positive association with acute stressors (WS: b=3.80, SE=1.21, p=.002). No association with anticipated stressors (WS: b=-0.53, SE=0.56, p=0.345);<br>DBP: No association with acute stressors (WS: b=1.29, SE=1.14, p=.259) or anticipated stressors (WS: b=0.28, SE=0.50, p=.581);<br>HR: No association with acute academic stressors (WS: b=2.32, SE=1.65, p=.163) or anticipated academic stressor (WS: b=-0.63, SE=0.68, p=.357)
**Dennis et al., 2016 (8)**	219 participants with PTSD and without PTSD in the US; M_{age}=30.32	Global subjective stress (scale range: 0-4);	1 day; exposure: not reported; outcome: continuously	5 minutes	HRV (low frequency domain): Negative association (WS: b=-1.14, SE=0.37, p<.01; BS: b=0.44, SE=1.24, p>.10)<br>HRV (High frequency domain): No association (WS: b=-0.57, SE=0.31, p<.10; BS: b=1.06, SE=1.02, p>.10)<br>HR: No association (WS: b=0.49, SE=0.30, p>.10; BS: b=-0.94, SE=1.01,

	(SD=5.42, range=18-39) with PTSD; M_{age}=27.8 (SD=5.47, range=18-39) without PTSD; female: 49% with PTSD; 53% without PTSD				p>.10)
**Enkelmann et al., 2005 (9)**	108 male police patrol officers at shift with patrol duties in Singapore; M_{age}= 26.9 (SD=5.7); female: 0%	Severity of acute stressors in social interactions	1 day; every 30 minutes	10 minutes	SBP/DBP: No main effects reported
**French, 2017 (10)**	100 full-time workers in the US; M_{age}= 33.09 (SD= 9.09); female: 63%	Occurrence of acute stressors regarding work-family conflict (momentary conflict - dichotomous scale: 0/1; accumulated conflict - sum of conflicts)	3 days; 8 times/day	2 hours	SBP: No association with momentary (ß=-1.40, p=.09) of daily accumulated (ß=0.18, p=.63) work-to-family conflict and momentary family-to-work conflict (ß=-0.11, p=.88), positive association with daily accumulated family-to-work conflict (ß=0.68, p<.01)<br>DBP: no association with momentary (ß=0.46, p=.05) and daily accumulated (ß=-0.01, p=.98) work-to-family conflict and momentary (ß=0.93, p=.15) and daily accumulated (ß=0.24, p=.24) family-to-work conflict<br>HR: No association with momentary (ß=0.69, p=.47) or daily accumulated (ß=-0.54, p=.26) work-to-family conflict. Negative association with momentary (ß=-1.97, p=.03) and daily accumulated (ß=-0.92, p<.01) family-to-work conflict
**Gaggioli, 2013 (11)**	6 participants in Italy; M_{age}=22; female: 50%	Severity of acute stressors: overall, in current activity and regarding social interactions (scale range: -0 - 3); global subjective stress (scale range: 1-10)	7 days; exposure: 5-6 times/day outcome: continuously	12 minutes	HR: HR measured between 4 to 0 minutes before, was positively correlated with severity of acute stressors (r=0.173, p=.032) and global subjective stress (r=0.205, p=.004). HR measured between 8 to 4 minutes before, was positively correlated with severity of acute stressors (r=0.175, p=.031) and global subjective stress (r=0.176, p=0.014). HR measured between 12 to 8 minutes before, was positively correlated global stress (r=0.168, p=0.021) but not with severity of acute stressors (r=0.151, p=.066). No significant correlation between HR and severity of acute stressors in current activity and social interactions.
**Gallo et al., 2006 (12)**	205 adolescents on school days in the US; Age Range 14-16; female: 50%	Severity of acute stressors regarding social interactions: conflicting social	2 days; every 30 minutes	10 minutes	Moderation effects of attachment were included in those models:<br>SBP: No association with pleasant/unpleasant interactions (b=0.23, SE=0.16, p>.10) and conflicting interactions (b=0.05, SE=0.19, p>.10)<br>DBP: Positive association with pleasant/unpleasant interaction (b=0.24, SE=0.10, p<.05). No direct association with conflicting interaction (b=0.13,

		interactions and pleasant/unpleasant social interactions (scale range: 1-6)			SE=0.12, p>.10)<br>HR: No association with pleasant/unpleasant interaction (b=0.30, SE=0.13, p<.10) or conflicting interaction (b=-0.02, SE=0.14, p>.10)
**Hawkley et al., 2003 (13)**	70 undergraduate students in the US; M_{age}=19.2 (SD=1.0); female: 50%	Severity of acute stressors in current activity (scale range: 1-5)	1 day; 9 times/day	concurrent	SBP, DBP, mean ambulatory blood pressure, HR, pre-ejection period, respiratory sinus arrhythmia or stroke volume: no association
**Ilies et al., 2010 (14)**	64 university employees in the US; M_{age}=42.58 (SD=9.44); female: 80%	Severity of acute stressors at work: job demands (scale range: 1-5)	10 days; 3 times/day (aggregated per day)	concurrent	SBP: Positive association (WS: b=2.48, ß=0.16, t=1.81, p<.05)
**Johnston et al., 2016 (15)**	100 Nurses at working shifts in medical and surgical wards in Scotland; M_{age}=36,4; female: 93%	Severity of acute stressors at work – job demands, work effort (dichotomous rating scale: 0/1)	2 days; exposure: every 90 minutes outcome: continuously	concurrent	HR: Positive association with demands (WS: ß=0.84, SE=0.11, p<.05; BS: ß=2.86, SE=1.11, p<.05) and effort (WS: ß=0.86, SE=0.14, p<.05; BS: ß=3.25, SE=1.40, p<.05).
**Kamarck et al., 2002 (16)**	340 older adults in the US; M_{age}= not reported; female: 51%	Severity of acute stressors at work: job demands and regarding social interactions (scale range: 1-4)	6 days; every 45 minutes	10 minutes	SBP: Positive association with job demands (WS: b=0.18, p<.05, BS: b=0.15, p<.01) and social interactions (WS: b=0.41, p<.05, BS: b=0.04, p>.05).<br>DBP: Positive association with job demands (WS: b=0.26, p<.0001, BS: b=0.12, p<.05) and social interactions (WS: b=0.17, p<.0001, BS: b=0.01, p>.05).
**Kamarck et al., 2018 (17); Thomas et al., 2019 (18)**	477 working midlife adults in the US; M_{age}=42.7 (SD=7.3, range=30-54); female: 52%	Occurrence of acute stressors at work: task strain - ratio between demands and control (dichotomous scale: 1 = high demands and low control, 0 = all other conditions) and regarding social	4 days; hourly	10 minutes	SBP: Positive association with task strain (WS: b=0.67,p=.003) and social interactions (WS: b=0.47, p=.003)<br>DBP: Positive association with task strain (WS: b=0.63, p<.001). No association with social interactions (WS: b=0.16, p=.16)

		interactions (scale range: 1-6)			
**Kamarck et al., 1998 (19)**	120 full time workers as part of the Pittsburgh study in the US; M_{age}=35 (range=23-50); female: 53%	Severity of acute stressors at work: job demands (dichotomous rating scale: 0/1) and regarding social interactions (scale range: 1-4)	6 days; every 45 minutes	10 minutes	SBP: No association with job demands (WS: b=-0.29, p=.06) or social interactions<br>DBP: No association with job demands or social interactions<br>HR: No association with social interactions. Positive association with job demands (WS: b=0.50, p<.05).
**Luecken et al., 2009 (20)**	91 undergraduate students (loss group experienced childhood parental death, control group did not experience childhood parental death) in the US; age range=18-29; female: 63%	Occurrence of any acute stressors (dichotomous scale: 0/1)	1 day; every 30 minutes	30 minutes	SBP: Positive association (WS: b=1.8, t(2179)=3.2, p=.001)<br>DBP: Positive association (WS: b=1.0, t(2247)=2.2, p=.031)
**Lumley et al., 2014 (21)**	40 female managers at work working in private or public sectors in Sweden; M_{age}=36.03 (SD=4.81); female: 100%	Global subjective stress (scale range: 1-6);	1 day; exposure: 3-14 times outcome: continuously	concurrent	HR: Positive association (WS: b=0.94, p.018)
**Määttänen et al., 2021 (22)**	44 students in Finland; M_{age}=25.0 (SD=5.4, range 20-47), female: 80%	Occurrence of any acute stressor (dichotomous scale)	3-5 days; exposure: every 45 minutes outome: continuously	concurrent	HRV – SDNN: No association (WS: p=0.09-0.44)
**Pieper et al., 2007**	73 teachers at secondary schools in the	Occurrence of any acute stressor	4 days; exposure: hourly	Pieper et al. 2007: 1 hour; Pieper et al.	Pieper et al., 2007 (23):<br>HR: Positive association (b=2.75, SE=0.77, CI=1.98-3.52, z=3.55, p<.001)

**(23)/ Pieper et al., 2010 (24)**	Netherlands; M_{age}=24.4 (SD=3.5); female: 33%	(dichotomous scale: 0/1)	outcome: continuously	2010: 15 minutes, 1 hour, 2 hours	RMSSD: No association (b=-0.05, SE=0.03,p=.098)<br>Pieper et al., 2010 (24):<br>HR: No association<br>RMSSD: No association
**Pollard et al., 2007 (25)**	33 university employees in non-manual jobs at working days in UK; M_{age}=43.1 (SD=9.8); female: 100%	Global subjective stress (scale range: 1-7)	2 days; 6 times/day	1 hour	SBP: Positive association (WS: b=1.39, t=3.65, p<.001)<br>DBP: Positive association (WS: b=0.88, t=2.93, p=.004)<br>HR: Positive association (WS: b=0.98, t=2.69, p=.008)
**Potter, 2019 (26)**	26 adults being overweight in the US; M_{age} = 27.51 (SD=9.83); female: 56%	Global subjective stress (scale range: 0-4)	7 days; exposure: 6 times/day outcome: continuously	concurrent	HR: No association (b=2.31-2.46, SE=4.37-4.39, p=.57-.60)
**Schilling et al., 2020 (27)**	173 police workers in Switzerland; M_{age} = 37.64 (SD=9.80); female: 34%	Global subjective stress (scale range: 1-5)	2 days; exposure: 8 times/day outcome: continuously	concurrent	Moderation effect of cardiorespiratory fitness was included in the model:<br>HRV - RMSSD: No association (WS: ß=-0.02, SE=0.03, p=0.437)
**Schmid & Thomas, 2020 (28)**	101 school teachers in Germany; M_{age} = 42.87 (SD=11.46); female: 69%	Severity of acute stressors at work (scale range: 1-7)	2 days; exposure: 6 times/day outcome: continuously	2 hours	HRV – RMSSD: No association (WS for emotional demands: b=0.00, SE=0.01, P=0.736; WS for time pressure: b=-0-02, SE=0.01, p=0.06)
**Schwerdtfeger & Dick, 2019 (29)**	43 male firefighters at work in Germany; M_{age}=32.7 (SD=6.9); female: 0%	Severity of acute stressors at work (dichotomous rating scale: 1 = baseline - 3 = high stressful emergency operations)	1 day; exposure: hourly outcome: continuously	1 hour	Moderation effects of resilience were included in those models:<br>HRV: lnRMSSD was negatively associated with stressful emergency-operations (b=-0.23, SE=0.08, 95% CI=-0.39 - -0.08). lnSDNN tended to be negatively associated with stressful emergency operations (b=-0.12, SE=0.06, 95%CI = -0.24 - 0.00).
**Shockley et al., 2013 (30)**	58 employees in the US; M_{age}=37.5; female: 90%	Occurrence of acute stressors regarding work-family conflict (dichotomous rating scale: 0/1)	10 days; exposure: daily outcome: 4 times/day	not reported (approximately 6 hours)	SBP: No association with work-family conflict (WS: ß=0.02, SE=0.01, p>.05), work-to-family conflict (WS: ß=0.01, SE=0.01, p>.05) or family-to-work conflict (WS: ß=0.01, SE=0.01, p>.05)<br>DBP: No association with work-family conflict (WS: ß=0.00, SE=0.02, p>.05), work-to-family conflict (WS: ß=-0.01, SE=0.01, p>.05) or family-to-work conflict (WS: ß=0.00, SE=0.02, p>.05))<br>HR: Positive association with work-family conflict (WS: ß=0.04, SE=0.02, p<.05) and family-to-work conflict (WS: ß=0.05, SE=0.02, p<.01). No

					association with work-to-family conflict (WS: ß=0.02, SE=0.02, p>.05)
**Simon et al., 2020 (31)**	174 adults in the US; M_{age} = 31.23 (SD=6.49, range 18-46); female: 50%	Global subjective stress (dichotomous scale: 0/1)	5 days; exposure: every 30 minutes outcome: continuously	not reported (approximately < 30 minutes)	HR: Positive association (WS: b=.93, SE=.19, p<.001) HRV-HF: Negative association (WS: b=-.02, SE=.01, p=.024) HRV-RMSSD: Negative association (WS: b=-.02, SE=.01, p=.006)
**Smith et al., 2007 (32)**	80 patients with acute myocardial infarction in the US; M_{age}= 57 (SD=11); female: 35%	Global subjective stress (scale range: 1-4)	1 day; exposure: every 30 minutes outcome: continuously (aggregated per hour)	concurrent	Number of ventricular premature beats: Positive association (WS: b=0.74, 95% CI=0.50-0.99 p<.001)
**Uchino et al., 2006 (33)**	214 middle-aged and older married couples in the US (N=428); M_{age}=52.8 (SD=10.0); female: 50%	Occurrence of any acute stressor (dichotomous rating scale: 0/1)	1 day; every 45 minutes	concurrent	SBP: No association DBP: Positive association (WS: b=1.71, p<.01)
**Wong & Kelloway, 2016 (34)**	55 care workers at work in Canada; M_{age}=43 (SD=9, range=25-62); female: 93%	Severity of acute stressors regarding social interactions (scale range: 1 - 5*)	1 day; hourly	15 minutes	SBP: Negative association (WS: b=-1.59, SE=0.68, 95% CI=-2.93- -0.24, p<.05)
**Wrzus et al., 2013 (35)**	89 participants in Germany; M_{age}=42.4 (SD=19.0); female: 55%	Acute stressors: circumscribed acute stressors = 1 life domain affected; complex acute stressor > 1 life domain affected (dichotomous scale: 0/1)	2 days; exposure: 6 times/day outcome: continuously	2 hours	Moderation effects of age were included in those models: RMSSD: No direct association with circumscribed (WS: b=-0.06, p>.05) and complex acute stressors (WS: b=-0.20, p>.05).

Notes. Abbreviations: b= unstandardized regression coefficient, ß = standardized regression coefficient, BS = between-subject effect, DBP = Diastolic blood pressure, HF = high frequency, HR = Heart rate, HRV = Heart rate variability, RMSSD = root mean square of the successive differences, SBP = Systolic blood pressure, SD = standard deviation, SDNN = standard deviation of NN intervals, SE = standard error, WS= Within-subject effect; [1] Higher values correspond to more stress. If higher values correspond to lower stress, those scale ranges will be marked by an asterisk; * higher values correspond to lower stress

References

1. Balducci C, Spagnoli P, Toderi S, Clark MA. A within-individual investigation on the relationship between day level workaholism and systolic blood pressure. WORK AND STRESS.

2. Bishop GD, Enkelmann HC, Tong EM, Why YP, Diong SM, Ang J, Khader M. Job demands, decisional control, and cardiovascular responses. J Occup Health Psychol. 2003;8:146-56.

3. Bowen KS, Uchino BN, Birmingham W, Carlisle M, Smith TW, Light KC. The stress-buffering effects of functional social support on ambulatory blood pressure. Health Psychol. 2014;33:1440-3.

4. Brondolo E, Rieppi R, Erickson SA, Bagiella E, Shapiro PA, McKinley P, Sloan RP. Hostility, interpersonal interactions, and ambulatory blood pressure. Psychosom Med. 2003;65:1003-11.

5. Buckley TC, Holohan D, Greif JL, Bedard M, Suvak M. Twenty-four-hour ambulatory assessment of heart rate and blood pressure in chronic PTSD and non-PTSD veterans. J Trauma Stress. 2004;17:163-71.

6. Carels RA, Cacciapaglia H, Perez-Benitez CI, Douglass O, Christie S, O'Brien WH. The association between emotional upset and cardiac arrhythmia during daily life. J Consult Clin Psychol. 2003;71:613-8.

7. Conley KM, Lehman BJ. Test anxiety and cardiovascular responses to daily academic stressors. Stress Health. 2012;28:41-50.

8. Dennis PA, Dedert EA, Van Voorhees EE, Watkins LL, Hayano J, Calhoun PS, Sherwood A, Dennis MF, Beckham JC. Examining the crux of autonomic dysfunction in posttraumatic stress disorder: Whether chronic or situational distress underlies elevated heart rate and attenuated heart rate variability. Psychosom Med. 2016;78:805-9.

9. Enkelmann HC, Bishop GD, Tong EM, Diong SM, Why YP, Khader M, Ang J. The relationship of hostility, negative affect and ethnicity to cardiovascular responses: an ambulatory study in Singapore. Int J Psychophysio. 2005;56:185-97.

10. French KA. Episodic work-family conflict and strain: A dynamic perspective (Doctoral dissertation, University of South Florida, Tampa, USA). 2017; Available from: https://scholarcommons.usf.edu/etd/7399/.

11. Gaggioli A, Pioggia G, Tartarisco G, Baldus G, Corda D, Cipresso P, Riva G. A mobile data collection platform for mental health research. Pers Ubiquitous Comput. 2013;17:241-51.

12. Gallo LC, Matthews KA. Adolescents' attachment orientation influences ambulatory blood pressure responses to everyday social interactions. Psychosom Med. 2006;68:253-61.

13. Hawkley LC, Burleson MH, Berntson GG, Cacioppo JT. Loneliness in everyday life: Cardiovascular activity, psychosocial context, and health behaviors. J Pers Soc Psychol. 2003;85:105-20.

14. Ilies R, Dimotakis N, De Pater IE. Psychological and physiological reactions to high workloads: Implications for well-being. Pers Psychol. 2010;63:407-36.

15. Johnston D, Bell C, Jones M, Farquharson B, Allan J, Schofield P, Ricketts I, Johnston M. Stressors, appraisal of stressors, experienced stress and cardiac response: A real-time, real-life investigation of work stress in nurses. Ann Behav Med. 2016;50:187-97.

16. Kamarck TW, Janicki DL, Shiffman S, Polk DE, Muldoon MF, Liebenauer LL, Schwartz JE. Psychosocial demands and ambulatory blood pressure: a field assessment approach. Physiol Behav. 2002;77:699-704.

17. Kamarck TW, Li X, Wright AG, Muldoon MF, Manuck SB. Ambulatory blood pressure reactivity as a moderator in the association between daily life psychosocial stress and carotid artery atherosclerosis. Psychosom Med. 2018;80:774-82.

18. Thomas MC, Kamarck TW, Li X, Erickson KI, Manuck SB. Physical activity moderates the effects of daily psychosocial stressors on ambulatory blood pressure. Health Psychol. 2019;38:925-35.

19. Kamarck TW, Shiffman SM, Smithline L, Goodie JL, Paty JA, Gnys M, Jong JY. Effects of task strain, social conflict, and emotional activation on ambulatory cardiovascular activity: daily life consequences of recurring stress in a multiethnic adult sample. Health Psychol. 1998;17:17-29.

20. Luecken LJ, Kraft A, Appelhans BM, Enders C. Emotional and cardiovascular sensitization to daily stress following childhood parental loss. Dev Psychol. 2009;45:296-302.

21. Lumley MA, Shi W, Wiholm C, Slatcher RB, Sandmark H, Wang S, Hytter A, Arnetz BB. The relationship of chronic and momentary work stress to cardiac reactivity in female managers: feasibility of a smart phone-assisted assessment system. Psychosom Med. 2014;76:512-8.

22. Määttänen I, Henttonen P, Valiaho J, Palomaki J, Thibault M, Kallio J, Mantyjarvi J, Harviainen T, Jokela M. Positive affect state is a good predictor of movement and stress: combining data from ESM/EMA, mobile HRV measurements and trait questionnaires. Heliyon. 2021;7:e06243.

23. Pieper S, Brosschot JF, van der Leeden R, Thayer JF. Cardiac effects of momentary assessed worry episodes and stressful events. Psychosom Med. 2007;69:901-9.

24. Pieper S, Brosschot JF, van der Leeden R, Thayer JF. Prolonged cardiac effects of momentary assessed stressful events and worry episodes. Psychosom Med. 2010;72:570-7.

25. Pollard TM, Pearce KL, Rousham EK, Schwartz JE. Do blood pressure and heart rate responses to perceived stress vary according to endogenous estrogen level in women? Am J Phys Anthropol. 2007;132:151-7.

26. Potter L. An ecological momentary investigation of weight stigma: Exploring associations between weight vigilance and poor health in everyday life (Doctoral dissertation, The Pennsylvania State University, Pennsylvania, USA). 2019; Available from: https://etda.libraries.psu.edu/catalog/15843lnc5003.

27. Schilling R, Herrmann C, Ludyga S, Colledge F, Brand S, Puhse U, Gerber M. Does Cardiorespiratory Fitness Buffer Stress Reactivity and Stress Recovery in Police Officers? A Real-Life Study. FRONTIERS IN PSYCHIATRY. 2020;11.

28. Schmid RF, Thomas J. Teachers' ambulatory heart rate variability as an outcome and moderating variable in the job demands-resources model. ANXIETY STRESS AND COPING. 2020;33:387-402.

29. Schwerdtfeger AR, Dick K. Episodes of momentary resilience in daily life are associated with HRV reductions to stressful operations in firefighters: An ambulatory assessment approach using bayesian multilevel modeling. J Posit Psychol. 2019;14:593-602.

30. Shockley KM, Allen TD. Episodic work-family conflict, cardiovascular indicators, and social support: An experience sampling approach. J Occup Health Psychol. 2013;18:262-75.

31. Simon SG, Sloan RP, Thayer JF, Jamner LD. Taking context to heart: Momentary emotions, menstrual cycle phase, and cardiac autonomic regulation. PSYCHOPHYSIOLOGY. 2021;58.

32. Smith PJ, Blumenthal JA, Babyak MA, Georgiades A, Sherwood A, Sketch MH, Jr., Watkins LL. Ventricular ectopy: impact of self-reported stress after myocardial infarction. Am Heart J. 2007;153:133-9.

33. Uchino BN, Berg CA, Smith TW, Pearce G, Skinner M. Age-related differences in ambulatory blood pressure during daily stress: evidence for greater blood pressure reactivity with age. Psychol Aging. 2006;21:231-9.

34. Wong JHK, Kelloway EK. What Happens at Work Stays at Work? Workplace Supervisory Social Interactions and Blood Pressure Outcomes. J Occup Health Psychol. 2016;21:133-41.

35. Wrzus C, Mueller V, Wagner GG, Lindenberger U, Riediger M. Affective and Cardiovascular Responding to Unpleasant Events From Adolescence to Old Age: Complexity of Events Matters. Dev Psychol. 2013;49:384-97.
