# Supplementary material for: Physiological reactions to acute stressors and subjective stress during daily life: A systematic review on ecological momentary assessment (EMA) studies
Source: PLoS One. 2022 Jul 27;17(7):e0271996. doi: 10.1371/journal.pone.0271996 (PMC9328558; doi:10.1371/journal.pone.0271996)
Supplement: S5 Table — (PDF) [file pone.0271996.s005.pdf]

S5 Table

Article title: Physiological reactions to acute stressors and subjective stress during daily life: A systematic review on ecological momentary assessment (EMA) studies

Authors: Jeannette Weber, Peter Angerer, Jennifer Apolinário-Hagen

Institute of Occupational-, Social- and Environmental Medicine, Centre for Health and Society, Medical Faculty, Heinrich-Heine-University Düsseldorf, Moorenstraße 5, 40225 Düsseldorf, Germany

S5 Table. Description of studies examining within-subject associations between acute stress and other outcomes

Study	Study population and setting	Exposure variable (scale range[1])	Sampling schedule per participant[2]	Max. time lag between exposure and outcome	Results (direct association between exposure and outcome)
**Benjamin, 2021 (1)**	44 adolescents with type I diabetes in the US; M_{age}= 15.4 (SD=1.05, range 13-17); female: 68%	Severity of acute general stressors (scale range: 1-5)<br>Severity of acute stressors regarding diabetes (scale range: 1-5)	7 days;<br>Exposure: daily<br>Outcome: >5 times/day	same day	Blood glucose: No association with severity of acute general stressors (WS: b=5.54, SE=5.86, p>.05; BS: b=18.76, SE=11.06, p>.05) and positive association with severity of acute stressors regarding diabetes (WS: b=9.44, SE=4.50, p<.05; BS: b=12.79, SE=10.07, p>.05)
**Berg et al., 2020 (2)**	199 patients with type I diabetes in the US; M_{age}= 46.81 (SD=13.95, range 25.9–74.9); female: 52%	Number of acute general stressors (scale range: 0-1)<br>Number of acute stressors regarding diabetes (scale range: 0-1)	14 days;<br>Exposure: daily<br>Outcome: dependent on regular measurement times	Same day	Blood glucose: No association with number of acute general stressors (WS: b=2.66, SE=5.68, p>.05; between-person: b=-26.83, SE=28.88, p>.05) and positive association with number of acute stressors regarding diabetes (WS: b=67.01, SE=6.28, p>.001; BS: b=82.09, SE=21.64, p>.001, in this model interactions with age and comorbidity were included)
**Birditt et al., 2018 (3)**	89 middle-aged to old adults in the US; M_{age}=67.72	Number of acute stressors regarding social interactions	5 days;<br>Exposure: daily<br>Outcome: 4 times/day	Overall levels per day, wake-evening slope: same day;	Overall salivary alpha-amylase levels per day[1]: No association at same day (WS: b=0.036, SE=0.04, p<.05) and previous day (WS: b=0.014, SE=0.04, p<.05)<br>Next-day awakening response of salivary alpha-amylase[1]: No association

	(SD=14.43, range 40-95); female: 55%	(scale range: 0-10)		Next-day awakening response: previous day	(WS: b=0.074, SE=0.10, p<.05)<br>Wake-evening slope of salivary alpha-amylase[1]: No association at same day (WS: b=-0.005, SE=0.00, p<.05) or previous day (WS: b=0.001, SE=0.00, p<.05)
**Clark et al., 1995 (4)**	72 persons with tension-type headaches and non-headache controls in the US; M_{age}=23.5 (range 18-36); female: 83%	Global subjective stress (scale range: 0-100)	3 days; every 30 minutes	30 minutes	Temporalis muscle activity: No significant correlation (p>.05)
**Geisser et al., 1995 (5)**	21 participants with chronic low back pain in the US; M_{age}=35.1 (range: 21-59); female: 57%	Global subjective stress (scale range: 1-8)	3 days; Exposure: every 30 minutes Outcome: continuously	concurrent	Electromyographic activity: No association (p>.05)
**Jelsma et al., 2021 (6)**	100 college students of racial and ethnic minorities in the US; M_{age}= 20.4 (SD=2.06, range 18-31); female: 61%	Occurrence of acute stressors regarding discrimination (dichotomous scale)	1 or 2 weeks; Exposure: 2 times/day Outcomes: continuously	concurrent	Electrodermal activity – sudomotor neuron activity: positive association (WS B=0.17, SE=0.45, p<.001)
**Liu et al., 2017/ Liu, 2017 (7, 8)**	165 family caregivers in the US; M_{age}= 61.99 (SD=10.70, range 39-89); female: 88%	Number of acute stressors in caregiving (scale range: 0-19); Number of acute stressors not related to caregiving (scale range: 0-8)	8 days; Exposure: daily Outcome: 5 times/day	Same day	Slope of salivary alpha-amylase levels between 30 minutes after wake-up and before lunch: Negative association with care-related stressors (WS: b=-0.36, SE=0.18, p=.048; BS: b=-0.31, SE=0.17, p>.05)<br>Slope of salivary alpha-amylase levels between before lunch and late afternoon: No association with care-related stressors (WS: b=-0.33, SE=0.24, p>.05; BS: b=-0.18, SE=0.18, p>.05)<br>Slope of salivary alpha-amylase levels between late afternoon and before bed: Positive association with care-related stressors (WS: b=0.17, SE=0.08, p=.03; BS: b=0.17, SE=0.06, p<.01)<br>Salivary alpha-amylase levels before lunch: No association with care-related stressors (WS: b=-0.39, SE=0.70, p>.05; BS: b=-0.90, SE=1.20, p>.05) or non-care-related stressors (WS: b=2.41, SE=2.57, p>.05; BS: b=-4.59, SE=7.30, p>.05)<br>Salivary alpha-amylase levels at late afternoon: Negative association with care-related stressors (WS: b=-1.95, SE=0.77, p<.05; BS: b=-1.60, SE=1.22, p>.05). No association with non-care-related stressors (WS: b=-1.49, SE=2.84, p>.05; BS: b=-7.24, SE=7.42, p>.05)<br>Salivary alpha-amylase levels before bed: No association with care-related stressors (WS: b=0.20, SE=0.84, p>.05; BS: b=0.48, SE=1.16, p>.05) or

					non-care-related stressors (WS: b=2.63, SE=3.09, p>.05; BS: b=-8.17, SE=7.07, p>.05)<br>AUCg curve of salivary alpha-amylase levels: No association with care-related stressors (WS: b=-0.35, SE=0.44, p>.05; BS: b=-0.31, SE=1.01, p>.05) or non-care-related stressors (WS: b=-0.29, SE=1.61, p>.05; BS: b=-6.93, SE=6.06, p>.05)
**Nater et al., 2007 (9)**	76 university students in Germany; M_{age}= 26.7 (SD=8.8, range 18-58); female: 58%	Global subjective stress (scale range: 1-5)	1 day; 15 times/day	1 hour	Current salivary alpha-amylase levels: No association (p>.05)
**Polenick et al., 2021 (10)**	93 middle-aged to old adults in the US; M_{age}=67.77 (SD=14.57, range 40-95)	Number of acute stressors regarding social interactions (scale range: 0-10)	5 days; exposure: daily outcome: 4 times/day	Overall levels, wake-evening slope: same day; Next-day awakening response: previous day	Overall DHEA-S levels per day: No association at same day (b=-0.027, SE=0.03, p>.05) or previous day (b=0.062, SE=0.03, p>.05)<br>Next-day awakening response of DHEA-S: No association (WS: b=0.111, SE=0.10, p>.05)<br>Wake-Evening Slope of DHEA-S: No association with same day (WS: b=0.002, SE=0.00, p>.05) or previous day (WS: b=0.001, SE=0.00, p>.05)
**Pollard et al., 1996 (11)**	104 participants in UK; Male: 49%, M_{age}= 38.0 (SD=7.53); female: 51%, M_{age}= 37.9 (SD=7.00)	Severity of acute stressors at work: job demands (scale range: 1-5)	3 days; daily	Same day	Urinary levels of adrenaline: No association in women. Positive association in men (b=0.01, p=0.04, F=4.03).
**Savla et al., 2013 (12)**	28 spouse care partners of individuals with mild cognitive impairment in the US; M_{age}= 72.9 (SD=6.82); female: 90%	Occurrence of any acute stressor in caregiving: Memory- and behavior related problems of spouse - restless behavior, mood disturbances, memory-related problems; Severity of acute stressors regarding social interactions (dichotomous scale: 0/1)	4 days; exposure: daily outcome: 5 times/day	Same day	Diurnal slope of salivary alpha-amylase: Restless behavior was associated with a steeper decline throughout the day (main effect: b=-1.50, SE=0.65, p<.05; interaction effect with daily decline of alpha-Amylase: b=-0.02, SE=0.06, p>.10). No association with mood-related problems (main effect: b=0.83, SE=0.53, p>.10; interaction effect with daily decline of alpha-Amylase: b=0.03, SE=0.06, p>.10) or memory-related problems (main effect: b=0.20, SE=0.45, p>.10; interaction effect with daily decline of alpha-Amylase: b=-0.03, SE=0.06, p>.10). Severity of acute stressors regarding social interactions was association with flatter decline of alpha-Amylase (main effect: b=0.67, SE=0.44, p>.10; interaction effect with daily decline of alpha-Amylase: b=-0.12, SE=0.05, <.05).
**Stoffel**	60 working adults	Severity of acute	4 days; 6	Current levels:	Current salivary alpha-amylase: No association with severity of acute

**et al., 2021 (13)**	in Germany; M_{age}= 36.172 (SD=11.611, range 19-60); female: 33%	stressors regarding social interactions (scale range: 1-100*) Global subjective stress (scale range: 1-100)	times/day	5.5 hour; average levels: same day	stressors regarding social interactions (WS: b=-0.003, SE= 0.002, p = 0.070, BS: b=0.019, SE= 0.010, p=0.061) or global subjective stress (WS: p>.05) <u>Average salivary alpha-amylase levels/day:</u> No association with severity of acute stressors regarding social interactions (WS: b=0.002, SE=0.003, p=0.488) or global subjective stress (p>.05)
**Timmons et al., 2019 (14)**	218 participants in partnership at home in the US; M_{age}= 23.1 (SD=3.0, range: 18-25); female: 50%	Severity of acute stressors regarding social interactions (scale range: 0-10)	1 day; Exposure: hourly Outcome: continuously	concurrent	<u>Electrodermal activity:</u> No association (WS: b=-0.02, p=.15)

Notes. Abbreviations: b= unstandardized regression coefficient, ß = standardized regression coefficient, BS = between-subject effect, DHEA-S = Dehydroepiandrosterone sulfate, SD = standard deviation, SE = standard error, WS= Within-subject effect; [1] Higher values correspond to more stress. If higher values correspond to lower stress, those scale ranges will be marked by an asterisk; * higher values correspond to lower stress; [1] salivary alpha-amylase levels (U/ml) were log-transformed

References

1. Benjamin NE. Ecological momentary assessment of anxiety, daily stress, and daily glycemic control in adolescents with type 1 diabetes: Dissertation Abstracts International: Section B: The Sciences and Engineering. Vol.82(8-B),2021, pp. No Pagination Specified.; 2021.

2. Berg CA, Helgeson VS, Kelly CS, Tracy EL, Litchman ML, Butner JE. Age Differences in Reactivity to Daily General and Type 1 Diabetes Stressors. PSYCHOLOGY AND AGING. 2020;35:1115-26.

3. Birditt KS, Tighe LA, Nevitt MR, Zarit SH. Daily social interactions and the biological stress response: Are there age differences in links between social interactions and alpha-amylase? Gerontologist. 2018;58:1114-25.

4. Clark G, Sakai S, Merrill R, Flack V, McCreary C. Cross-correlation between stress, pain, physical activity, and temporalis muscle EMG in tension-type headache. Cephalalgia. 1995;15:511-8.

5. Geisser ME, Robinson ME, Richardson C. A time series analysis of the relationship between ambulatory EMG, pain, and stress in chronic low back pain. Biofeedback Self Regul. 1995;20:339-55.

6. Jelsma EB, Goosby BJ, Cheadle JE. Do trait psychological characteristics moderate sympathetic arousal to racial discrimination exposure in a natural setting? [References]: Psychophysiology. Vol.58(4), 2021, ArtID e13763.; 2021.

7. Liu Y, Granger DA, Kim K, Klein LC, Almeida DM, Zarit SH. Diurnal salivary alpha-amylase dynamics among dementia family caregivers. Health Psychol. 2017;36:160-8.

8. Liu Y. Aging, caregiving, health and well-being (Doctoral dissertation, The Pennsylvania State University, Pennsylvania, USA). 2017; Available from: https://etda.libraries.psu.edu/files/final_submissions/11686.

9. Nater UM, Rohleder N, Schlotz W, Ehlert U, Kirschbaum C. Determinants of the diurnal course of salivary alpha-amylase. Psychoneuroendocrinology. 2007;32:392-401.

10. Polenick CA, Birditt KS, Turkelson A, Perbix EA, Salwi SM, Zarit SH. Daily Social Interactions and HPA Axis Activity Among Midlife and Older Adults. Gerontologist. 2021;61:897-906.

11. Pollard TM, Ungpakorn G, Harrison GA, Parkes KR. Epinephrine and cortisol responses to work: A test of the models of Frankenhaeuser and Karasek. Ann Behav Med. 1996;18:229-37.

12. Savla J, Granger DA, Roberto KA, Davey A, Blieszner R, Gwazdauskas F. Cortisol, alpha amylase, and daily stressors in spouses of persons with mild cognitive impairment. Psychol Aging. 2013;28:666-79.

13. Stoffel M, Abbruzzese E, Rahn S, Bossmann U, Moessner M, Ditzen B. Covariation of psychobiological stress regulation with valence and quantity of social interactions in everyday life: disentangling intra- and interindividual sources of variation. JOURNAL OF NEURAL TRANSMISSION. 2021;128:1381-95.

14. Timmons AC, Han SC, Chaspari T, Kim Y, Pettit C, Narayanan S, Margolin G. Family-of-origin aggression, dating aggression, and physiological stress reactivity in daily life. Physiol Behav. 2019;206:85-92.
