# Supplementary material for: Physiological reactions to acute stressors and subjective stress during daily life: A systematic review on ecological momentary assessment (EMA) studies
Source: PLoS One. 2022 Jul 27;17(7):e0271996. doi: 10.1371/journal.pone.0271996 (PMC9328558; doi:10.1371/journal.pone.0271996)
Supplement: S6 Table — (PDF) [file pone.0271996.s006.pdf]

S6 Table

Article title: Physiological reactions to acute stressors and subjective stress during daily life: A systematic review on ecological momentary assessment (EMA) studies

Authors: Jeannette Weber, Peter Angerer, Jennifer Apolinário-Hagen

Institute of Occupational-, Social- and Environmental Medicine, Centre for Health and Society, Medical Faculty, Heinrich-Heine-University Düsseldorf, Moorenstraße 5, 40225 Düsseldorf, Germany

S6 Table Quality assessment

Study	Measurement frequency: at least 3 times per day	Compliance	Recall bias	Confounding	Validity and reliability of exposure
Almeida et al. 2016 [1]	no	NA	moderate	moderate	partly
Bai et al. 2017 [2]	no	yes	moderate	low to moderate	partly
Balducci et al. 2021 [3]	no	yes	high	moderate	yes
Barker et al. 2012 [4]	no	NA	moderate	moderate	no to partly
Benjamin 2021 [5]	no	yes	moderate to high	moderate	no to partly
Berg et al. 2020	no	yes	moderate to high	low	no to partly
Bernstein et al. 2018 [6]	yes	yes	low	moderate	no
Birditt et al. 2015 [7]	no	yes	moderate	moderate	NA
Birditt et al. 2016 [8]	no	yes	moderate	low to moderate	NA
Birditt et al. 2017 [9]	no	yes	moderate	low	NA
Birditt et al. 2018 [10]	no	yes	moderate	low to moderate	NA
Bishop et al. 2003 [11]	yes	no	low	low	partly
Bowen et al. 2014 [12]	yes	NA	low	low to moderate	NA
Brondolo et al. 2003 [13]	yes	yes	moderate	low to moderate	partly
Buckley et al. 2004 [14]	yes	NA	low to moderate	low	no
Carels et al. 2003 [15]	yes	NA	low to moderate	moderate	yes
Clark et al. 1995 [16]	yes	NA	moderate	high	no
Collip et al. 2011 [17]	yes	no	high	low to moderate	no
Conley & Lehman 2012 [18]	partly	NA	moderate to high	moderate	partly
Costanzo et al. 2012 [19]	no	NA	moderate	moderate	no
Crockett & Neff 2013 [20]	no	NA	moderate to high	low	no
Damaske et al. 2016 [21]	yes	yes	low	moderate	partly to yes
Dennis et al. 2016 [22]	NA	NA	moderate	moderate	no
Enkelmann et al. 2005 [23]	yes	no	low	low	partly
Fischer et al. 2016 [24]	yes	yes	low	moderate	no
French 2017 [25]	yes	yes	moderate	moderate	no
Gaggioli et al. 2013 [26]	yes	yes	low	high	no to partly
Gallo et al. 2006 [27]	yes	NA	low to moderate	low	no
Gartland et al. 2014 [28]	No	NA	moderate to high	low	partly
Geisser et al. 1995 [29]	yes	NA	moderate	high	no

Han et al. 2018 [30]	No	NA	moderate	moderate	No to partly
Hanson et al. 2000 [31]	yes	yes	moderate	moderate	partly
Hanson & Chen 2010 [32]	No	yes	moderate	moderate	no to partly
Hartley et al. 2019 [33]	No	NA	moderate	moderate	no
Hartley et al. 2012 [34]	No	NA	moderate	moderate	no to partly
Havermans et al. 2011 [35]	yes	yes	high	low to moderate	no
Hawkley et al. 2003 [36]	yes	yes	moderate	low	no
Heissel et al. 2018 [37]	No	NA	low	moderate	partly to yes
Hoppmann et al. 2006 [38]	yes	yes	moderate to high	low to moderate	NA
Ilies et al. 2010 [39]	Yes[1]	no	low	moderate	yes
Jacobs et al. 2007 [40]	yes	no	moderate	low to moderate	no
Jelsma., 2021 [41]	no	yes	moderate	moderate	partly
Johnson et al. 2021 [42]	yes	yes	low	low to moderate	no to partly
Johnston et al. 2016 [43]	yes	yes	low	moderate	yes
Kalpakjian et al. 2009 [44]	yes	yes	moderate	moderate	no
Kamarck et al. 2002 [45]	yes	yes	low	low	yes
Kamarck et al. 2018/Thomas et al. 2019 [46,47]	yes	NA	low	low	yes
Kamarck et al. 1998 [48]	yes	yes	low	low to moderate	yes
Keneski et al. 2018 [49]	No	NA	moderate to high	low	no
Lazarides et al. 2020	yes	yes	low	moderate	no to partly
Linz et al. 2018 [50]	yes	yes	moderate	low to moderate	no
Lippold et al. 2016 [51]	No	yes	moderate	moderate	no to partly
Lippold et al. 2016 [52]	No	yes	moderate	moderate	no
Liu et al. 2017/Liu 2017 [53,54]	No	yes	moderate	moderate	partly
Liu et al. 2018/ Liu 2017 [54,55]	No	yes	moderate	moderate	partly
Lee et al. 2019 [56]	No	NA	moderate	low to moderate	partly
Luecken et al. 2009 [57]	yes	yes	low	low to moderate	no
Lumley et al. 2014 [58]	yes	yes	low	low to moderate	no
Määttänen et al. 2021 [59]	yes	NA	moderate	moderate	no
Nater et al. 2007 [60]	yes	NA	moderate to high	low	no
O'Connor et al. 2020 [61]	No	yes	moderate	low	no to partly
Peeters et al. 2003 [62]	yes	no	high	low to moderate	no
Pieper et al. 2007/2010 [63,64]	yes	NA	moderate	moderate	no
Poleninck et al. 2021 [65]	no	yes	moderate	low	NA
Pollard et al. 1996 [66]	No	NA	high	moderate	yes
Pollard et al. 2007 [67]	yes	yes	high	low to moderate	no
Potter 2019 [68]	yes	no	low	moderate	no
Proulx 2015 [69]	No	NA	high	moderate	No to partly
Qu et al. 2020 [70]	no	NA	high	moderate	no
Savla et al. 2013 [71]	No	yes	moderate	moderate	partly to yes
Savla et al. 2011 [72]	No	yes	moderate	moderate	partly to yes
Savla et al. 2018 [73]	No	NA	moderate	moderate	No to partly
Saxbe et al. 2008 [74]	No	NA	high	moderate	yes
Schilling et al. 2020 [75]	yes	yes	low	moderate	no
Schlotz et al. 2006 [76]	yes	yes	moderate	moderate	no
Schmid & Thomas, 2020 [77]	yes	yes	moderate	low to moderate	no to partly
Schwerdtfeger & Dick 2019 [78]	yes	NA	low to moderate	moderate	no
Seaton et al. 2021 [79]	no	yes	moderate to high	moderate	yes
Seltzer et al. 2010 [80]	No	NA	moderate	moderate	yes
Shockley et al. 2013 [81]	yes	yes	high	moderate	no to partly
Simon et al. 2020 [82]	yes	yes	low	low to moderate	no
Sladek et al. 2019 [83]	yes	yes	moderate	low to moderate	no
Sladek et al. 2016 [84]	yes	yes	high	low to moderate	partly
Sladek et al. 2020 [85]	No	yes	moderate	low to moderate	no
Slatcher et al. 2010 [86]	No	NA	high	moderate	partly

Smith et al. 2007 [87]	yes	NA	moderate	moderate	no
Smyth et al. 1998 [88]	yes	yes	low to moderate	moderate	no
Smyth et al. 2017 [89]	yes	yes	moderate	low to moderate	no
Stawski et al. 2013 [90]	No	yes	moderate	moderate	No to partly
Stoffel et al. 2021 [91]	yes	yes	low to moderate	low	no
Timmons et al 2019 [92]	yes	yes	moderate	low to moderate	no
Uchino et al. 2006 [93]	yes	NA	moderate	low to moderate	no
Vaessen et al. 2018 [94]	yes	NA	low to moderate	moderate	no
van Eck et al. 1996 [95]	yes	yes	high	low to moderate	partly
van der Linden et al. 2021	yes	no	low	moderate	partly
van Duin et al. 2019 [96]	yes	no	low	low to moderate	no
Volmer & Fritsche 2016 [97]	yes[1]	NA	moderate	low to moderate	no
Wong et al. 2014 [98]	No	NA	moderate	moderate	No to partly
Wong et al. 2012 [99]	No	yes	moderate	moderate	No to partly
Wong & Shobo 2016 [100]	No	NA	moderate	moderate	No to partly
Wong & Shobo 2017 [101]	No	NA	moderate	moderate	No to partly
Wong & Kelloway 2016 [102]	yes	NA	moderate	low to moderate	no
Wright et al. 2021 [103]	yes	NA	low	moderate	no
Wrzus et al. 2013 [104]	yes	no	moderate	moderate	no

Notes: Abbreviation: NA = not available; [1] data was subsequently aggregated per day

References

1. Almeida DM, Davis KD, Lee S, Lawson KM, Walter K, et al. (2016) Supervisor Support Buffers Daily Psychological and Physiological Reactivity to Work-to-Family Conflict. J Marriage Fam 78: 165-179.
2. Bai S, Robles TF, Reynolds BM, Repetti RL (2017) Children's diurnal cortisol responses to negative events at school and home. Psychoneuroendocrinology 83: 150-158.
3. Balducci C, Spagnoli P, Toderi S, Clark MA A within-individual investigation on the relationship between day level workaholism and systolic blood pressure. WORK AND STRESS.
4. Barker ET, Greenberg JS, Seltzer MM, Almeida DM (2012) Daily Stress and Cortisol Patterns in Parents of Adult Children With a Serious Mental Illness. Health Psychol 31: 130-134.
5. Benjamin NE (2021) Ecological momentary assessment of anxiety, daily stress, and daily glycemic control in adolescents with type 1 diabetes: Dissertation Abstracts International: Section B: The Sciences and Engineering. Vol.82(8-B),2021, pp. No Pagination Specified.
6. Bernstein MJ, Zawadzki MJ, Juth V, Benfield JA, Smyth JM (2018) Social interactions in daily life: Within-person associations between momentary social experiences and psychological and physical health indicators. J Soc Pers Relat 35: 372-394.
7. Birditt KS, Nevitt MR, Almeida DM (2015) Daily interpersonal coping strategies: Implications for self-reported well-being and cortisol. J Soc Pers Relat 32: 687-706.

8. Birditt KS, Kim K, Zarit SH, Fingerman KL, Loving TJ (2016) Daily interactions in the parent-adult child tie: Links between children's problems and parents' diurnal cortisol rhythms. Psychoneuroendocrinology 63: 208-216.

9. Birditt KS, Manalel JA, Kim K, Zarit SH, Fingerman KL (2017) Daily interactions with aging parents and adult children: Associations with negative affect and diurnal cortisol. J Fam Psychol 31: 699-709.

10. Birditt KS, Tighe LA, Nevitt MR, Zarit SH (2018) Daily social interactions and the biological stress response: Are there age differences in links between social interactions and alpha-amylase? Gerontologist 58: 1114-1125.

11. Bishop GD, Enkelmann HC, Tong EM, Why YP, Diong SM, et al. (2003) Job demands, decisional control, and cardiovascular responses. J Occup Health Psychol 8: 146-156.

12. Bowen KS, Uchino BN, Birmingham W, Carlisle M, Smith TW, et al. (2014) The stress-buffering effects of functional social support on ambulatory blood pressure. Health Psychol 33: 1440-1443.

13. Brondolo E, Rieppi R, Erickson SA, Bagiella E, Shapiro PA, et al. (2003) Hostility, interpersonal interactions, and ambulatory blood pressure. Psychosom Med 65: 1003-1011.

14. Buckley TC, Holohan D, Greif JL, Bedard M, Suvak M (2004) Twenty-four-hour ambulatory assessment of heart rate and blood pressure in chronic PTSD and non-PTSD veterans. J Trauma Stress 17: 163-171.

15. Carels RA, Cacciapaglia H, Perez-Benitez CI, Douglass O, Christie S, et al. (2003) The association between emotional upset and cardiac arrhythmia during daily life. J Consult Clin Psychol 71: 613-618.

16. Clark G, Sakai S, Merrill R, Flack V, McCreary C (1995) Cross-correlation between stress, pain, physical activity, and temporalis muscle EMG in tension-type headache. Cephalalgia 15: 511-518.

17. Collip D, Nicolson N, Lardinois M, Lataster T, van Os J, et al. (2011) Daily cortisol, stress reactivity and psychotic experiences in individuals at above average genetic risk for psychosis. Psychol Med 41: 2305-2315.

18. Conley KM, Lehman BJ (2012) Test anxiety and cardiovascular responses to daily academic stressors. Stress Health 28: 41-50.

19. Costanzo ES, Stawski RS, Ryff CD, Coe CL, Almeida DM (2012) Cancer survivors' responses to daily stressors: Implications for quality of life. Health Psychol 31: 360-370.

20. Crockett EE, Neff LA (2013) When receiving help hurts: Gender differences in diurnal cortisol responses to spousal support. Soc Psychol Personal Sci 4: 190-197.

21. Damaske S, Zawadzki MJ, Smyth JM (2016) Stress at work: Differential experiences of high versus low SES workers. Soc Sci Med 156: 125-133.

22. Dennis PA, Dedert EA, Van Voorhees EE, Watkins LL, Hayano J, et al. (2016) Examining the crux of autonomic dysfunction in posttraumatic stress disorder: Whether chronic or situational distress underlies elevated heart rate and attenuated heart rate variability. Psychosom Med 78: 805-809.

23. Enkelmann HC, Bishop GD, Tong EM, Diong SM, Why YP, et al. (2005) The relationship of hostility, negative affect and ethnicity to cardiovascular responses: an ambulatory study in Singapore. Int J Psychophysio 56: 185-197.

24. Fischer S, Doerr JM, Strahler J, Mewes R, Thieme K, et al. (2016) Stress exacerbates pain in the everyday lives of women with fibromyalgia syndrome--The role of cortisol and alpha-amylase. Psychoneuroendocrinology 63: 68-77.

25. French KA (2017) Episodic work-family conflict and strain: A dynamic perspective (Doctoral dissertation, University of South Florida, Tampa, USA).

26. Gaggioli A, Pioggia G, Tartarisco G, Baldus G, Corda D, et al. (2013) A mobile data collection platform for mental health research. Pers Ubiquitous Comput 17: 241-251.

27. Gallo LC, Matthews KA (2006) Adolescents' attachment orientation influences ambulatory blood pressure responses to everyday social interactions. Psychosom Med 68: 253-261.

28. Gartland N, O'Connor DB, Lawton R, Bristow M (2014) Exploring day-to-day dynamics of daily stressor appraisals, physical symptoms and the cortisol awakening response. Psychoneuroendocrinology 50: 130-138.

29. Geisser ME, Robinson ME, Richardson C (1995) A time series analysis of the relationship between ambulatory EMG, pain, and stress in chronic low back pain. Biofeedback Self Regul 20: 339-355.

30. Han SH, Kim K, Burr JA (2018) Stress-buffering effects of volunteering on salivary cortisol: Results from a daily diary study. Soc Sci Med 201: 120-126.

31. Hanson EK, Maas CJ, Meijman TF, Godaert GL (2000) Cortisol secretion throughout the day, perceptions of the work environment, and negative affect. Ann Behav Med 22: 316-324.

32. Hanson MD, Chen E (2010) Daily stress, cortisol, and sleep: The moderating role of childhood psychosocial environments. Health Psychol 29: 394-402.

33. Hartley SL, DaWalt LS, Hong J, Greenberg JS, Mailick MR (2019) Positive Emotional Support in Premutation Carrier Mothers of Adolescents and Adults With Fragile X Syndrome: Gene by Environment Interactions. Am J Intellect Dev Disabil 124: 411-426.

34. Hartley SL, Seltzer MM, Hong J, Greenberg JS, Smith L, et al. (2012) Cortisol response to behavior problems in FMR1 premutation mothers of adolescents and adults with fragile X syndrome: A diathesis-stress model. Int J Behav Dev 36: 53-61.

35. Havermans R, Nicolson NA, Berkhof J, deVries MW (2011) Patterns of salivary cortisol secretion and responses to daily events in patients with remitted bipolar disorder. Psychoneuroendocrinology 36: 258-265.

36. Hawkley LC, Burleson MH, Berntson GG, Cacioppo JT (2003) Loneliness in everyday life: Cardiovascular activity, psychosocial context, and health behaviors. J Pers Soc Psychol 85: 105-120.

37. Heissel JA, Sharkey PT, Torrats-Espinosa G, Grant K, Adam EK (2018) Violence and Vigilance: The Acute Effects of Community Violent Crime on Sleep and Cortisol. Child Dev 89: e323-e331.

38. Hoppmann CA, Klumb PL (2006) Daily goal pursuits predict cortisol secretion and mood states in employed parents with preschool children. Psychosom Med 68: 887-894.

39. Ilies R, Dimotakis N, De Pater IE (2010) Psychological and physiological reactions to high workloads: Implications for well-being. Pers Psychol 63: 407-436.

40. Jacobs N, Myin-Germeys I, Derom C, Delespaul P, van Os J, et al. (2007) A momentary assessment study of the relationship between affective and adrenocortical stress responses in daily life. Biol Psychol 74: 60-66.

41. Jelsma EB, Goosby BJ, Cheadle JE (2021) Do trait psychological characteristics moderate sympathetic arousal to racial discrimination exposure in a natural setting? [References]: Psychophysiology. Vol.58(4), 2021, ArtID e13763.

42. Johnson JA, Zawadzki MJ, Jones DR, Reichenberger J, Smyth JM (2021) Intra-individual Associations of Perceived Stress, Affective Valence, and Affective Arousal with Momentary Cortisol in a Sample of Working Adults. Annals of Behavioral Medicine 22: 22.

43. Johnston D, Bell C, Jones M, Farquharson B, Allan J, et al. (2016) Stressors, appraisal of stressors, experienced stress and cardiac response: A real-time, real-life investigation of work stress in nurses. Ann Behav Med 50: 187-197.

44. Kalpakjian CZ, Farrell DJ, Albright KJ, Chiodo A, Young EA (2009) Association of daily stressors and salivary cortisol in spinal cord injury. Rehabil Psychol 54: 288-298.

45. Kamarck TW, Janicki DL, Shiffman S, Polk DE, Muldoon MF, et al. (2002) Psychosocial demands and ambulatory blood pressure: a field assessment approach. Physiol Behav 77: 699-704.

46. Thomas MC, Kamarck TW, Li X, Erickson KI, Manuck SB (2019) Physical activity moderates the effects of daily psychosocial stressors on ambulatory blood pressure. Health Psychol 38: 925-935.

47. Kamarck TW, Li X, Wright AG, Muldoon MF, Manuck SB (2018) Ambulatory blood pressure reactivity as a moderator in the association between daily life psychosocial stress and carotid artery atherosclerosis. Psychosom Med 80: 774-782.

48. Kamarck TW, Shiffman SM, Smithline L, Goodie JL, Paty JA, et al. (1998) Effects of task strain, social conflict, and emotional activation on ambulatory cardiovascular activity:

daily life consequences of recurring stress in a multiethnic adult sample. Health Psychol 17: 17-29.

49. Keneski E, Neff LA, Loving TJ (2018) The Importance of a Few Good Friends: Perceived Network Support Moderates the Association Between Daily Marital Conflict and Diurnal Cortisol. Soc Psychol Personal Sci 9: 962-971.

50. Linz R, Singer T, Engert V (2018) Interactions of momentary thought content and subjective stress predict cortisol fluctuations in a daily life experience sampling study. Sci Rep 8: 15462.

51. Lippold MA, Davis KD, McHale SM, Buxton OM, Almeida DM (2016) Daily stressor reactivity during adolescence: The buffering role of parental warmth. Health Psychol 35: 1027-1035.

52. Lippold MA, McHale SM, Davis KD, Almeida DM, King RB (2016) Experiences With Parents and Youth Physical Health Symptoms and Cortisol: A Daily Diary Investigation. J Res Adolesc 26: 226-240.

53. Liu Y, Granger DA, Kim K, Klein LC, Almeida DM, et al. (2017) Diurnal salivary alpha-amylase dynamics among dementia family caregivers. Health Psychol 36: 160-168.

54. Liu Y (2017) Aging, caregiving, health and well-being (Doctoral dissertation, The Pennsylvania State University, Pennsylvania, USA).

55. Liu Y, Almeida DM, Rovine MJ, Zarit SH (2018) Modeling cortisol daily rhythms of family caregivers of individuals with dementia: Daily stressors and adult day services use. J Gerontol B Psychol Sci Soc Sci 73: 457-467.

56. Lee HY, Jamieson JP, Miu AS, Josephs RA, Yeager DS (2019) An entity theory of intelligence predicts higher cortisol levels when high school grades are declining. Child Dev 90: e849-e867.

57. Luecken LJ, Kraft A, Appelhans BM, Enders C (2009) Emotional and cardiovascular sensitization to daily stress following childhood parental loss. Dev Psychol 45: 296-302.

58. Lumley MA, Shi W, Wiholm C, Slatcher RB, Sandmark H, et al. (2014) The relationship of chronic and momentary work stress to cardiac reactivity in female managers: feasibility of a smart phone-assisted assessment system. Psychosom Med 76: 512-518.

59. Määttänen I, Henttonen P, Valiaho J, Palomaki J, Thibault M, et al. (2021) Positive affect state is a good predictor of movement and stress: combining data from ESM/EMA, mobile HRV measurements and trait questionnaires. Heliyon 7: e06243.

60. Nater UM, Rohleder N, Schlotz W, Ehlert U, Kirschbaum C (2007) Determinants of the diurnal course of salivary alpha-amylase. Psychoneuroendocrinology 32: 392-401.

61. O'Connor DB, Branley-Bell D, Green JA, Ferguson E, O'Carroll RE, et al. (2020) Effects of childhood trauma, daily stress, and emotions on daily cortisol levels in individuals vulnerable to suicide. J Abnorm Psychol 129: 92-107.

62. Peeters F, Nicholson NA, Berkhof J (2003) Cortisol Responses to Daily Events in Major Depressive Disorder. Psychosom Med 65: 836-841.

63. Pieper S, Brosschot JF, van der Leeden R, Thayer JF (2007) Cardiac effects of momentary assessed worry episodes and stressful events. Psychosom Med 69: 901-909.

64. Pieper S, Brosschot JF, van der Leeden R, Thayer JF (2010) Prolonged cardiac effects of momentary assessed stressful events and worry episodes. Psychosom Med 72: 570-577.

65. Polenick CA, Birditt KS, Turkelson A, Perbix EA, Salwi SM, et al. (2021) Daily Social Interactions and HPA Axis Activity Among Midlife and Older Adults. Gerontologist 61: 897-906.

66. Pollard TM, Ungpakorn G, Harrison GA, Parkes KR (1996) Epinephrine and cortisol responses to work: A test of the models of Frankenhaeuser and Karasek. Ann Behav Med 18: 229-237.

67. Pollard TM, Pearce KL, Rousham EK, Schwartz JE (2007) Do blood pressure and heart rate responses to perceived stress vary according to endogenous estrogen level in women? Am J Phys Anthropol 132: 151-157.

68. Potter L (2019) An ecological momentary investigation of weight stigma: Exploring associations between weight vigilance and poor health in everyday life (Doctoral dissertation, The Pennsylvania State University, Pennsylvania, USA).

69. Proulx JA (2015) Are There Gender Differences in Stress Response in Older Adults? : Findings from the Normative Aging Study (Doctoral dissertation, Oregon State University, Oregon, USA).

70. Qu Y, Yang B, Telzer EH (2020) The Cost of Academic Focus: Daily School Problems and Biopsychological Adjustment in Chinese American Families. JOURNAL OF YOUTH AND ADOLESCENCE 49: 1631-1644.

71. Savla J, Granger DA, Roberto KA, Davey A, Blieszner R, et al. (2013) Cortisol, alpha amylase, and daily stressors in spouses of persons with mild cognitive impairment. Psychol Aging 28: 666-679.

72. Savla J, Roberto KA, Blieszner R, Cox M, Gwazdauskas F (2011) Effects of daily stressors on the psychological and biological well-being of spouses of persons with mild cognitive impairment. J Gerontol B Psychol Sci Soc Sci 66: 653-664.

73. Savla J, Zarit SH, Almeida DM (2018) Routine support to parents and stressors in everyday domains: Associations with negative affect and cortisol. J Gerontol B Psychol Sci Soc Sci 73: 437-446.

74. Saxbe DE, Repetti RL, Nishina A (2008) Marital satisfaction, recovery from work, and diurnal cortisol among men and women. Health Psychol 27: 15-25.

75. Schilling R, Herrmann C, Ludyga S, Colledge F, Brand S, et al. (2020) Does Cardiorespiratory Fitness Buffer Stress Reactivity and Stress Recovery in Police Officers? A Real-Life Study. FRONTIERS IN PSYCHIATRY 11.

76. Schlotz W, Schulz P, Hellhammer J, Stone AA, Hellhammer DH (2006) Trait anxiety moderates the impact of performance pressure on salivary cortisol in everyday life. Psychoneuroendocrinology 31: 459-472.

77. Schmid RF, Thomas J (2020) Teachers' ambulatory heart rate variability as an outcome and moderating variable in the job demands-resources model. ANXIETY STRESS AND COPING 33: 387-402.

78. Schwerdtfeger AR, Dick K (2019) Episodes of momentary resilience in daily life are associated with HRV reductions to stressful operations in firefighters: An ambulatory assessment approach using bayesian multilevel modeling. J Posit Psychol 14: 593-602.

79. Seaton EK, Zeiders KH (2021) Daily Racial Discrimination Experiences, Ethnic-Racial Identity, and Diurnal Cortisol Patterns Among Black Adults. CULTURAL DIVERSITY & ETHNIC MINORITY PSYCHOLOGY 27: 145-155.

80. Seltzer MM, Greenberg JS, Hong J, Smith LE, Almeida DM, et al. (2010) Maternal cortisol levels and behavior problems in adolescents and adults with ASD. J Autism Dev Disord 40: 457-469.

81. Shockley KM, Allen TD (2013) Episodic work-family conflict, cardiovascular indicators, and social support: An experience sampling approach. J Occup Health Psychol 18: 262-275.

82. Simon SG, Sloan RP, Thayer JF, Jamner LD (2021) Taking context to heart: Momentary emotions, menstrual cycle phase, and cardiac autonomic regulation. PSYCHOPHYSIOLOGY 58.

83. Sladek MR, Doane LD, Gonzales NA, Grimm KJ, Luecken LJ (2019) Latino adolescents' cultural values associated with diurnal cortisol activity. Psychoneuroendocrinology 109: 104403.

84. Sladek MR, Doane LD, Luecken LJ, Eisenberg N (2016) Perceived stress, coping, and cortisol reactivity in daily life: A study of adolescents during the first year of college. Biol Psychol 117: 8-15.

85. Sladek MR, Doane LD, Breitenstein RS (2020) Daily rumination about stress, sleep, and diurnal cortisol activity. Cogn Emot 34: 188-200.

86. Slatcher RB, Robles TF, Repetti RL, Fellows MD (2010) Momentary work worries, marital disclosure, and salivary cortisol among parents of young children. Psychosom Med 72: 887-896.

87. Smith PJ, Blumenthal JA, Babyak MA, Georgiades A, Sherwood A, et al. (2007) Ventricular ectopy: impact of self-reported stress after myocardial infarction. Am Heart J 153: 133-139.

88. Smyth J, Ockenfels MC, Porter L, Kirschbaum C, Hellhammer DH, et al. (1998) Stressors and mood measured on a momentary basis are associated with salivary cortisol secretion. Psychoneuroendocrinology 23: 353-370.

89. Smyth JM, Zawadzki MJ, Juth V, Sciamanna CN (2017) Global life satisfaction predicts ambulatory affect, stress, and cortisol in daily life in working adults. J Behav Med 40: 320-331.

90. Stawski RS, Cichy KE, Piazza JR, Almeida DM (2013) Associations among daily stressors and salivary cortisol: Findings from the National Study of Daily Experiences. Psychoneuroendocrinology 38: 2654-2665.

91. Stoffel M, Abbruzzese E, Rahn S, Bossmann U, Moessner M, et al. (2021) Covariation of psychobiological stress regulation with valence and quantity of social interactions in everyday life: disentangling intra- and interindividual sources of variation. JOURNAL OF NEURAL TRANSMISSION 128: 1381-1395.

92. Timmons AC, Han SC, Chaspari T, Kim Y, Pettit C, et al. (2019) Family-of-origin aggression, dating aggression, and physiological stress reactivity in daily life. Physiol Behav 206: 85-92.

93. Uchino BN, Berg CA, Smith TW, Pearce G, Skinner M (2006) Age-related differences in ambulatory blood pressure during daily stress: evidence for greater blood pressure reactivity with age. Psychol Aging 21: 231-239.

94. Vaessen T, Kasanova Z, Hernaus D, Lataster J, Collip D, et al. (2018) Overall cortisol, diurnal slope, and stress reactivity in psychosis: An experience sampling approach. Psychoneuroendocrinology 96: 61-68.

95. van Eck M, Berkhof H, Nicolson N, Sulon J (1996) The effects of perceived stress, traits, mood states, and stressful daily events on salivary cortisol. Psychosom Med 58: 447-458.

96. van Duin ED, Vaessen T, Kasanova Z, Viechtbauer W, Reininghaus U, et al. (2019) Lower cortisol levels and attenuated cortisol reactivity to daily-life stressors in adults with 22q11.2 deletion syndrome. Psychoneuroendocrinology 106: 85-94.

97. Volmer J, Fritsche A (2016) Daily negative work events and employees' physiological and psychological reactions. Front Psychol 7: 1711.

98. Wong JD, Mailick MR, Greenberg JS, Hong J, Coe CL (2014) Daily work stress and awakening cortisol in mothers of individuals with autism spectrum disorders or Fragile X syndrome. Fam Relat 63: 135-147.

99. Wong JD, Seltzer MM, Greenberg JS, Hong J, Almeida DM, et al. (2012) Stressful life events and daily stressors affect awakening cortisol level in midlife mothers of individuals with autism spectrum disorders. Aging Ment Health 16: 939-949.

100. Wong JD, Shobo Y (2016) The influences of employment status and daily stressors on physiological functioning in a sample of midlife and older adults. J Aging Hum Dev 83: 26-43.

101. Wong JD, Shobo Y (2017) The influences of daily stressors on morning cortisol levels in midlife and older retirees: The moderating roles of age and gender. J Aging Health 29: 858-879.

102. Wong JHK, Kelloway EK (2016) What Happens at Work Stays at Work? Workplace Supervisory Social Interactions and Blood Pressure Outcomes. J Occup Health Psychol 21: 133-141.

103. Wright L, Bukowski WM (2021) Gender is Key: Girls' and Boys' Cortisol Differs as a Factor of Socioeconomic Status and Social Experiences During Early Adolescence. Journal of Youth & Adolescence 50: 1281-1291.

104. Wrzus C, Mueller V, Wagner GG, Lindenberger U, Riediger M (2013) Affective and Cardiovascular Responding to Unpleasant Events From Adolescence to Old Age: Complexity of Events Matters. Dev Psychol 49: 384-397.
